# Synthesis of *C*-prenylated analogues of stilbenoid methyl ethers and their cyclic dihydrobenzopyranyl derivatives as potential anti-inflammatory agents†

**DOI:** 10.1039/d2ra00441k

**Published:** 2022-03-15

**Authors:** Hana Pizova, Milan Malanik, Karel Smejkal, Michal Oravec, Pavel Bobal

**Affiliations:** Department of Chemical Drugs, Faculty of Pharmacy, Masaryk University Palackeho tr. 1946/1 612 00 Brno Czech Republic bobalp@pharm.muni.cz; Department of Natural Drugs, Faculty of Pharmacy, Masaryk University Palackeho tr. 1946/1 612 00 Brno Czech Republic; Global Change Research Institute of the Czech Academy of Sciences Belidla 986/4a 603 00 Brno Czech Republic

## Abstract

An efficient and versatile synthesis of the naturally occurring *C*-prenylated stilbenoid methyl ethers and their synthetic analogues is presented. The synthesis represents a six step convergent process including an optimised *C*-prenylation method. Furthermore, during the demethylation process, six new dihydro-benzopyranyl derivatives were obtained and isolated.

## Abstract 2

An efficient and versatile synthesis of the naturally occurring *C*-prenylated stilbenoid methyl ethers and synthetic analogs has been developed.

## Graphical abstract

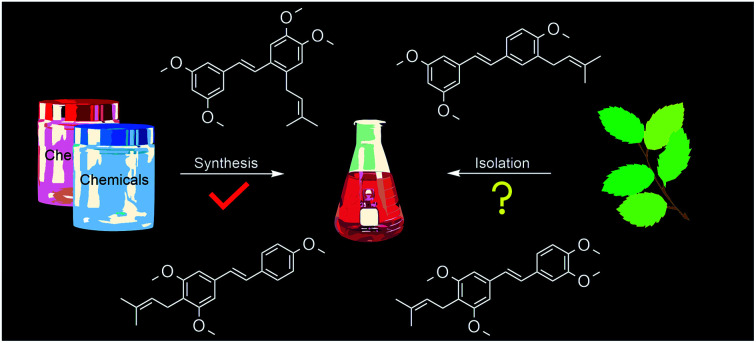

## Introduction

Stilbenoids are hydroxylated derivatives of stilbene and are a well-known class of naturally occurring phytochemicals. All of the stilbenoids contain a 1,2-diphenylethylene skeleton. They belong to a family of plant phenolics known for their structural complexity and diverse biological activities. Stilbenoids are classified as phytoalexins. They occur with a limited but heterogeneous distribution in plants.^1^ These compounds are stress metabolites produced in leaves and sapwood in response to pathogen infection.^2,3^ Although they play a crucial role as plant defence compounds, they possess high diversity of impacts on cellular and biological mechanisms affecting human health.

The most studied, *E*-resveratrol (3,4′,5-trihydroxystilbene), the non-flavonoid plant phenolic isolated from grapes or red wine, has unveiled strong antioxidant effects. The biological effects of this well-characterised stilbene include its role as an inducer of cell differentiation, a mediator of anti-inflammatory action and its anti-ageing properties.^4^ Furthermore, stilbenoids are known for their neuroprotective, cardioprotective, anti-inflammatory and anti-diabetic properties, and they could also find application in depigmentation and finally the prevention and treatment of cancer. These effects are thought to be mediated by several universal signalling pathways.^5^

Behind almost all of the other beneficial pharmacological effects of stilbenoids are their antioxidant and anti-inflammatory activities. There is a high number of research articles related to the anti-inflammatory activity of stilbenoids. A lot of them are highlighted in the review published recently.^6^ The most studied *E*-isomer of resveratrol, and its derivatives are potent inhibitors of cyclooxygenase-2, the critical enzyme for inflammation induction.^7,8^ Moreover, resveratrol also inhibits COX-2 gene expression.^9^

Prenylated stilbenoids synthesised in some plants and legumes exhibit plant pathogen defence properties and pharmacological activities with potential benefits to human health.^10^ Anti-inflammatory activity of over 400 prenylated phenols including stilbenoids are discussed in the recent review.^11^ Prenylated stilbenoids showed promising anti-inflammatory potential. In our recent studies, 11 natural and synthetic prenylated compounds were tested for their potential to inhibit the catalytic activity of cyclooxygenase-1 (COX-1), cyclooxygenase-2 (COX-2), and 5-lipoxygenase (5-LOX) *in vitro* in a cell-free assay using human recombinant enzymes. All of the tested structures have shown their anti-inflammatory activity. Our study shows that prenylation can favourably affect the activity and increase the anti-inflammatory potential of stilbenoids.^12^ These results encouraged us to develop reliable synthetic methods for achieving prenylated stilbenoids as potent multi-target anti-inflammatory compounds in acceptable yields.

**Fig. 1:**
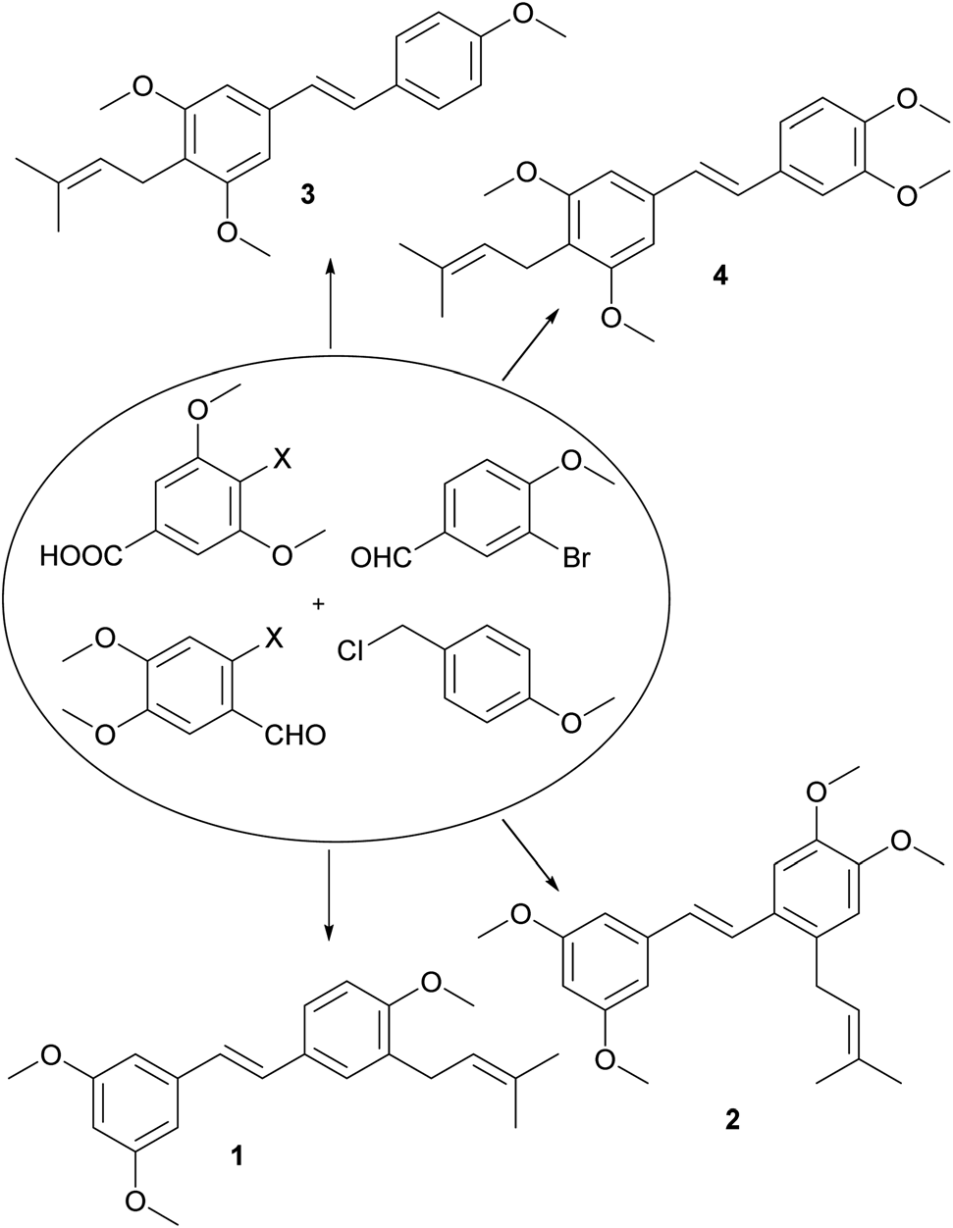
Selected natural and synthetic *C*-prenylated stilbenoids methyl ethers.

From a large amount of theoretically possible simple monomeric prenylated stilbenoid methyl ethers, we have selected four of them (Fig. 1). There is missing any information in the literature about compounds 1, 2 and 4, and compound 3 has been firstly isolated from the root of *Derris floribunda* and later from *Deguelia duckeana* but its synthesis has not been published yet.^13,14^

The aim of this work was to develop an efficient and practical procedure for the synthesis of a key *C*-prenylated stilbenoids from relatively simple and commercially available methoxylated aromatic aldehydes or acids (Fig. 1). We have selected the classic formation of olefin from plenty of synthetic procedures towards the stilbenoids by a condensation reaction. In our previous results published earlier, we prepared resveratrol methyl ether and its brominated derivative *via* Horner–Wadsworth–Emmons reaction^15,16^ of benzylic diethyl phosphonate and an aromatic aldehyde.^17^ In order to obtain *C*-prenylated stilbenoids first, we studied the reaction of resveratrol methyl ether with *n*-BuLi so-called *ortho*-lithiation followed by the addition of prenyl bromide. Unfortunately, we did not get satisfactory results. On the other hand, the direct lithiation of the brominated derivative of *E*-resveratrol methyl ether afforded a mixture of an alkylated product but with very low conversion. Therefore we carried out this type of bromine–lithium exchange in the presence of TMEDA.^18^ This experiment provided only traces of alkylated product and we isolated also debrominated product. When CuCN·2LiCl^19^ was used instead of TMEDA, conversion of prenylation raised to about 75% and we isolated desired *C*-prenylated resveratrol methyl ether as an *E*-isomer together with three other products *e.g.*, *C*-prenylated *Z*-isomer of resveratrol methyl ether, a product of allylic rearrangement and resveratrol itself. Similar results were obtained by using CuBr·DMS^20,21^ but the ratio of the desired product to rearranged one was more favorable.^12^

## Results and discussion

**Scheme 1:**
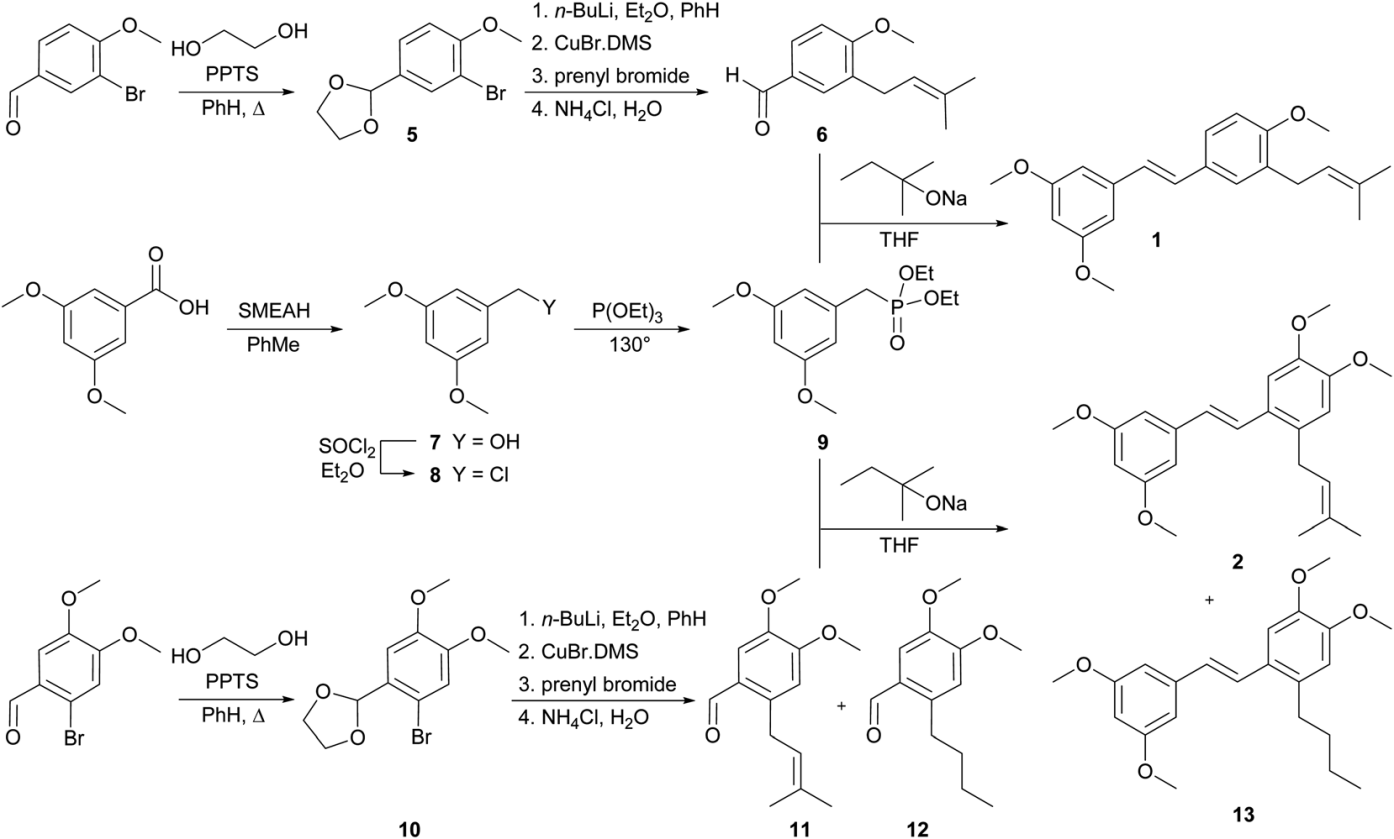
Synthesis of 3- and 2-prenylstilbenoids methyl ethers 1 and 2.

The synthetic route used to prepare the desired *C*-prenylated stilbenoid methyl ethers 1 and 2 is given in Scheme 1. From the two selected and tested methods of *C*-prenylation using CuBr·DMS^20,21^ described above, we have chosen a more convenient one, which does not require such a low temperature.^21^ The suitable substrate for prenylation 5 was prepared from commercially available 3-bromo-4-methoxybenzaldehyde with ethylene glycol and PPTS using a Dean–Stark adapter according to a modified procedure of Stephenson *et al.*^20^ Direct lithiation of dioxolane 5 was carried out with *n*-butyllithium to generate the anion followed by transmetalation with CuBr·DMS. The resulting cuprate was subsequently treated with prenyl bromide to yield prenylated dioxolane derivative, which was directly cleaved to aldehyde 6 during the aqueous work-up. In parallel, 3,5-dimethoxybenzoic acid was reduced with SMEAH at elevated temperature to alcohol 7, which was turned to dimethoxybenzyl chloride 8 with thionyl chloride.^22^ Chloride 8 was converted into diethyl phosphonate 9 by Michaelis–Arbuzov reaction.^23,24^ Finally, Horner–Wadsworth–Emmons reaction^15,16^ of diethyl phosphonate 9 and prenylated aldehyde 6 using sodium *tert*-pentoxide as base provide *C*-prenylated stilbenoid methyl ether 1 as an *E*-isomer in 64% yield. The condensation reaction with sodium hydride as a base provided similar results as with sodium *tert*-pentoxide but from a practical point of view, manipulation with sodium *tert*-pentoxide was much easier than with hydride. A similar procedure for *C*-prenylation of dioxolane 10 prepared from 2-bromo-4,5-dimethoxybenzaldehyde was applied. Surprisingly, together with expected prenylated aldehyde 11 also 2-butyl derivative 12 was formed. Despite the enormous efforts to separate derivatives 11 and 12, we were unable to do so. Therefore, the last coupling step was carried out by the same method with a mixture of aldehydes 11 and 12. After the reaction with diethyl phosphonate 9 the mixture of *E*-isomers of *C*-prenyl- and *C*-butylstilbenoid methyl ethers 2 and 13 was isolated. Our trials to separate compounds 2 and 13 were unsuccessful too.

**Scheme 2:**
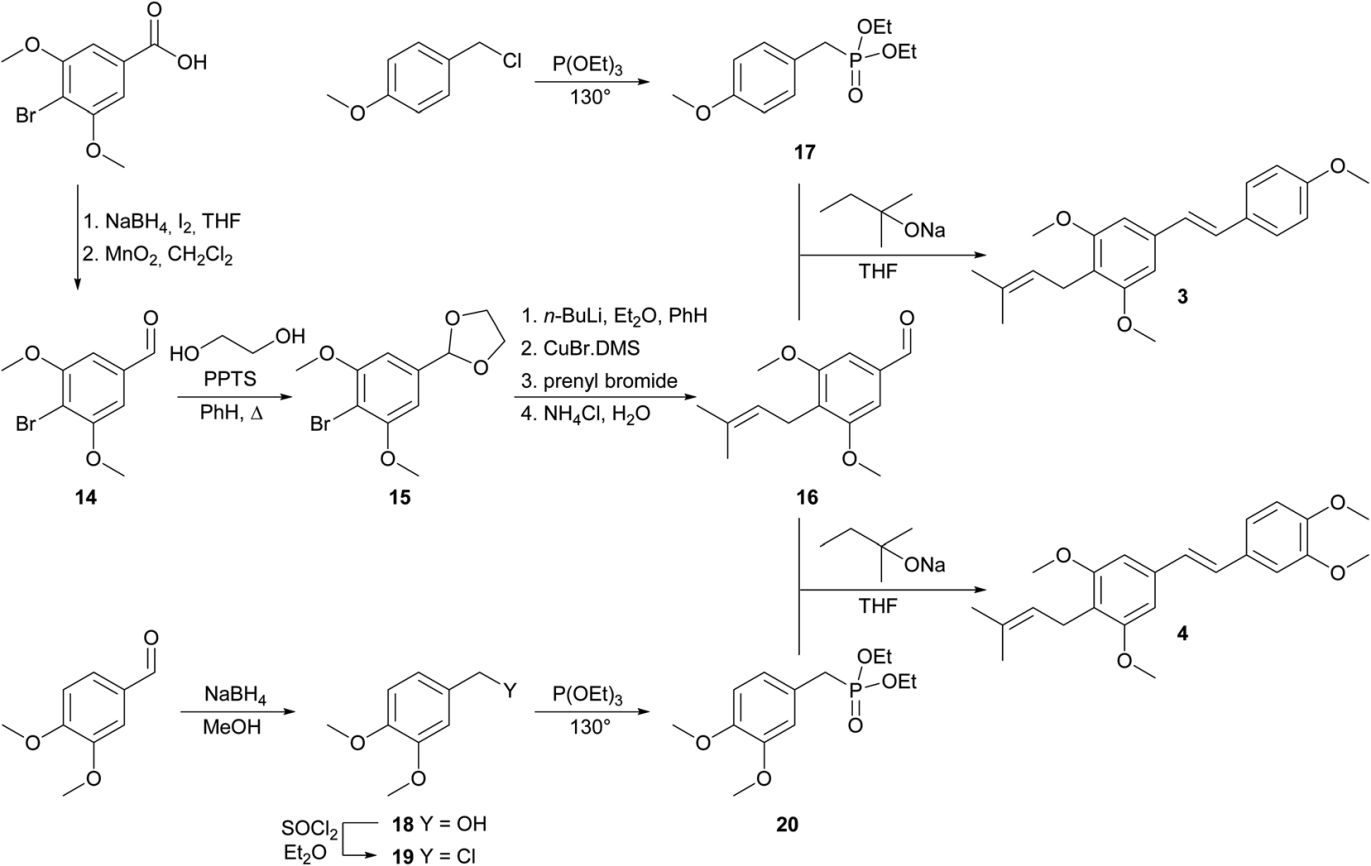
Synthesis of 4-prenylstilbenoids methyl ethers 3 and 4.

The synthesis of 4-prenylstilbenoids methyl ethers 3 and 4 is highlighted in Scheme 2. A similar approach has been selected starting from commercially available 4-bromo-3,5-dimethoxybenzoic acid. When SMEAH was used to reduce carboxylic function, as in the example described above, 4-bromo-3,5-dimethoxybenzyl alcohol was isolated, but it was contaminated with almost 30% of debrominated benzyl alcohol. For that reason, the reduction was carried out with diborane generated *in situ* from sodium borohydride, and iodine in THF.^25^ Prepared 4-bromo-3,5-dimethoxybenzyl alcohol was then oxidized to an aldehyde 14, which was converted to dioxolane 15 by the same method as described earlier.^20^*C*-Prenylation consisting of bromine–metal exchange using *n*-butyllithium followed by transmetalation with CuBr.DMS and treatment with prenyl bromide led after the aqueous work-up to the formation of *C*-prenylated aldehyde 16 in satisfactory yield. Diethyl phosphonate 17 was prepared from 4-methoxybenzyl chloride and triethyl phosphite at elevated temperature.^23,24^ The base catalysed condensation reaction provided 4-prenylstilbenoid methyl ether 3.^15,16^ For the synthesis of 4-prenylstilbenoid methyl ether 4 was required diethyl phosphonate 20 which was prepared from 3,4-dimethoxybenzaldehyde *via* reduction with sodium borohydride,^26^ chlorination with thionyl chloride^22^ and subsequent conversion under Michaelis–Arbuzov conditions.^23,24^ The final condensation to product 4 was performed similarly than for compound 3.^15,16^

**Scheme 3:**
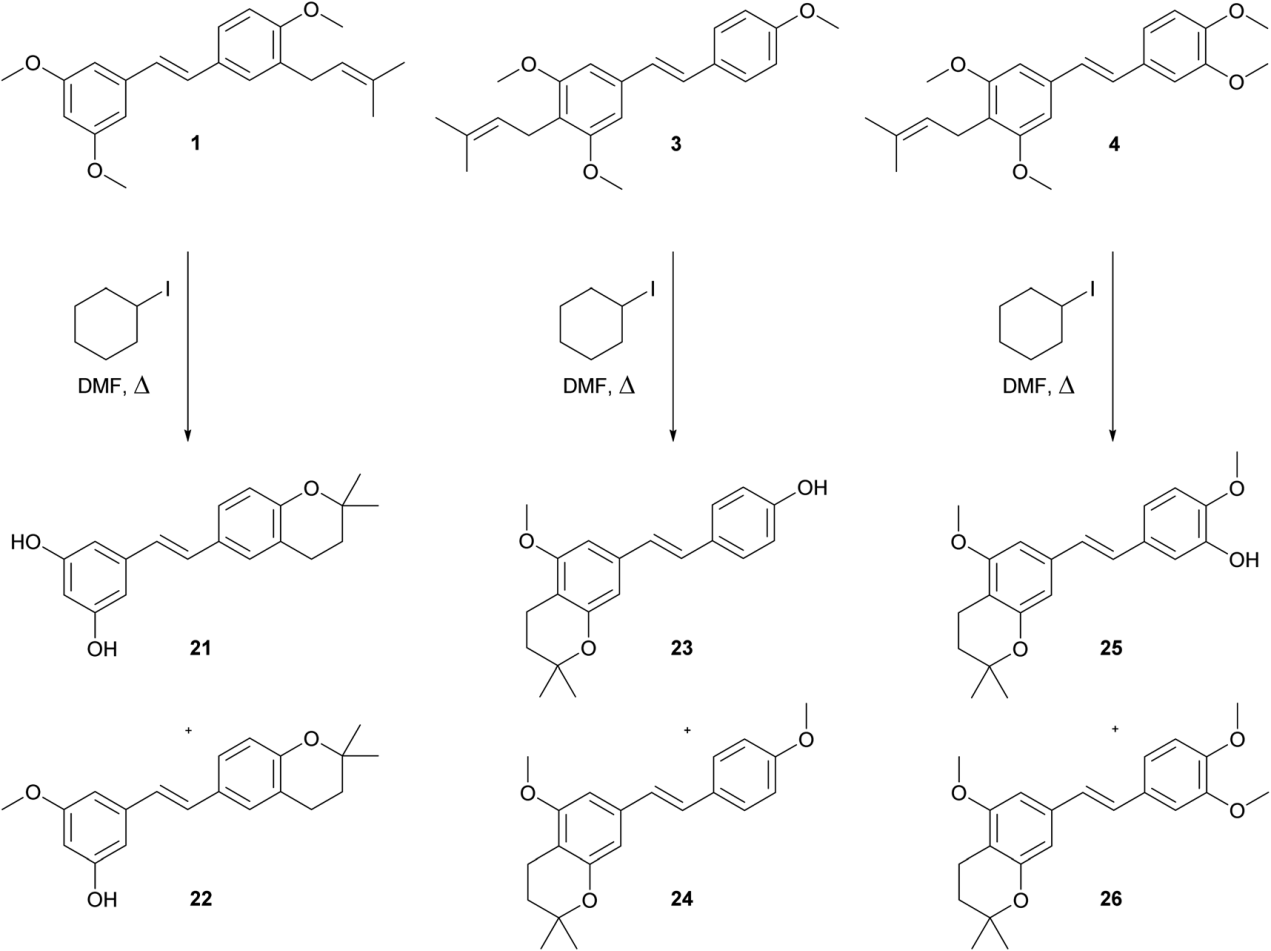
Formation of cyclic dihydrobenzopyranyl derivatives 21–26.

The demethylation procedure has been tested on synthesised compounds 1, 3 and 4. Although boron tribromide catalysed demethylation of trimethylresveratrol and dimethylpinosylvin led to the formation of desired stilbenoids,^27^ in case of our prenylated derivatives 1, 3 and 4 only the decomposition was observed. From all of the tested methods for demethylation of aryl methyl ethers,^27–31^ the best results were achieved by using iodocyclohexane in DMF under reflux conditions.^32^ During this reaction, hydrogen iodide slowly formed *in situ* cleaves the methyl ethers. The disadvantage of this method is that only traces of desired demethylated *C*-prenylated stilbenoids were isolated. The major products of this processes were dihydrobenzopyranyl derivatives 21–26 as shown on Scheme 3. The formation of cyclic compound 21–26 was probably caused by the strong acidity of formed hydrogen iodide and quite a high temperature of the reaction. The cyclization of *ortho*-prenylated phenols during Lewis acid catalysed demethylation has been reported in case of isoxanthohumol^28^ and during HCl catalysed deprotection of MOM-protected isobavachalcone.^33^

## Experimental

Experimental details for the chemical synthesis, IR, ^1^H and ^13^C NMR and MS spectra of the synthesised compounds are given in the ESI† section.

## Conclusions

In summary, efficient total synthesis of four *C*-prenylated stilbenoids methyl ethers has been successfully carried out. Their syntheses have not been published so far. Three of them are entirely new compounds. This convergent process comprises of usually six steps, including an optimised prenylation procedure and condensation reaction. The desired products were prepared in an overall 11–38% yield. In addition, after the process of demethylation, six new cyclic dihydrobenzopyranyl derivatives were prepared as well. The investigation of the anti-inflammatory activity of products 1–4, 21–26 is currently in progress.

## Author contributions

H. P. carried out the synthesis, characterisation of all intermediates and products and wrote the preliminary draft of the paper. P. B. was responsible for conceptualising the work in every step of the project and finalising the paper. K. S. and M. M. were responsible for the separation of complicated reaction mixtures. M. O. performed the MS studies of synthesised compounds.

## Conflicts of interest

There are no conflicts of interest to declare.

## Supplementary Material

RA-012-D2RA00441K-s001
