# Supplementary material for: Synthesis of C-prenylated analogues of stilbenoid methyl ethers and their cyclic dihydrobenzopyranyl derivatives as potential anti-inflammatory agents
Source: RSC Adv. 2022 Mar 15;12(13):8188–92. doi: 10.1039/d2ra00441k (PMC8982364; doi:10.1039/d2ra00441k)

Electronic Supplementary Material (ESI) for RSC Advances.
This journal is © The Royal Society of Chemistry 2022

Supporting Information

Synthesis of *C*-prenylated analogues of stilbenoid methyl ethers and their cyclic dihydrobenzopyranyl derivatives as potential anti-inflammatory agents

Hana Pizova,[a] Milan Malanik,[b] Karel Smejkal,[b] Michal Oravec,[c] and Pavel Bobal*[a]

Department of Chemical Drugs, Faculty of Pharmacy, Masaryk University, Palackeho tr. 1946/1, 612 00 Brno, Czech Republic.

****E-mail: bobalp@pharm.muni.cz***

Department of Natural Drugs, Faculty of Pharmacy, Masaryk University, Palackeho tr. 1946/1, 612 00 Brno, Czech Republic.

Global Change Research Institute of the Czech Academy of Sciences, Belidla 986/4a, 603 00 Brno, Czech Republic.

Contents

1. **General Information**
2. **General experimental procedures – characterization of products**
3. **Spectral characteristics**

1. General Information.

The reagents and solvents used for synthesis were purchased from Sigma-Aldrich and used as received, if not stated otherwise. Anhydrous reagents and solvents were absolutized as usual and distilled prior to use. 1D-NMR spectra were obtained on a JEOL ECZR-400 MHz instrument (Jeol Corp.). All NMR measurements were done at 25 °C. Chemical shifts δ are reported in parts per million (ppm) and *J* values in Hz. NMR spectra were acquired in $CDCl_3$, or DMSO-d_6. The signal of TMS or the residual solvent signals of $CDCl_3$, or DMSO-d_6 were used for reference. Norell StandartSeries™ 5 mm NMR tubes were utilized. MS analysis was performed on an LTQ Orbitrap XL high-resolution mass spectrometer (Thermo Fisher Scientific). Melting points were measured using a Böetius apparatus (Franz Küstner Nachf. KG) and were not corrected. Mass spectra were obtained using electrospray ionization (ESI) in the positive or negative ion mode. IR spectra were recorded on a SmartMIRacle ATR ZnSe for Nicolet Impact 410 FT-IR spectrometer (Thermo Scientific Corp.). Separation was performed using a semipreparative HPLC Young Lin 9100 (Young Lin, South Korea) instrument with an Ascentis RP-amide (250 mm × 10 mm, particle size 5 μm) column (Supelco, USA) with gradient elution with acetonitrile and H_2O, 67 % of acetonitrile in 0th minute to 72 % of acetonitrile in 20th minute. The flow rate was 5 mL/min, the column temperature was 40 °C,

and the separation was monitored using UV detection at 254 and 350 nm. For column chromatography Merck Kieselgel 60 silica gel (70-230 mesh particle size) was used. All fractions were monitored by silica gel thin-layer chromatography (TLC) plates (225 µm thickness, 60 A silica gel medium) with the detection of compounds with fluorescent light.

All of the synthetic steps are highlighted in Schemes 1 - 3.

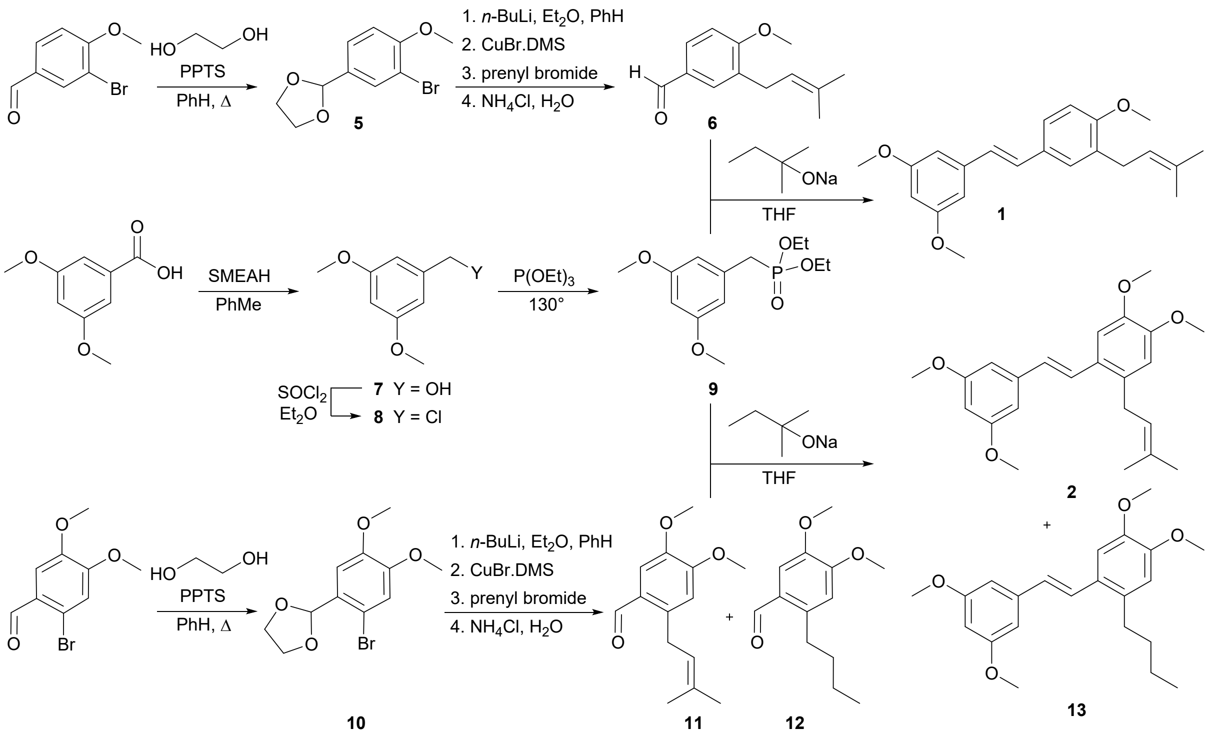

Scheme 1 Synthesis of 3- and 2-prenylstilbenoids methyl ethers **1** and **2**.

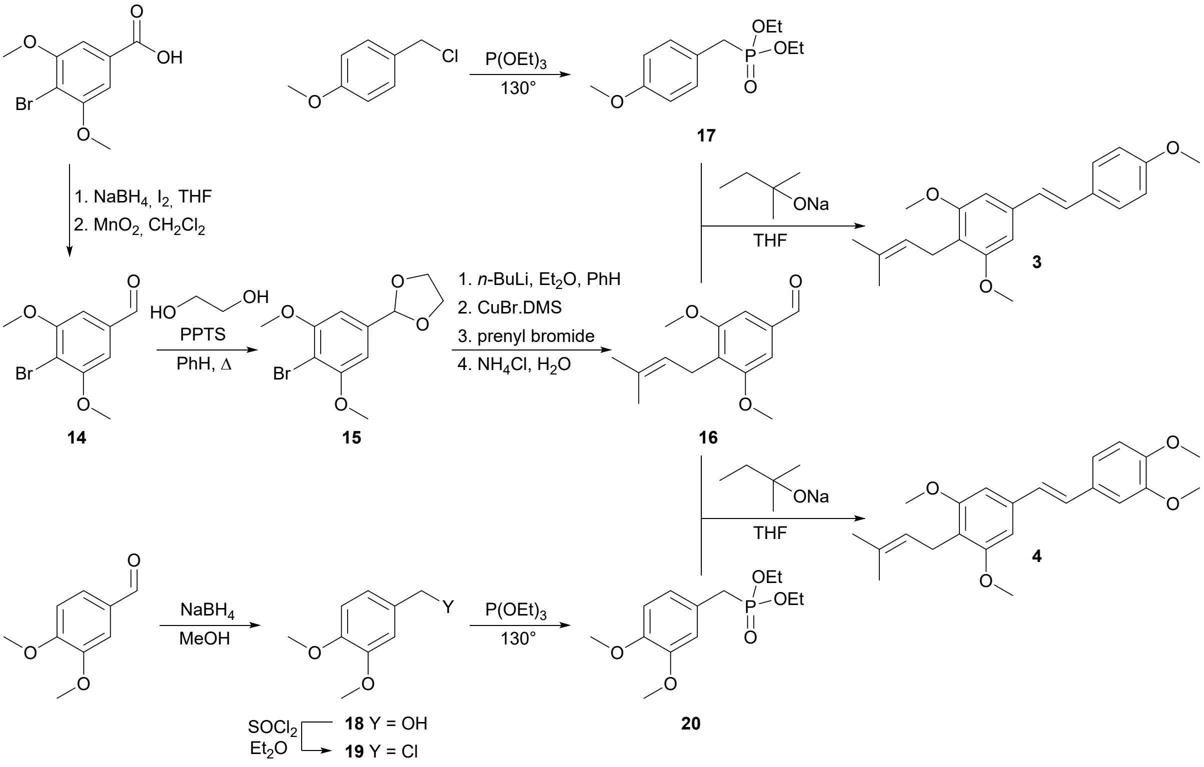

Scheme 2 Synthesis of 4-prenylstilbenoids methyl ethers **3** and **4**.

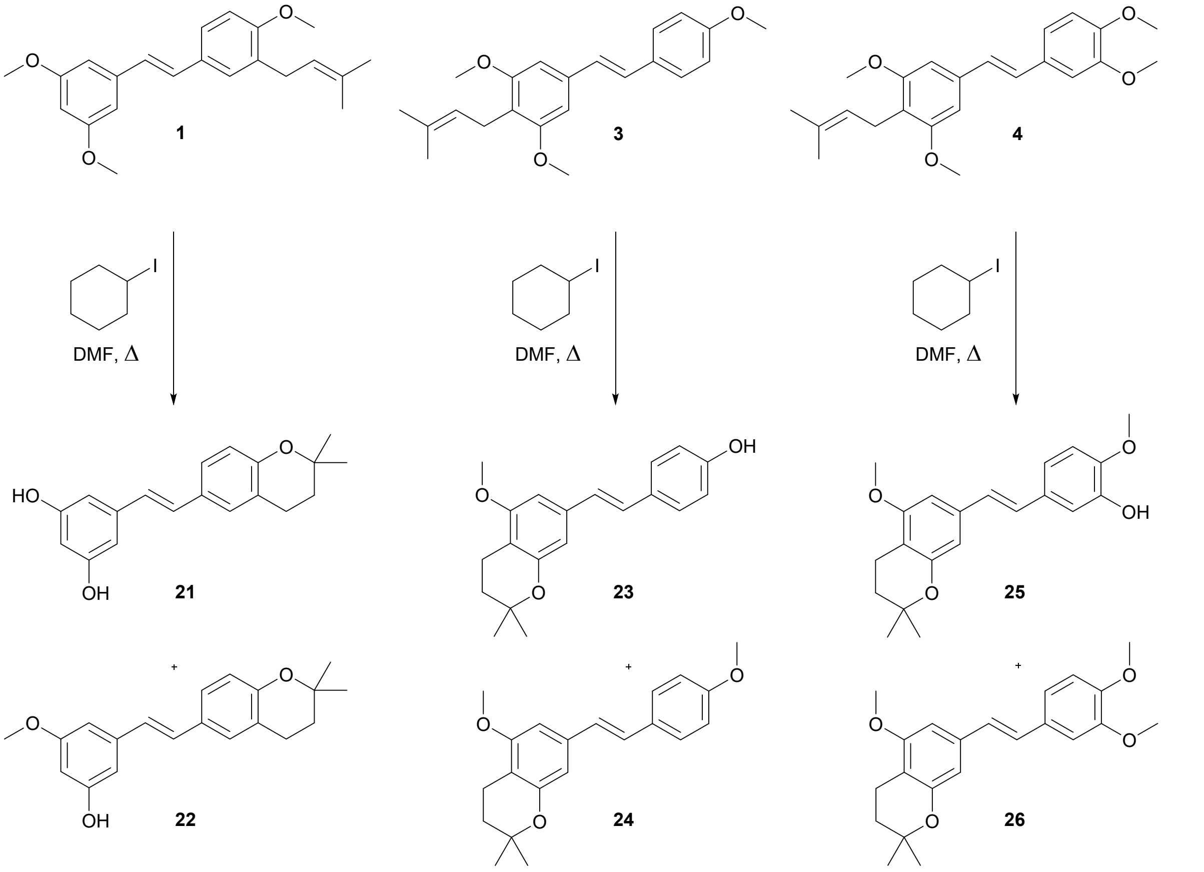

Scheme 3 Formation of cyclic dihydrobenzopyranyl derivatives **21** - **26**.

2. General experimental procedures – characterization of products

Reduction to alcohol

3,5-Dimethoxybenzyl alcohol 7

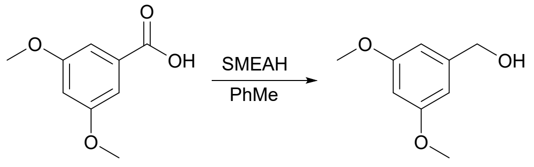

7

To a mixture of 5.00 g (0.0274 mol) of 3,5-dimethoxybenzoic acid in dry toluene (100 mL) 27.70 g (0.0892 mol) of 60% SMEAH was dropwise added. The reaction mixture was stirred at 80 °C 1.5 h under an atmosphere of argon. The reaction was monitored by thin-layer chromatography (ethyl acetate/*n*-hexane, 2/3). After the disappearance of the starting material, the mixture was cooled down to 0 °C, and water (75 mL) was carefully added. The reaction mixture was acidified with 1 M HCl and extracted three times with toluene (3×100 mL). The organic layer was separated, washed twice with water and dried with anhydrous $MgSO_4$. The solvent was evaporated under reduced pressure to produce 3.79 g (82 %) of white crystals of 3,5-dimethoxybenzyl alcohol (Mp 46-48 °C).[1] ^{1}H NMR (400 MHz, DMSO-d_6) δ 6.48 (d, $^4J_{HH}$ = 2.3 Hz, 2H), 6.34 (t, $^4J_{HH}$ = 2.3 Hz, 1H), 5.17 (t, $^3J_{HH}$ = 6.0 Hz, 1H), 4.43 (d, $^3J_{HH}$ = 6.0 Hz, 2H), 3.72 (s, 6H). ^{13}C NMR (101 MHz, DMSO-d_6) δ 160.3, 145.2, 104.0, 98.5, 62.8, 55.0.

3,4-Dimethoxybenzyl alcohol 18

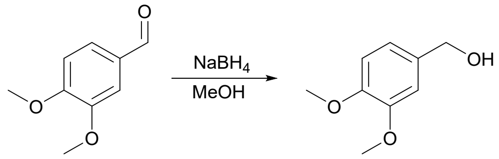

18

To the solution of 5.00 g (0.0301 mol) of 3,4-dimethoxybenzyl aldehyde was dissolved in MeOH (50 mL). To the solution, 3.50 g (0.0924 mol) of $NaBH_4$ was added. The reaction mixture was stirred under an inert atmosphere overnight. The reaction was monitored by thin-layer chromatography (3% MeOH in dichloromethane). To the reaction mixture, 2.5M NaOH (5 mL) was dropwise added, followed by water (20 mL). The mixture was extracted with chloroform (3×100 mL). The organic layer was dried with anhydrous $MgSO_4$, and the solvent was evaporated under reduced pressure to produce 4.22 g (83 %) of colourless oil of 3,4-dimethoxybenzyl alcohol.[2] ^{1}H NMR (400 MHz, $CDCl_3$) δ 6.93–6.80 (m, 3H), 4.59 (s, 2H), 3.87 (s, 3H), 3.86 (s, 3H), 2.13–1.97 (m, 1H). ^{13}C NMR (101 MHz, $CDCl_3$) δ 148.9, 148.4, 133.5, 119.3, 110.9, 110.3, 65.1, 55.8, 55.7.

4-Bromo-3,5-dimethoxybenzyl alcohol

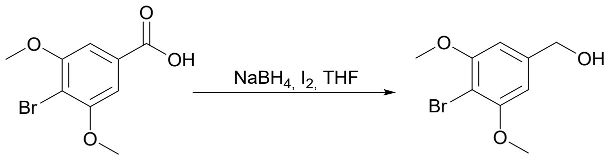

To the solution of 5.00 g (0.0215 mol) of 4-bromo-3,5-dimethoxybenzoic acid and 2.50 g (0.0661 mol) of $NaBH_4$ in dry THF (125 mL), the solution of 6.20 g (0.0244 mol) of iodine in dry THF (20 mL) was dropwise added at 0 °C. The reaction mixture was heating under reflux overnight. The reaction was monitored by thin-layer chromatography (3% MeOH in dichloromethane). To the reaction mixture, methanol (50 mL) was dropwise added, stirred 30 minutes and concentrated under reduced pressure. The rest was dissolved in a mixture of dichloromethane and 5% NaOH. The organic layer was separated, and the water layer was extracted three times with dichloromethane (3×100 mL). Organic layers were washed three times with $Na_2S_2O_3$ (3×50 mL) and dried with anhydrous $MgSO_4$. The solvent was evaporated under reduced pressure to produce 3.33 g (70 %) of white crystals of 4-bromo-3,5-dimethoxybenzyl alcohol (Mp 75-76 °C).[3] ^1H NMR (400 MHz, $CDCl_3$) δ 6.59 (s, 2H), 4.67 (s, 2H), 3.90 (s, 6H), 1.93 (br. s., 2H). ^{13}C NMR (101 MHz, $CDCl_3$) δ 157.1, 141.7, 102.9, 99.6, 65.1, 56.4.

4-Bromo-3,5-dimethoxybenzaldehyde 14

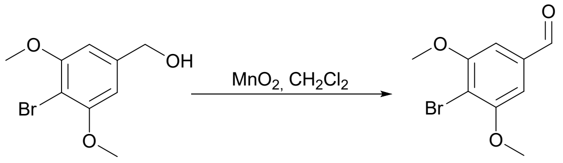

14

To a solution of 1.00 g (0.0040 mol) of 4-bromo-3,5-dimethoxybenzyl alcohol in dry dichloromethane (50 mL), 3.50 g (0.0405 mol) of MnO_2 was added. The reaction was stirred at room temperature under an atmosphere of inert gas for six days. The reaction was monitored by thin-layer chromatography (10% MeOH in dichloromethane). The reaction mixture was filtrated through Celite layer and washed with dichloromethane. The solvent was evaporated under reduced pressure to produce 0.82 g (83 %) of yellow crystals of 4-bromo-3,5-dimethoxybenzyl aldehyde (Mp 107-108 °C).[4] ^1H NMR (400 MHz, $CDCl_3$) δ 9.93 (s, 1H), 7.07 (s, 2H), 3.98 (s, 6H). ^{13}C NMR (101 MHz, $CDCl_3$) δ 191.1, 157.7, 136.2, 108.5, 105.1, 56.7.

Preparation of chlorides

3,5-Dimethoxybenzyl chloride 8

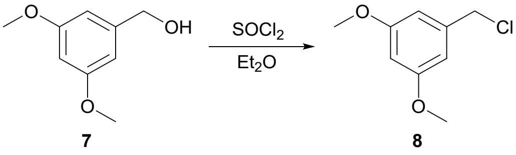

3.79 g (0.0225 mol) of 3,5-Dimethoxybenzyl alcohol was dissolved in dry diethyl ether (70 mL). The mixture was cooled in an ice bath, and DMF (1 mL) was added, followed by a mixture of $SOCl_2$ 5.36 g (0.0451 mol) in dry diethyl ether (10 mL). The reaction mixture was stirred at 25 °C overnight under a condenser equipped with calcium chloride closure. The reaction was monitored by thin-layer chromatography (ethyl acetate/*n*-hexane, 2/3). After the disappearance of the starting material, the mixture was cooled down, and water (50 mL) was slowly added and extracted three times with diethyl ether (3×50 mL). The organic layers were washed with water (50 mL), brine (50 mL) and dried with anhydrous $MgSO_4$. The solvent was evaporated under reduced pressure, and the product was purified with column chromatography (ethyl acetate/*n*-hexane, 2/3) to produce 3.08 g (73 %) of yellowish crystals of 3,5-dimethoxybenzyl chloride (Mp 44-45 °C).[1] ^{1}H NMR (400 MHz, $CDCl_3$) δ 6.55 (d, $^4J_{HH}$ = 2.3 Hz, 2H), 6.43 (t, $^4J_{HH}$ = 2.3 Hz, 1H), 4.53 (s, 2H), 3.81 (s, 6H); ^{13}C NMR (101 MHz, $CDCl_3$) δ 160.9, 139.5, 106.4, 100.4, 55.4, 46.3.

3,4-Dimethoxybenzyl chloride 19

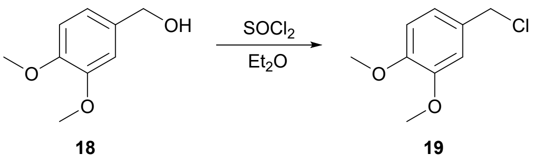

4.00 g (0.0238 mol) of 3,4-Dimethoxybenzyl alcohol was dissolved in dry diethyl ether (80 mL). The mixture was cooled in an ice bath, and DMF (1.18 mL) was added, followed by a mixture of $SOCl_2$ 5.66 g (0.0476 mol) in dry diethyl ether (10 mL). The reaction mixture was stirred at 25 °C overnight under a condenser equipped with calcium chloride closure. The reaction was monitored by thin-layer chromatography (ethyl acetate/*n*-hexane, 2/3). After the disappearance of the starting material, the mixture was cooled down, and water (50 mL) was slowly added and extracted three times with diethyl ether (3×50 mL). Organic layers were washed with water (50 mL), brine (50 mL) and dried with anhydrous $MgSO_4$. The solvent was evaporated under reduced pressure, and the product was purified with column chromatography (ethyl acetate/*n*-hexane, 2/3) to produce 3.55 g (80 %) of yellowish crystals of 3,4-dimethoxybenzyl chloride (Mp 50-54 °C).[5] ^{1}H NMR (400 MHz, $CDCl_3$) δ 6.98–6.89 (m, 2H), 6.83 (d, $^3J_{HH}$ = 7.9 Hz, 1H), 4.58 (s, 2H), 3.91 (s, 3H), 3.89 (s, 3H). ^{13}C NMR (101 MHz, $CDCl_3$) δ 149.0, 129.9, 121.1, 111.6, 110.9, 55.9, 46.7.

Preparation of phosphonates

Diethyl 3,5-dimethoxybenzylphosphonate 9

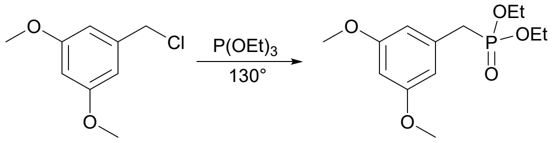

8 9

The reaction mixture of 2.70 g (0.0145 mol) of 3,5-dimethoxybenzyl chloride, 1.00 g (0.01445 mol) of triethyl phosphite and 0.20 g of potassium iodide was stirred at 130 °C 3h under condenser equipped with calcium chloride closure. After this time, same amounts of triethyl phosphite and potassium iodide were added, and a mixture was stirred again at 130 °C another 3 hours. Reaction was monitored by thin-layer chromatography (ethyl acetate/*n*-hexane, 2/3). Reaction mixture was cooled down and water (10 mL) was added and extracted three times with ethyl acetate (3×10 mL). Organic layer was washed with brine (10 mL) and dried over anhydrous $MgSO_4$. Solvent was evaporated under reduced pressure to produce 4.10 (98 %) of yellow oil of diethyl 3,5-dimethoxybenzylphosphonate.[6] ^{1}H NMR (400 MHz, $CDCl_3$) δ 6.45 (t, $^4J_{HH}$ = 2.3 Hz, 2H), 6.34 (q, $^4J_{HH}$ = 2.1 Hz, 1H), 4.08–3.97 (m, 4H), 3.77 (s, 6H), 3.09 (d, $^2J_{PH}$ = 22.0 Hz, 2H), 1.26 (t, $^3J_{HH}$ = 7.1 Hz, 6H). ^{13}C NMR (101 MHz, $CDCl_3$) δ 160.6 (d, $^4J_{PC}$ = 2.9 Hz), 133.5 (d, $^2J_{PC}$ = 8.7 Hz), 107.8 (d, $^3J_{PC}$ = 6.7 Hz), 99.0 (d, $^5J_{PC}$ = 2.8 Hz), 62.2 (d, $^2J_{POC}$ = 6.7 Hz), 55.3, 33.9 (d, $^1J_{PC}$ = 137.8 Hz), 16.4 (d, $^3J_{POCC}$ = 5.8 Hz).

Diethyl 3,4-dimethoxybenzylphosphonate 20

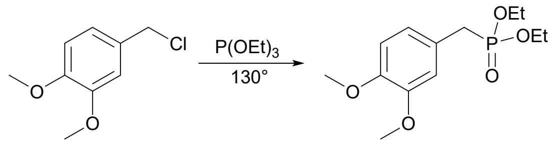

19 20

Reaction mixture of 2.00 g (0.0107 mol) of 3,4-dimethoxybenzyl chloride, 1.78 g (0.0107 mol) of triethyl phosphite and 0.14 g of potassium iodide was stirred at 130 °C 3h under condenser equipped with calcium chloride closure. After this time, the same amounts of triethyl phosphite and potassium iodide were added, and mixture was stirred again at 130 °C another 3 hours. Reaction was monitored by thin-layer chromatography (ethyl acetate/*n*-hexane, 2/3). Reaction mixture was cooled down and water (10 mL) was added and extracted three times with ethyl acetate (3×10 mL). Organic layer was washed with brine (10 mL) and dried over anhydrous $MgSO_4$. Solvent was evaporated under reduced pressure to produce 2.86 (92 %) of yellow oil of diethyl 3,4-dimethoxybenzylphosphonate.[7] ^{1}H NMR (400 MHz, $CDCl_3$) δ 6.87–6.74 (m, 3H), 4.05–3.94 (m, 4H), 3.86 (s, 3H), 3.84 (s, 3H), 3.08 (d, $^2J_{HP}$ = 21.3 Hz, 2H), 1.23 (t, $^3J_{HH}$ = 7.1 Hz, 6H). ^{13}C NMR (101 MHz, $CDCl_3$) δ 148.7 (d, $^5J_{PC}$ = 2.9 Hz), 147.9 (d, $^4J_{PC}$ = 3.9 Hz), 123.7 (d, $^2J_{PC}$ = 9.6 Hz), 121.8 (d, $^3J_{PC}$ = 7.7 Hz), 112.7 (d, $^3J_{PC}$ = 5.8 Hz), 111.1 (d, $^4J_{PC}$ = 2.9 Hz), 62.1 (d, $^2J_{POC}$ = 6.7 Hz), 55.8, 33.1 (d, $^1J_{PC}$ = 138.7 Hz), 16.3 (d, $^3J_{POCC}$ = 5.8 Hz).

Diethyl 4-methoxybenzylphosphonate 17

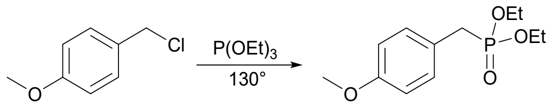

17

Reaction mixture of 1.00 g (0.0064 mol) of 4-methoxybenzyl chloride, 1.06 g (0.0064 mol) of triethyl phosphite and 0.10 g of potassium iodide was stirred at 130 °C 3h under condenser equipped with calcium chloride closure. After this time, same amounts of triethyl phosphite and potassium iodide were added, and mixture was stirred again at 130 °C another 3 hours. Reaction was monitored by thin-layer chromatography (ethyl acetate/*n*-hexane, 2/3). Reaction mixture was cooled down and water (10 mL) was added and extracted three times with ethyl acetate (3×10 mL). Organic layer was washed with brine (10 mL) and dried over anhydrous $MgSO_4$. Solvent was evaporated under reduced pressure to produce 1.63 (99 %) of colourless oil of diethyl 4-methoxybenzylphosphonate.[6] ^{1}H NMR (400 MHz, $CDCl_3$) δ 7.22–7.17 (m, 2H), 6.83 (d, $^3J_{HH}$ = 8.1 Hz, 2H), 4.04–3.93 (m, 4H), 3.77 (s, 3H), 3.07 (d, $^2J_{HP}$ = 21.2 Hz, 2H), 1.25–1.20 (m, 6H). ^{13}C NMR (101 MHz, $CDCl_3$) δ 158.5 (d, $^5J_{PC}$ = 2.9 Hz), 130.6 (d, $^3J_{PC}$ = 6.7 Hz), 123.3 (d, $^2J_{PC}$ = 8.7 Hz), 113.9 (d, $^4J_{PC}$ = 1.9 Hz), 61.9 (d, $^2J_{POC}$ = 6.7 Hz), 55.2, 32.7 (d, $^1J_{PC}$ = 139.7 Hz), 16.3 (d, $^3J_{POCC}$ = 5.8 Hz).

Protection of aldehydes

2-(3-Bromo-4-methoxyphenyl)-1,3-dioxolane 5

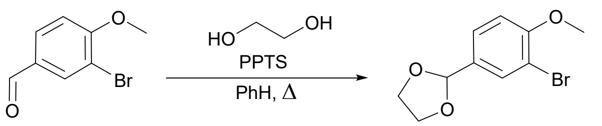

5

To a solution of 20.00 g (0.0930 mol) of 3-bromo-4-methoxybenzaldehyde in benzene (200 mL), 28.90 g (0.4650 mol) of ethylene glycol and 1.70 g (0.0070 mol) of pyridinium *p*-toluenesulfonate were added. The reaction mixture was heated under reflux with Dean-Stark equipment for 48 hours. The reaction was monitored by ^{1}H NMR. The reaction mixture was washed with water (2×100 mL), saturated aqueous solution of $NaHCO_3$ (100 mL) and brine (100 mL). The organic layer was dried over anhydrous $MgSO_4$ and solvent was evaporated under reduced pressure to produce 22.17 g (92 %) of white crystals of 2-(3-bromo-4-methoxyphenyl)-1,3-dioxolan dioxolan (Mp 28-30 °C).[8] ^{1}H NMR (400 MHz, $CDCl_3$) δ 7.68 (d, $^{3}J_{HH}$ = 2.1 Hz, 1H), 7.38 (dd, $^{3}J_{HH}$ = 8.5, $^{4}J_{HH}$ = 2.0 Hz, 1H), 6.89 (d, $^{3}J_{HH}$ = 8.5 Hz, 1H), 5.74 (s, 1H), 4.16–4.07 (m, 2H), 4.07–3.98 (m, 2H), 3.90 (s, 3H). ^{13}C NMR (101 MHz, $CDCl_3$) δ 156.4, 131.53, 131.48, 126.9, 111.6, 111.4, 102.7, 65.2, 56.3.

2-(4-Bromo-3,5-dimethoxyphenyl)-1,3-dioxolane 15

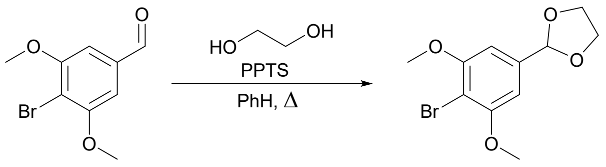

14 15

To a solution of 0.50 g (0.0020 mol) of 4-bromo-3,5-dimethoxybenzaldehyde in benzene (20 mL), 0.76 g (0.0122 mol) of ethylene glycol and 0.04 g (0.1667 mmol) of pyridinium *p*-toluenesulfonate were added. The reaction mixture was heated under reflux with Dean-Stark equipment for 48 hours. The reaction was monitored by ^{1}H NMR. The reaction mixture was washed with water (2×20 mL), saturated aqueous solution of $NaHCO_3$ (20 mL) and brine (20 mL). The organic layer was dried over anhydrous $MgSO_4$, and the solvent was evaporated under reduced pressure to produce 0.52 g (88 %) of white crystals of 2-(4-bromo-3,5-dimethoxyphenyl)-1,3-dioxolan (Mp 55-56 °C).[9] ^{1}H NMR (400 MHz, $CDCl_3$) δ 6.72 (s, 2H), 5.79 (s, 1H), 4.16–4.11 (m, 2H), 4.10–4.03 (m, 2H), 3.92 (s, 6H). ^{13}C NMR (101 MHz, $CDCl_3$) δ 157.0, 138.5, 103.1, 102.5, 101.4, 65.2, 56.5.

2-(2-Bromo-4,5-dimethoxyphenyl)-1,3-dioxolane 10

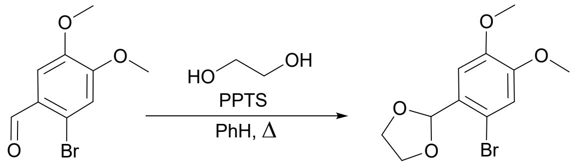

10

To a solution of 4.44 g (0.0181 mol) of 2-bromo-4,5-dimethoxybenzaldehyde in benzene (100 mL), 6.75 g (0.1087 mol) of ethylene glycol and 0.37 g (0.0014 mol) of pyridinium *p*-toluenesulfonate were added. The reaction mixture was heated under reflux with Dean-Stark equipment for 48 hours. The reaction was monitored by ^{1}H NMR. The reaction mixture was washed with water (2×50 mL), saturated aqueous solution of $NaHCO_3$ (50 mL) and brine (50 mL). The organic layer was dried over anhydrous $MgSO_4$, and the solvent was evaporated under reduced pressure to produce 4.62 g (88 %) of white crystals of 2-(2-bromo-4,5-dimethoxyphenyl)-1,3-dioxolan (Mp 108-110 °C).[10] ^{1}H NMR (400 MHz, $CDCl_3$) δ 7.11 (s, 1H), 7.01 (s, 1H), 5.99 (s, 1H), 4.20–4.13 (m, 2H), 4.11–4.03 (m, 2H), 3.89 (s, 3H), 3.87 (s, 3H). ^{13}C NMR (101 MHz, $CDCl_3$) δ 150.2, 148.4, 128.2, 115.4, 113.3, 110.1, 102.6, 65.3, 56.2, 56.0.

Prenylation procedure

4-Methoxy-3-(3-methylbut-2-en-1-yl)benzaldehyde 6

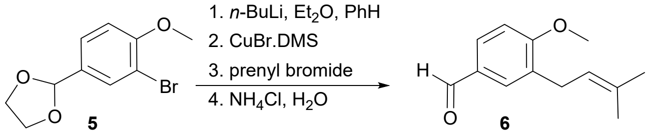

5.00 g (0.0190 mol) of 2-(3-Bromo-4-methoxyphenyl)-1,3-dioxolane was dissolved in a mixture of dry diethyl ether (80 mL) and anhydrous benzene (30 mL), and ground 5Å molecular sieves (2.50 g) were added. The reaction mixture was cooled to 0° C, and 9.20 mL (0.0230 mol) of 2.5 M *n*-BuLi in *n*-hexane was dropwise added. This mixture was stirred 30 minutes at room temperature and again cooled down to 0 °C, and 1.98 g (0.0097 mol) of CuBr·DMS was added. The reaction mixture was stirred at room temperature. After one hour, 3.43 g (0.0230 mol) of prenyl bromide was dropwise added. The reaction mixture was stirred for 3 hours, and saturated aqueous solution of NH_4Cl (50 mL) was carefully added, followed by H_2O (25 mL) and extracted to diethyl ether (3×50 mL). The organic layer was washed with 1M HCl (2×20 mL), dried over anhydrous $MgSO_4$ and solvent was evaporated under reduced pressure. The product was purified by column chromatography (ethyl acetate/*n*-hexane, 1/1) to produce 2.02 g (51 %) of 4-methoxy-3-(3-methylbut-2-en-1-yl) benzaldehyde as a colourless oil.[11] ^{1}H NMR (400 MHz, $CDCl_3$) δ 9.87 (s, 1H), 7.72 (dd, $^3J_{HH}$ = 8.5, $^4J_{HH}$ = 2.1 Hz, 1H), 7.68 (d, $^4J_{HH}$ = 2.3 Hz, 1H), 6.95 (d, $^3J_{HH}$ = 8.7 Hz, 1H), 5.33–5.28 (m, 1H), 3.93 (s, 3H), 3.35 (d, $^3J_{HH}$ = 7.3 Hz, 2H), 1.78–1.75 (m, 3H), 1.71 (s, 3H). ^{13}C NMR (101 MHz, $CDCl_3$) δ 191.3, 162.4, 133.5, 131.1, 130.6, 130.3, 129.6, 121.2, 109.9, 55.7, 28.2, 25.8, 17.8.

3,5-Dimethoxy-4-(3-methylbut-2-en-1-yl)benzaldehyde 16

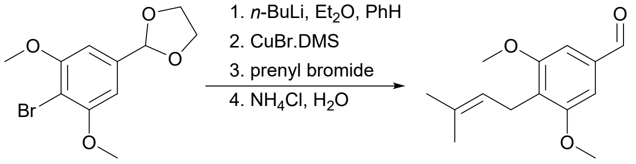

1.00 g (3.4588 mmol) of 2-(4-Bromo-3,5-dimethoxyphenyl)-1,3-dioxolane was dissolved in a mixture of dry diethyl ether (16 mL) and anhydrous benzene (6 mL), and ground 5Å molecular sieves (0.5 g) were added. The reaction mixture was cooled to 0° C, and 1.80 mL (4.4964 mmol) of 2.5 M *n*-BuLi in *n*-hexane was dropwise added. This mixture was stirred for 30 minutes at room temperature and again cooled down to 0 °C, and 0.36 g (1.7294 mmol) of CuBr·DMS was added. The reaction mixture was stirred at room temperature. After one hour, 0.67 g (4.4964 mmol) of prenyl bromide was dropwise added. The reaction mixture was stirred for 3 hours, and saturated aqueous solution of NH_4Cl (10 mL) was carefully added, followed by H_2O (5 mL) and extracted to diethyl ether (3×20 mL). The organic layer was washed with 1M HCl (2×10 mL), dried over anhydrous $MgSO_4$, and the solvent was evaporated under reduced pressure. The product was purified by column chromatography (ethyl acetate/*n*-hexane, 1/1) to produce 0.38 g (45 %) of 3,5-dimethoxy-4-(3-methylbut-2-en-1-yl) benzaldehyde as a colourless oil. IR (ATR) ν_{max}/cm^{-1} 2978, 2909, 1682, 1597, 1581, 1496, 1456, 1441, 1371, 1332, 1250, 1202, 1114, 1056, 1025, 968, 843, 810, 781. ^{1}H NMR (400 MHz, $CDCl_3$) δ 9.90

(s, 1H), 7.07 (s, 2H), 5.20–5.12 (m, 1H), 3.90 (s, 6H), 3.40 (d, $^{3}J_{HH}$ = 7.3 Hz, 2H), 1.78 (s, 3H), 1.67 (s, 3H). ^{13}C NMR (101 MHz, $CDCl_3$) δ 191.9, 158.4, 135.3, 132.2, 125.9, 121.3, 105.0, 55.9, 25.8, 22.8, 17.8. HRMS *m/z* calcd for $C_{14}H_{19}O_3$: 235.1329; found: $[M + H]^+$ 235.1321.

3,5-Dimethoxy-2-(3-methylbut-2-en-1-yl)benzaldehyde 11 and 2-butyl-3,5-dimethoxy-benzaldehyde 12

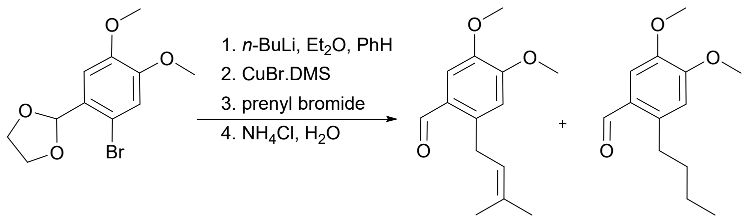

10 **11** **12**

3.00 g (0.0104 mol) of 2-(2-Bromo-4,5-dimethoxyphenyl)-1,3-dioxolane was dissolved in a mixture of dry diethyl ether (40 mL) and anhydrous benzene (20 mL), and ground 5Å molecular sieves (1.50 g) were added. The reaction mixture was cooled to 0° C, and 4.98 mL (0.0125 mol) of 2.5 M *n*-BuLi in *n*-hexane was dropwise added. This mixture was stirred for 30 minutes at room temperature and again cooled down to 0 °C, and 1.07 g (0.0052 mol) of CuBr·DMS was added. The reaction mixture was stirred at room temperature. After one hour, 1.86 g (0.0125 mol) of prenyl bromide was added dropwise. The reaction mixture was stirred for 3 hours, and saturated aqueous solution of NH_4Cl (20 mL) was carefully added, followed by H_2O (10 mL) and extracted to diethyl ether (3×20 mL). The organic layer was washed with 1M HCl (2×10 mL), dried over anhydrous $MgSO_4$ and solvent was evaporated under reduced pressure. Product was purified by column chromatography (ethyl acetate/*n*-hexane, 1/1) to produce 0.52 g (21 %) of mixture of yellow oil of 4,5-dimethoxy-2-(3-methylbut-2-en-1-yl) benzaldehyde and 2-butyl-4,5-dimethoxy benzaldehyde. ^{1}H NMR (400 MHz, $CDCl_3$) δ 10.23 (s, 1H), 10.21 (s, 1H), 7.38 (s, 2H), 6.72 (s, 1H), 6.70 (s, 1H), 5.29–5.22 (m, 1H), 3.96–3.95 (m, 6H), 3.92 (s, 6H), 3.69 (d, $^{3}J_{HH}$ = 6.7 Hz, 2H), 3.00–2.92 (m, 2H), 1.77–1.70 (m, 6H), 1.67–1.57 (m, 2H), 1.47–1.36 (m, 2H), 0.95 (t, $^{3}J_{HH}$ = 7.3 Hz, 3H). ^{13}C NMR (101 MHz, $CDCl_3$) δ 189.7, 153.8, 147.5, 141.4, 127.8, 126.5, 121.7, 112.6, 110.4, 56.0, 55.9, 55.7, 35.4, 31.3, 25.8, 22.5, 17.8, 13.9.

Preparation of stilbenes

1,3-Dimethoxy-5-[(*E*)-2-[4-methoxy-3-(3-methylbut-2-en-1-yl)phenyl]ethenyl]benzene 1

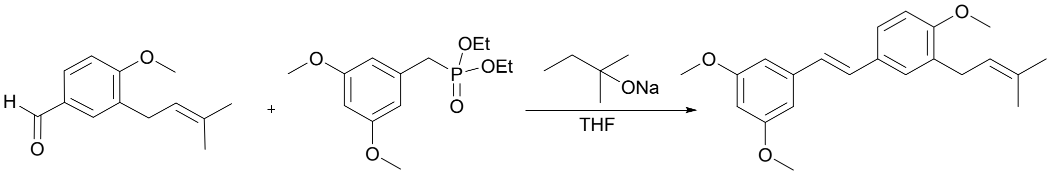

6 **9** **1**

To the solution of 1.35 g (0.0122 mol) of sodium *tert*-pentoxide in dry THF (20 mL), the solution of 0.71 g (2.4470 mmol) of diethyl 3,5-dimethoxybenzylphosphonate and 0.50 g (2.4470 mmol) of 3-(3-methylbut-2-en-1-yl)-4-methoxybenzaldehyde in dry THF (5 mL) was dropwise added. Reaction mixture was stirred at reflux under an inert atmosphere. The reaction was monitored by thin-layer chromatography (ethyl acetate/*n*-hexane, 1/4). To the reaction mixture was water (15 mL) added and extracted three times into ethyl acetate (3×20 mL). The organic layer was washed with brine (20 mL) and dried over anhydrous $MgSO_4$. The solvent was evaporated under reduced pressure. The product was purified by column chromatography (ethyl acetate/*n*-hexane, 1/4) to produce 1.07 g (64 %) of 1,3-dimethoxy-5-[(*E*)-2-[4-methoxy-3-(3-methylbut-2-en-1-yl)phenyl]ethenyl]benzene as yellow oil.[12] IR (ATR) ν_{max}/cm^{-1} 2931, 1593, 1500, 1459, 1253, 1204, 1154, 1066, 1032, 860, 827. ^{1}H NMR (400 MHz, $CDCl_3$) δ 7.37–7.30 (m, 2H), 7.06 (d, $^{3}J_{HH}$ = 16.2 Hz, 1H), 6.91 (d, $^{3}J_{HH}$ = 16.2 Hz, 1H), 6.85 (d, $^{3}J_{HH}$ = 8.1 Hz, 1H), 6.68 (d, $^{4}J_{HH}$ = 2.2 Hz, 2H), 6.39 (t, $^{4}J_{HH}$ = 2.1 Hz, 1H), 5.39–5.31 (m, 1H), 3.89–3.83 (m, 9H), 3.36 (d, $^{3}J_{HH}$ = 7.2 Hz, 2H), 1.79–1.78 (m, 6H). ^{13}C NMR (101 MHz, $CDCl_3$) δ 160.9, 157.2, 139.8, 132.6, 130.3, 129.5, 129.1, 127.6, 126.2, 125.4, 122.3, 110.3, 104.2, 99.5, 55.4, 55.3, 28.5, 25.8, 17.8. HRMS *m/z* calcd for $C_{22}H_{27}O_3$: 339.1955; found: $[M + H]^+$ 339.1955.

1,3-Dimethoxy-5-[(*E*)-2-(4-methoxyphenyl)ethenyl]-2-(3-methylbut-2-en-1-yl)benzene 3

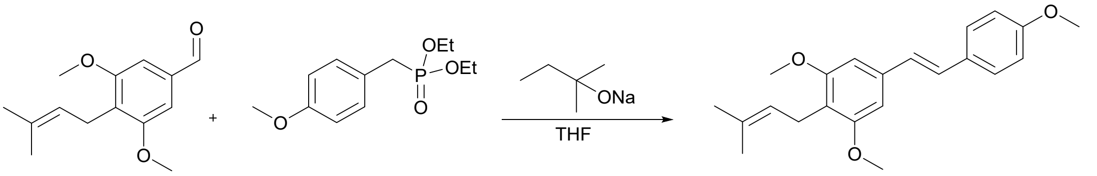

16 **17** **3**

To the solution of 588.00 mg (5.3350 mmol) of sodium *tert*-pentoxide in dry THF (5 mL), the solution of 250.00 mg (1.0671 mmol) of diethyl 4-methoxybenzylphosphonate and 276.00 mg (1.0671 mmol) of 4-(3-methylbut-2-en-1-yl)-3,5-dimethoxybenzaldehyde in dry THF (10 mL) was dropwise added. The reaction mixture was stirred at reflux under an inert atmosphere. The reaction was monitored by thin-layer chromatography (ethyl acetate/*n*-hexane, 1/4). To the reaction mixture was water (10 mL) added and extracted three times into ethyl acetate (3×20 mL). The organic layer was washed with brine (20 mL) and dried over anhydrous $MgSO_4$. Solvent was evaporated under reduced pressure. The product was purified by column chromatography (ethyl acetate/*n*-hexane, 1/4) to produce 100.00 mg (28 %) of 1,3-dimethoxy-5-[(*E*)-2-(4-methoxyphenyl)ethenyl]-2-(3-methylbut-2-en-1-yl)benzene as yellow oil.[13] IR

(ATR) ν_{max}/cm^{-1} 2924, 2844, 1585, 1509, 1454, 1415, 1244, 1171, 1108, 1030, 955, 824. ^1H NMR (400 MHz, $CDCl_3$) δ 7.46 (d, J = 8.7 Hz, 2 H), 7.03 (d, $^3J_{HH}$ = 16.0 Hz, 1H), 6.95 (d, $^3J_{HH}$ = 16.0 Hz, 1H), 6.91 (d, J = 8.7 Hz, 2 H), 6.59 (s, 2 H), 5.20 (t, J = 7.1 Hz, 1 H), 3.88 (s, 6 H), 3.84 (s, 3 H), 3.35 (d, J = 6.9 Hz, 2 H), 1.78 (s, 3 H), 1.67 (s, 3 H). ^{13}C NMR (101 MHz, $CDCl_3$) δ 159.2, 158.1, 136.4, 131.2, 130.1, 127.6, 127.5, 127.1, 122.8, 117.9, 114.1, 102.0, 55.8, 55.3, 25.8, 22.3, 17.7. HRMS *m/z* calcd for $C_{22}H_{27}O_3$: 339.1955; found: $[M + H]^+$ 339.1954.

1-[(*E*)-2-(3,5-dimethoxyphenyl)ethenyl]-4,5-dimethoxy-2-(3-methylbut-2-en-1-yl)benzene 2 and 1-butyl-2-[(*E*)-2-(3,5-dimethoxyphenyl)ethenyl]-4,5-dimethoxybenzene 13

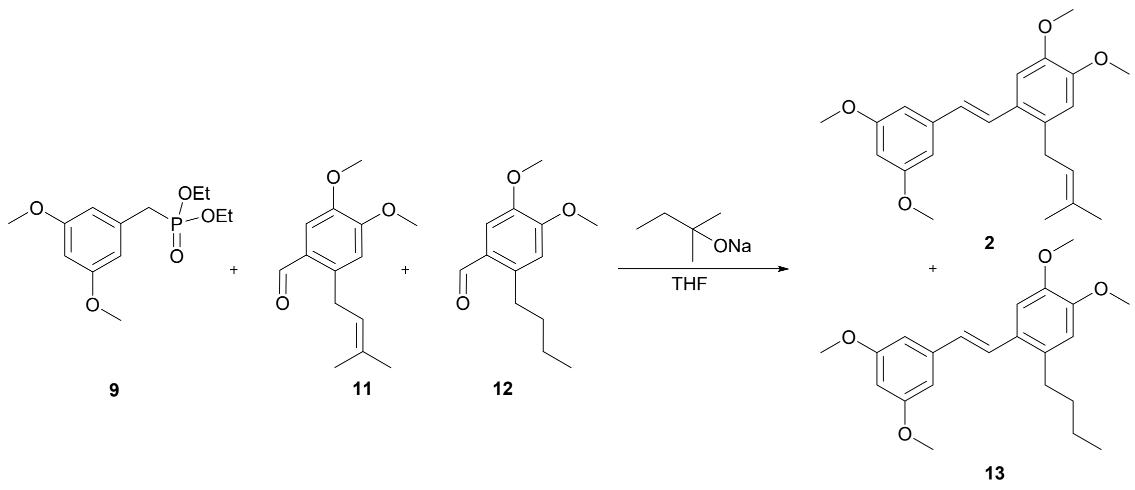

To the solution of 70.51 mg (0.6402 mmol) of sodium *tert*-pentoxide in dry THF (5 mL), the solution of 37.00 mg (0.1280 mmol) of diethyl 3,5-dimethoxybenzylphosphonate and 30.00 mg (0.1280 mmol) of mixture of 4,5-dimethoxy-2-(3-methylbut-2-en-1-yl) benzaldehyde and 2-butyl-4,5-dimethoxy benzaldehyde in dry THF (1 mL) was dropwise added. The reaction mixture was stirred at reflux under an inert atmosphere. The reaction was monitored by thin-layer chromatography (ethyl acetate/*n*-hexane, 1/4). To the reaction mixture was water (5 mL) added and extracted three times into ethyl acetate (3×10 mL). The organic layer was washed with brine (10 mL) and dried over anhydrous $MgSO_4$. The solvent was evaporated under reduced pressure. The product was purified by column chromatography (ethyl acetate/*n*-hexane, 1/4) to produce 9.00 mg (19 %) of mixture of 51 % of 1-[(*E*)-2-(3,5-dimethoxyphenyl)ethenyl]-4,5-dimethoxy-2-(3-methylbut-2-en-1-yl)benzene and 49 % of 1-butyl-2-[(*E*)-2-(3,5-dimethoxyphenyl)ethenyl]-4,5-dimethoxybenzene as yellow oil. IR (ATR) ν_{max}/cm^{-1} 2928, 2850, 1590, 1512, 1458, 1356, 1272, 1198, 1150, 1104, 957, 846. ^1H NMR (400 MHz, $CDCl_3$) δ 7.31 (d, $^3J_{HH}$ = 16.0 Hz, 1H), 7.30 (d, $^3J_{HH}$ = 16.0 Hz, 1H), 7.12–7.11 (m, 2H), 6.84 (d, $^3J_{HH}$ = 16.0 Hz, 1H), 6.84 (d, $^3J_{HH}$ = 16.0 Hz, 1H), 6.71–6.65 (m, 6H), 6.40 (q, $^4J_{HH}$ = 2.3 Hz, 2H), 5.30–5.20 (m, 1H), 3.95 (s, 6H), 3.92–3.89 (m, 6H), 3.87–3.82 (m, 12H), 3.42 (d, $^3J_{HH}$ = 6.9 Hz, 2H), 2.74–2.67 (m, 2H), 1.79 (s, 3H), 1.75 (s, 3H), 1.60–1.55 (m, 2H), 1.46–1.37 (m, 2H), 0.96 (t, $^3J_{HH}$ = 7.3 Hz, 4H). ^{13}C NMR (101 MHz, $CDCl_3$) δ 161.0, 148.9, 148.8, 147.4, 147.3, 140.0, 133.9, 132.6, 132.0, 128.0, 127.9, 127.7, 127.6, 126.70, 126.65, 123.4, 112.6, 112.5, 108.5, 108.4, 104.44, 104.41, 99.5, 99.4, 56.0, 55.9, 55.0, 33.9, 32.8, 32.0, 25.7, 22.6, 18.1, 14.0. HRMS *m/z* calcd for $C_{23}H_{29}O_4$: 369.2060; found: $[M + H]^+$ 369.2051.

5-[(*E*)-2-(3,4-dimethoxyphenyl)ethenyl]-1,3-dimethoxy-2-(3-methylbut-2-en-1-yl)benzene 4

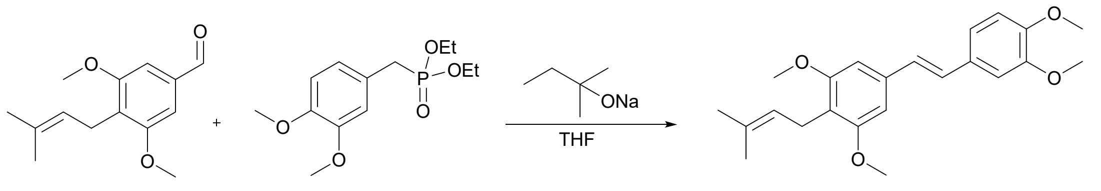

16 **20** **4**

To the solution of 235.00 mg (2.1342 mmol) of sodium *tert*-pentoxide in dry THF (5 mL), the solution of 123.50 mg (0.4268 mmol) of diethyl 3,4-dimethoxybenzylphosphonate and 100.00 mg (0.4268 mmol) of 4-(3-methylbut-2-en-1-yl)-3,5-dimethoxybenzaldehyde in dry THF (1 mL) was dropwise added. The reaction mixture was stirred at reflux under an inert atmosphere. The reaction was monitored by thin-layer chromatography (ethyl acetate/*n*-hexane, 1/4). To the reaction mixture was water (5 mL) added and extracted three times into ethyl acetate (3×10 mL). The organic layer was washed with brine (10 mL) and dried over anhydrous $MgSO_4$. The solvent was evaporated under reduced pressure. The product was purified by column chromatography (ethyl acetate/*n*-hexane, 1/4) to produce 78.00 mg (50 %) of 5-[(*E*)-2-(3,4-dimethoxyphenyl)ethenyl]-1,3-dimethoxy-2-(3-methylbut-2-en-1-yl)benzene as yellow oil. IR (ATR) ν_{max}/cm^{-1} 2905, 2827, 1590, 1500, 1456, 1425, 1344, 1251, 1236, 1203, 1150, 1085, 1066, 1032, 955, 888, 842, 762*,* 718. ^{1}H NMR (400 MHz, $CDCl_3$) δ 7.10–7.05 (m, 2H), 7.02 (d, $^3J_{HH}$ = 16.2 Hz, 1H), 6.95 (d, $^3J_{HH}$ = 16.2 Hz, 1H), 6.87 (d, $^3J_{HH}$ = 8.1 Hz, 1H), 6.72–6.68 (m, 2H), 5.24–5.17 (m, 1H), 3.96 (s, 3H), 3.92 (s, 3H), 3.88 (s, 6H), 3.36 (d, $^3J_{HH}$ = 7.2 Hz, 2H), 1.79 (s, 3H), 1.68 (s, 3H). ^{13}C NMR (101 MHz, $CDCl_3$) δ 158.1, 149.1, 148.8, 136.3, 131.2, 130.5, 127.7, 127.3, 122.7, 119.8, 118.0, 111.2, 108.6, 102.1, 55.9, 55.82, 55.76, 25.8, 22.3, 17.7. HRMS *m/z* calcd for $C_{23}H_{29}O_4$: 369.2060; found: $[M + H]^+$ 369.2052.

Demethylation

Demethylation of 1,3-dimethoxy-5-[(*E*)-2-[4-methoxy-3-(3-methylbut-2-en-1-yl)phenyl]-ethenyl]benzene 1

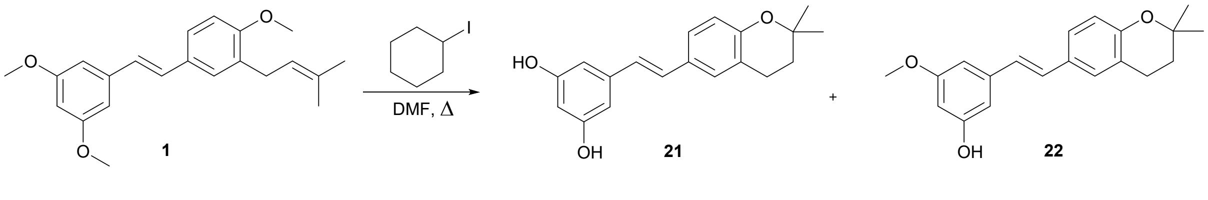

1 **21** **22**

0.50 g (0.0015 mol) of 1,3-Dimethoxy-5-[(*E*)-2-[4-methoxy-3-(3-methylbut-2-en-1-yl)phenyl]ethenyl]benzene was dissolved in anhydrous DMF (10 mL) and 4.50 g (0.0222 mol) of iodocyclohexane was added. The reaction mixture was heated under reflux under an atmosphere of inert gas overnight. After the finished reaction, the mixture was cooled down, poured into the water (10 mL), extracted with ethyl acetate (3×10 mL). The organic layer was washed with saturated aqueous solution of $NaHSO_3$ (10 mL), brine (10 mL) and dried over anhydrous Na_2SO_4. The solvent was evaporated under reduced pressure and purified by column chromatography (ethyl acetate/*n*-hexane, 2/1) to produce 0.37 g of a mixture of products. This mixture of products was dissolved in MeOH and separated by semipreparative HPLC. The mobile phase was removed under reduced pressure, followed by extraction of water residue to

chloroform. The chloroform was removed under reduced pressure and finally under a stream of nitrogen, and yellowish precipitates were obtained. We tried to avoid exposing the isolated compounds to sunlight in order to prevent *cis/trans* isomerization.

5-[(*E*)-2-(2,2-dimethyl-3,4-dihydro-*2H*-1-benzopyran-6-yl)ethenyl]benzene-1,3-diol 21

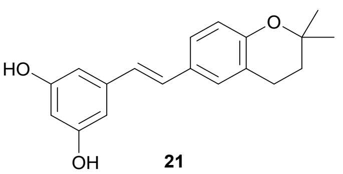

21

Isolated yield: 6 % (yellow oil). IR (ATR) ν_{max}/cm^{-1} 3388, 2969, 2926, 2860, 1591, 1496, 1454, 1342, 1259, 1150, 951, 827, 755. ^{1}H NMR (400 MHz, $CDCl_3$) δ 7.26–7.18 (m, 2H), 6.97 (d, $^{3}J_{HH}$ = 16.1 Hz, 1H), 6.85–6.74 (m, 2H), 6.55 (d, $^{4}J_{HH}$ = 1.9 Hz, 2H), 6.30–6.20 (m, 1H), 4.78 (br. s., 2H), 2.81 (t, $^{3}J_{HH}$ = 6.7 Hz, 2H), 1.83 (t, $^{3}J_{HH}$ = 6.7 Hz, 2H), 1.40–1.32 (s, 6H). ^{13}C NMR (101 MHz, $CDCl_3$) δ 156.8, 154.1, 140.6, 129.5, 128.7, 127.9, 125.7, 125.2, 121.0, 117.6, 105.8, 101.7, 74.6, 32.8, 26.9, 22.5. HRMS *m/z* calcd for $C_{19}H_{19}O_3$: 295.1340; found: $[M - H]^-$ 295.1336.

3-[(E)-2-(2,2-dimethyl-3,4-dihydro-2H-1-benzopyran-6-yl)ethenyl]-5-methoxyphenol 22

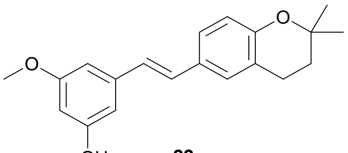

22

Isolated yield: 4 % (yellow oil). IR (ATR) ν_{max}/cm^{-1} 3388, 2924, 2847, 1588, 1503, 1454, 1419, 1251, 1156, 1111, 952, 825, 617. ^{1}H NMR (400 MHz, $CDCl_3$) δ 7.27–7.20 (m, 2 H), 6.99 (d, $^{3}J_{HH}$ = 16.1 Hz, 1 H), 6.85 (d, $^{3}J_{HH}$ = 16.1 Hz, 1 H), 6.78 (d, $^{3}J_{HH}$ = 8.5 Hz, 1 H), 6.62–6.58 (m, 2 H), 6.33–6.30 (m, 1 H), 4.83 (br. s., 1 H), 3.82 (s, 3 H), 2.81 (t, $^{3}J_{HH}$ = 6.7 Hz, 2 H), 1.83 (t, $^{3}J_{HH}$ = 6.7 Hz, 2 H), 1.36 (s, 6 H). ^{13}C NMR (101 MHz, $CDCl_3$) δ 161.1, 156.7, 154.1, 140.2, 129.3, 128.8, 127.9, 125.7, 125.6, 121.0, 117.6, 105.6, 104.6, 100.4, 74.6, 55.3, 32.8, 26.9, 22.5. HRMS *m/z* calcd for $C_{20}H_{21}O_3$: 309.1496; found: $[M - H]^-$ 309.1496.

Demethylation of 1,3-dimethoxy-5-[(*E*)-2-(4-methoxyphenyl)ethenyl]-2-(3-methylbut-2-en-1-yl)benzene 3

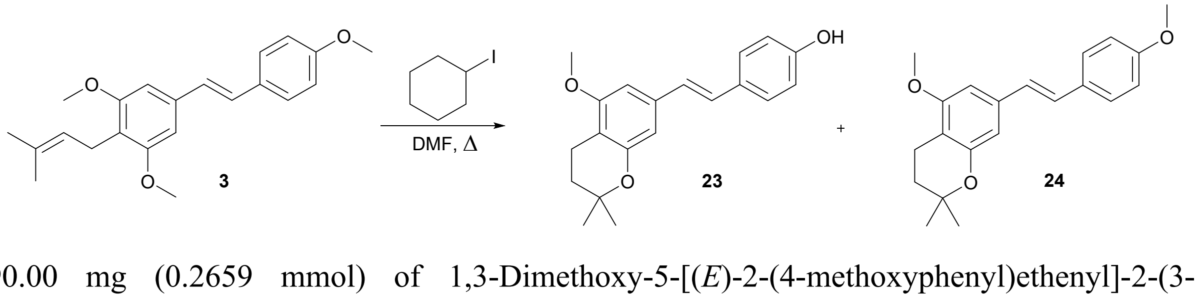

90.00 mg (0.2659 mmol) of 1,3-Dimethoxy-5-[(*E*)-2-(4-methoxyphenyl)ethenyl]-2-(3-methylbut-2-en-1-yl)benzene was dissolved in anhydrous DMF (3 mL) and 0.84 g (3.9889 mol) of iodocyclohexane was added. The reaction mixture was heated under reflux under an atmosphere of inert gas overnight. After the finished reaction, the mixture was cooled down,

poured into the water (5 mL), extracted with ethyl acetate (3×5 mL). The organic layer was washed with saturated aqueous solution of $NaHSO_3$ (5 mL), brine (5 mL) and dried over anhydrous Na_2SO_4. The solvent was evaporated under reduced pressure and purified by column chromatography (ethyl acetate/*n*-hexane, 2/1) to produce 50.25 mg of a mixture of products. This mixture of products was dissolved in MeOH and separated by semipreparative HPLC. The mobile phase was removed under reduced pressure, followed by extraction of water residue to chloroform. The chloroform was removed under reduced pressure and finally under a stream of nitrogen, and yellowish precipitates were obtained. We tried to avoid exposing the isolated compounds to sunlight in order to prevent *cis/trans* isomerization.

4-[(*E*)-2-(5-Methoxy-2,2-dimethyl-3,4-dihydro-2H-1-benzopyran-7-yl)ethenyl]phenol 23*

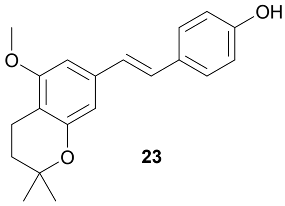

23

Isolated yield: 7 % (yellow oil). IR (ATR) ν_{max}/cm^{-1} 3391, 2928, 1596, 1457, 1428, 1350, 1291, 1201, 1149, 1058, 1004, 822, 698. ^1H NMR (400 MHz, $CDCl_3$) δ 7.39 (d, J = 8.5 Hz, 2 H), 7.00 (d, $^3J_{HH}$ = 16.2 Hz, 1 H), 6.87 (d, $^3J_{HH}$ = 16.2 Hz, 1 H), 6.83 (d, J = 8.5 Hz, 2 H), 6.62 (s, 1 H), 6.54 (s, 1 H), 4.81 (bs, 1 H), 3.88 (s, 3 H), 2.65 (t, J = 6.8 Hz, 2 H), 1.79 (t, J = 6.8 Hz, 2 H), 1.34 (s, 6 H). ^{13}C NMR (101 MHz, $CDCl_3$) δ 158.0, 155.1, 154.7, 136.7, 130.5, 127.8, 127.5, 127.0, 115.5, 109.6, 108.0, 99.5, 74.0, 55.4, 32.3, 26.7, 17.2. HRMS *m/z* calcd for $C_{20}H_{21}O_3$: 309.1496; found: $[M - H]^-$ 309.1502.

*The structure of the compound **23** was determined from the shift of the methoxy group in the *para* position of starting stilbenoid **3** which was at 3.84 ppm and in product **23**, it has disappeared. The signal on the second aromatic ring at 3.88 ppm of compound **3**, which was equal to 6 hydrogens (2 equal methoxy groups), remains in product **23** but only with 3 hydrogens, so one of the methoxy groups has been demethylated.

5-Methoxy-7-[(*E*)-2-(4-methoxyphenyl)ethenyl]-2,2-dimethyl-3,4-dihydro-2H-1-benzopyran 24

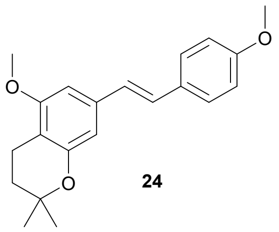

24

Isolated yield: 5 % (yellow oil). IR (ATR) ν_{max}/cm^{-1} 2942, 2887, 1595, 1501, 1381, 1258, 1162, 1093, 1024, 957, 883, 809, 589. ^1H NMR (400 MHz, $CDCl_3$) δ 7.44 (d, $^3J_{HH}$ = 8.7 Hz, 2 H), 7.02 (d, $^3J_{HH}$ = 16.2 Hz, 1 H), 6.93–6.85 (m, 3 H), 6.63 (s, 1 H), 6.55 (s, 1 H), 3.88 (s, 3 H), 3.84 (s, 3 H), 2.66 (t, $^3J_{HH}$ = 6.8 Hz, 2 H), 1.79 (t, $^3J_{HH}$ = 6.8 Hz, 2 H), 1.34 (s, 6 H). ^{13}C NMR (101 MHz, $CDCl_3$) δ 129.2, 158.0, 154.7, 136.7, 130.2, 127.63, 127.58, 126.9, 114.1, 109.5,

108.0, 99.5, 74.0, 55.3, 32.3, 26.7, 17.2. HRMS *m/z* calcd for $C_{21}H_{23}O_3$: 323.1653; found: $[M - H]^-$ 323.1662.

Demethylation of 5-[(*E*)-2-(3,4-dimethoxyphenyl)ethenyl]-1,3-dimethoxy-2-(3-methylbut-2-en-1-yl)benzene 4

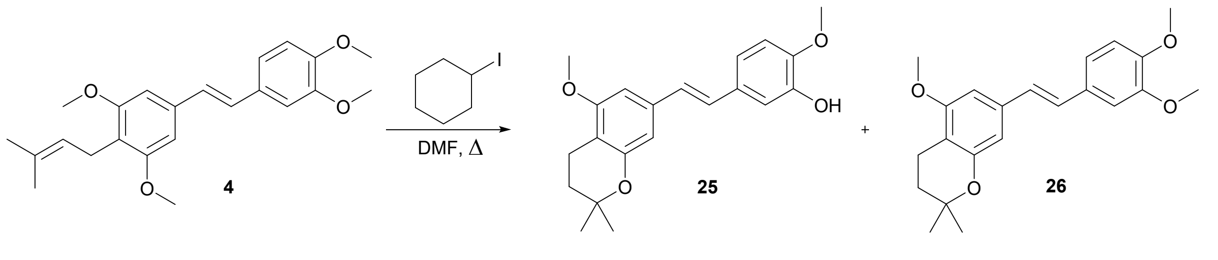

100.00 mg (0.2714 mmol) of 5-[(*E*)-2-(3,4-Dimethoxyphenyl)ethenyl]-1,3-dimethoxy-2-(3-methylbut-2-en-1-yl)benzene was dissolved in anhydrous DMF (3 mL) and 0.86 g (4.0709 mol) of iodocyclohexane was added. The reaction mixture was heated under reflux under an atmosphere of inert gas overnight. After the finished reaction, the mixture was cooled down, poured into the water (5 mL), extracted with ethyl acetate (3×5 mL). The organic layer was washed with saturated aqueous solution of $NaHSO_3$ (5 mL), brine (5 mL) and dried over anhydrous Na_2SO_4. The solvent was evaporated under reduced pressure and purified by column chromatography (ethyl acetate/*n*-hexane, 2/1) to produce 50.25 mg of a mixture of products. This mixture of products was dissolved in MeOH and separated by semipreparative HPLC. The mobile phase was removed under reduced pressure, followed by extraction of water residue to chloroform. The chloroform was removed under reduced pressure and finally under a stream of nitrogen, and yellowish precipitates were obtained. We tried to avoid exposing the isolated compounds to sunlight in order to prevent *cis/trans* isomerization.

2-Methoxy-5-[(*E*)-2-(5-methoxy-2,2-dimethyl-3,4-dihydro-2H-1-benzopyran-7-yl)ethenyl]phenol 25*

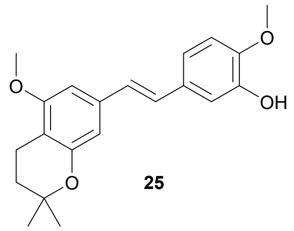

Isolated yield: 6 % (yellow oil). IR (ATR) ν_{max}/cm^{-1} 3244, 2932, 2839, 1588, 1455, 1413, 1236, 1118, 1026, 956, 818, 627. ^1H NMR (400 MHz, $CDCl_3$) δ 7.13 (d, $^3J_{HH}$ = 1.9 Hz, 1 H), 7.00–6.92 (m, 2 H), 6.90–6.81 (m, 2 H), 6.62 (s, 1 H), 6.54 (s, 1 H), 5.60 (s, 1 H), 3.92 (s, 3 H), 3.88 (s, 3 H), 2.65 (t, $^3J_{HH}$ = 6.7 Hz, 2 H), 1.79 (t, $^3J_{HH}$ = 6.9 Hz, 2 H), 1.34 (s, 6 H). ^{13}C NMR (101 MHz, $CDCl_3$) δ 158.0, 154.7, 146.3, 136.6, 131.2, 127.6, 127.4, 119.2, 111.7, 110.6, 109.6, 108.1, 99.5, 74.0, 56.0, 55.4, 32.3, 26.7, 17.2. HRMS *m/z* calcd for $C_{21}H_{23}O_4$: 339.1602; found: $[M - H]^-$ 339.1608.

*The structure of the compound **25**, was determined from the signal at 3.96 ppm present in the starting stilbenoid **4**, which belongs to the meta methoxy group according to the NOESY experiment of a similar structure, which has disappeared in the compounds **25**. From this

observation, we can guess that the group in the meta position was deprotected. And the integral of a signal at 3.88 ppm was reduced to 3H compared to the starting compound **4**.

Furthermore, this statement can be supported from literature data[14] in which it was stated that the methoxy group in the *meta* position is visible in ^{1}H NMR at a higher shift than the one in the *para* position. Also, the signal at the higher shift has disappeared after demethylation. If we compare the NMR of the starting compound **4** and the double demethylated compound **25**, we can determine which methoxy group was demethylated.

7-[(*E*)-2-(3,4-Dimethoxyphenyl)ethenyl]-5-methoxy-2,2-dimethyl-3,4-dihydro-2H-1-benzopyran 26

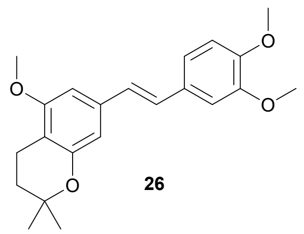

26

Isolated yield: 10 % (yellow oil). IR (ATR) ν_{max}/cm^{-1} 2935, 2852, 1586, 1458, 1382, 1321, 1110, 1032, 962, 826, 628. ^{1}H NMR (400 MHz, $CDCl_3$) δ 7.08–6.97 (m, 3 H), 6.92–6.83 (m, 2 H), 6.64 (s, 1 H), 6.55 (s, 1 H), 3.95 (s, 3 H), 3.91 (s, 3 H), 3.88 (s, 3 H), 2.66 (t, $^{3}J_{HH}$ = 6.7 Hz, 2 H), 1.79 (t, $^{3}J_{HH}$ = 6.8 Hz, 2 H), 1.34 (s, 6 H). ^{13}C NMR (101 MHz, $CDCl_3$) δ 158.0, 154.7, 149.1, 148.8, 136.6, 130.6, 127.8, 127.1, 119.8, 111.2, 109.6, 108.7, 108.0, 99.5, 74.0, 55.9, 55.8, 55.4, 32.3, 26.7, 17.2. HRMS *m/z* calcd for $C_{22}H_{27}O_4$: 355.1904; found: $[M + H]^+$ 355.1910.

References:

1. M. C. Davis and T. J. Groshens. *Tetrahedron Lett.,* 2012, **53**, 3521-3523.
2. H.-T. Song, W. Ding, Q.-Q. Zhou, J. Liu, L.-Q. Lu and W.-J. Xiao. *J. Org. Chem.,* 2016, **81**, 7250-7255.
3. J. S. Tan and M. A. Ciufolini, *Org. Lett.*, 2006, **8**, 4771-4774.
4. C. Lu, Z. Bingqing, M. Xiaoyu, Z. Zaiyong, W. Jian-rong and Z. Q. M. Xuefeng, *Chem.-Eur. J.,* 2019, **25**, 6584 - 6590.
5. S. B. Beil, I. Uecker, P. Franzmann, T. Müller and S. R. Waldwogel. *Org. Lett.,* 2018, **20**, 4107-4110.
6. Y. Takaya, K. Terashima, J. Ito, Y.-H. He, M. Tateoka, N. Yamaguchi and M. Niwa, *Tetrahedron,* 2005, **61**, 10285-10290.
7. W. Xu, J.-P. Zou and W. Zhang, *Tetrahedron Lett*., 2010, **51**, 2639-2643.
8. Ch. E. Anson, C. S. Creaser, A. V. Malkov, L. Mojovic and G. R. Stephenson, *J. Organomet. Chem.*, 2003, **668**, 101-122.
9. M. A. Rizzacasa and M. V. Sargent, *J. Chem. Soc., Perkin Trans. 1.,* 1991, **4**, 841-844.
10. T. Kim, K. H. Jeong, K. S. Kang, M. Nakata and J. Ham, *Eur. J. Org. Chem*., 2017, **2017**, 1704-1712.
11. A. Maiti, M. Cuendet, V. L. Croy, D. C. Endringer, J. M. Pezzuto and M. Cushman, *J. Med. Chem.,* 2007, **50**, 2799-2806.

12. V. Lelakova, J. Hosek, P. Bobal, H. Pizova, M. Gazdova, M. Malanik, K. Jakubczyk, O. Vesely, P. Landa, V. Temml, S. Daniela, V. Prachyawarakorn, G. Ren, F. Zpurny, M. Oravec and K. Smejkal, *J. Nat. Prod.*, 2019, **82**, 1839-1848.
13. M. Nerilson, J. I. A. Andrade, K. C. S. Lima, F. N. Dos Santos, A. Barison, K. S. Salome, T. Matsuura and C. V. Nunez, *Nat. Prod. Res.*, 2013, **27**, 425-432.
14. F. Villalba, A. C. Albéniz, *Adv. Synth. Catal.*, 2021, 363, 4795-4804.

3. Spectral characteristics

^1H and ^{13}C NMR spectra of compound **18**.

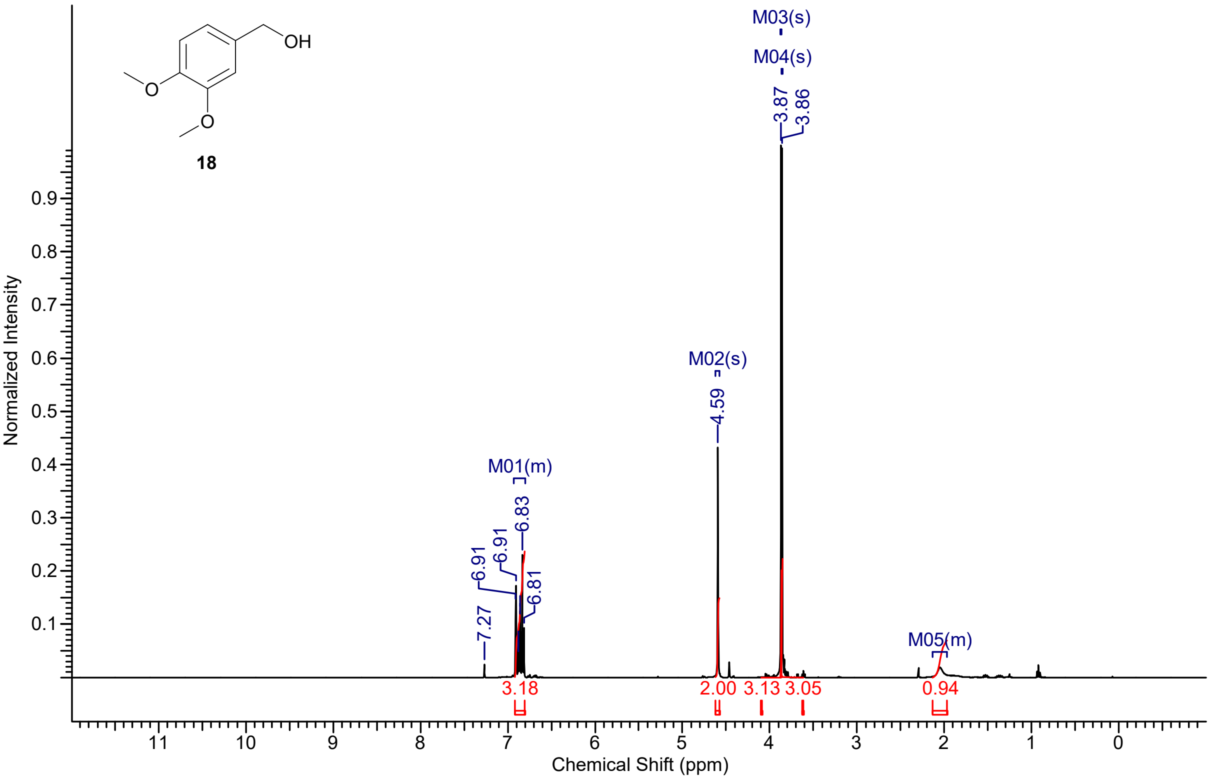

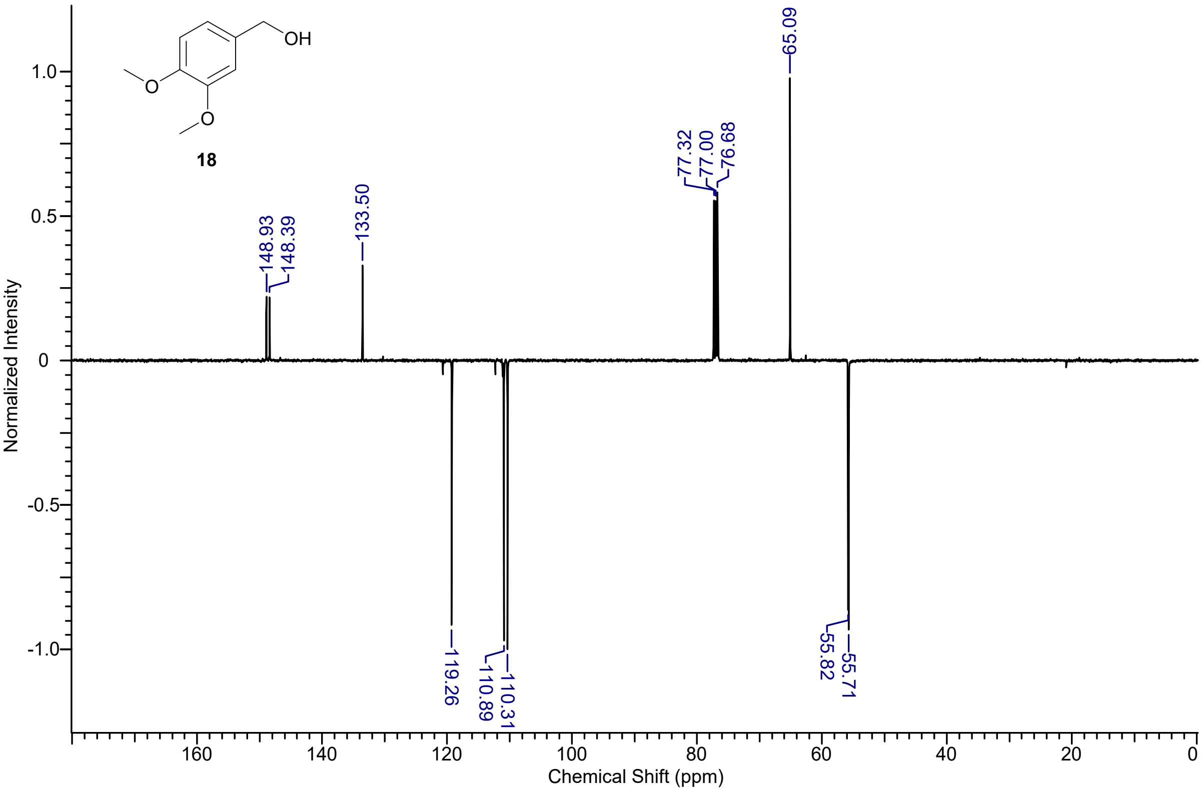

^{1}H and ^{13}C NMR spectra of compound **19**.

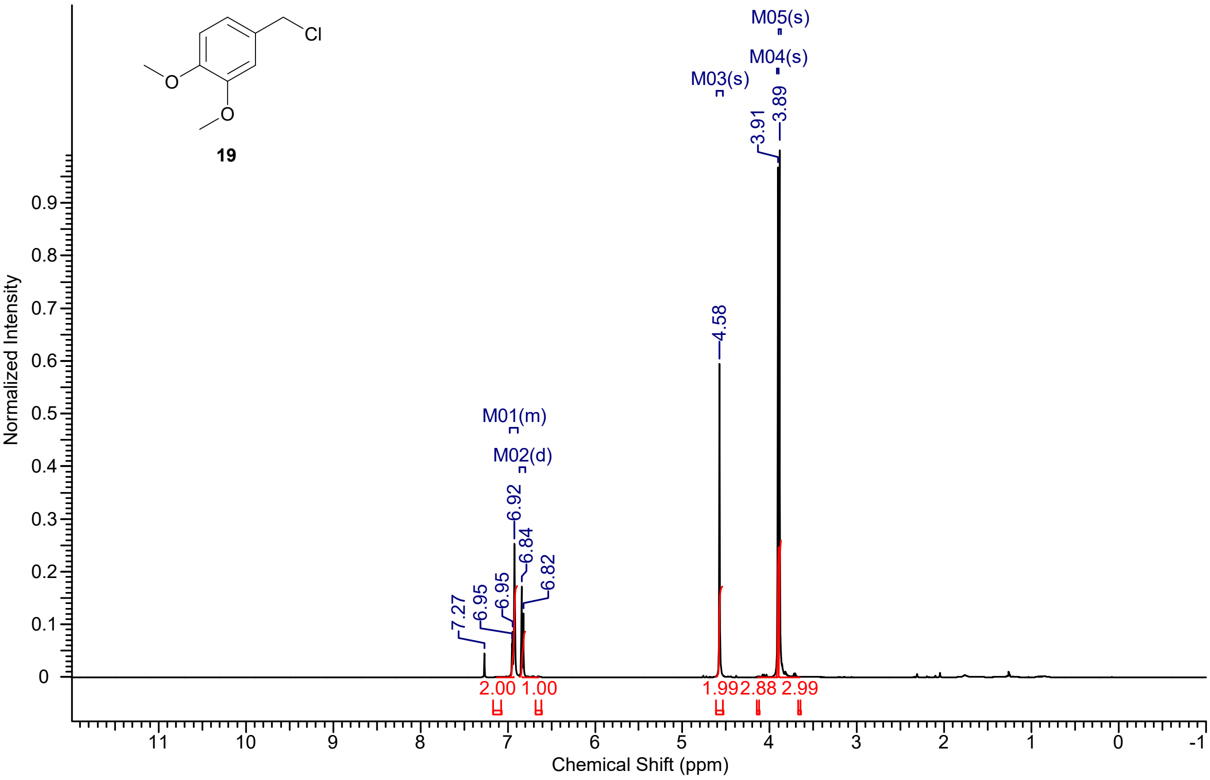

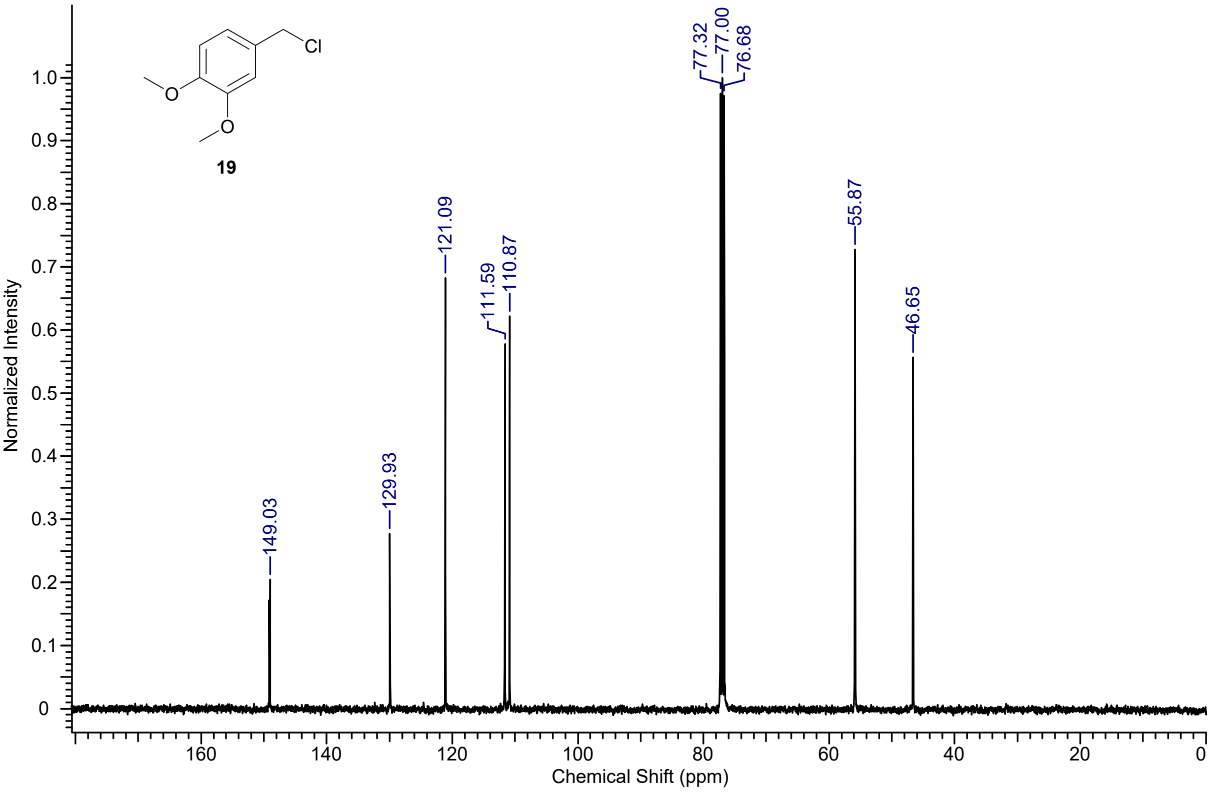

^{1}H and ^{13}C NMR spectra of compound **20**.

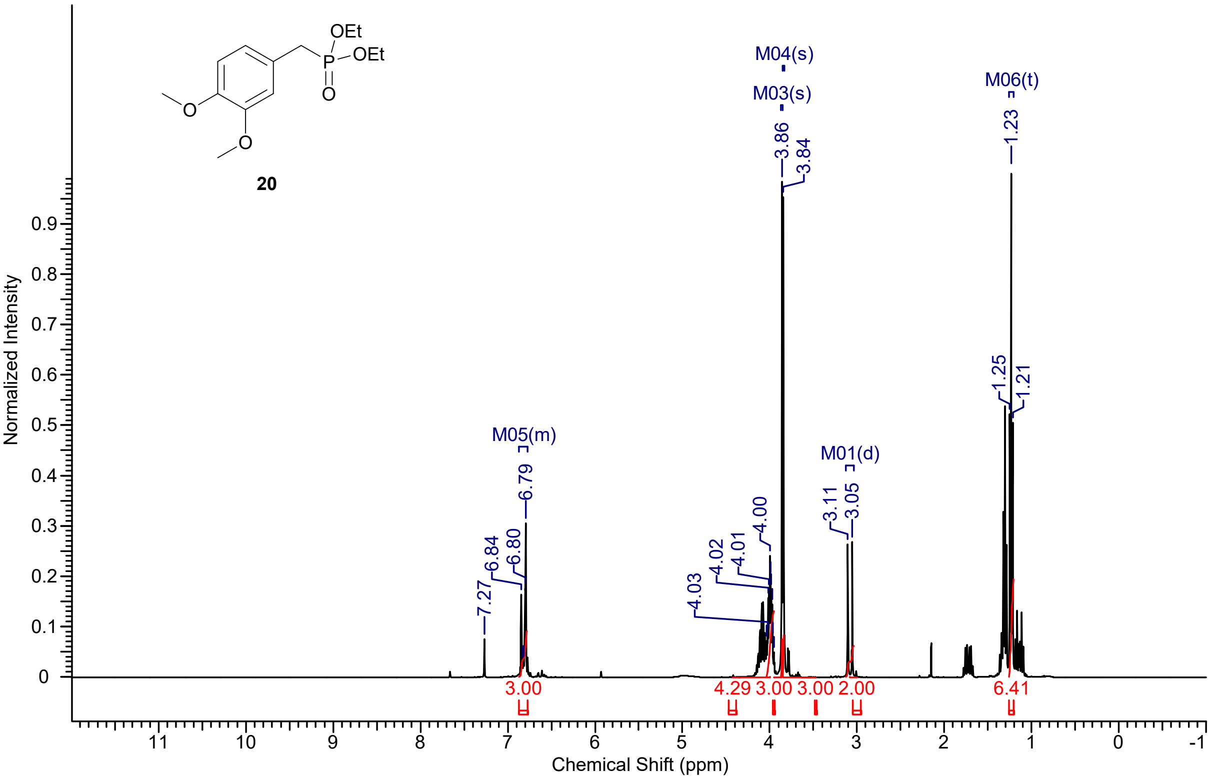

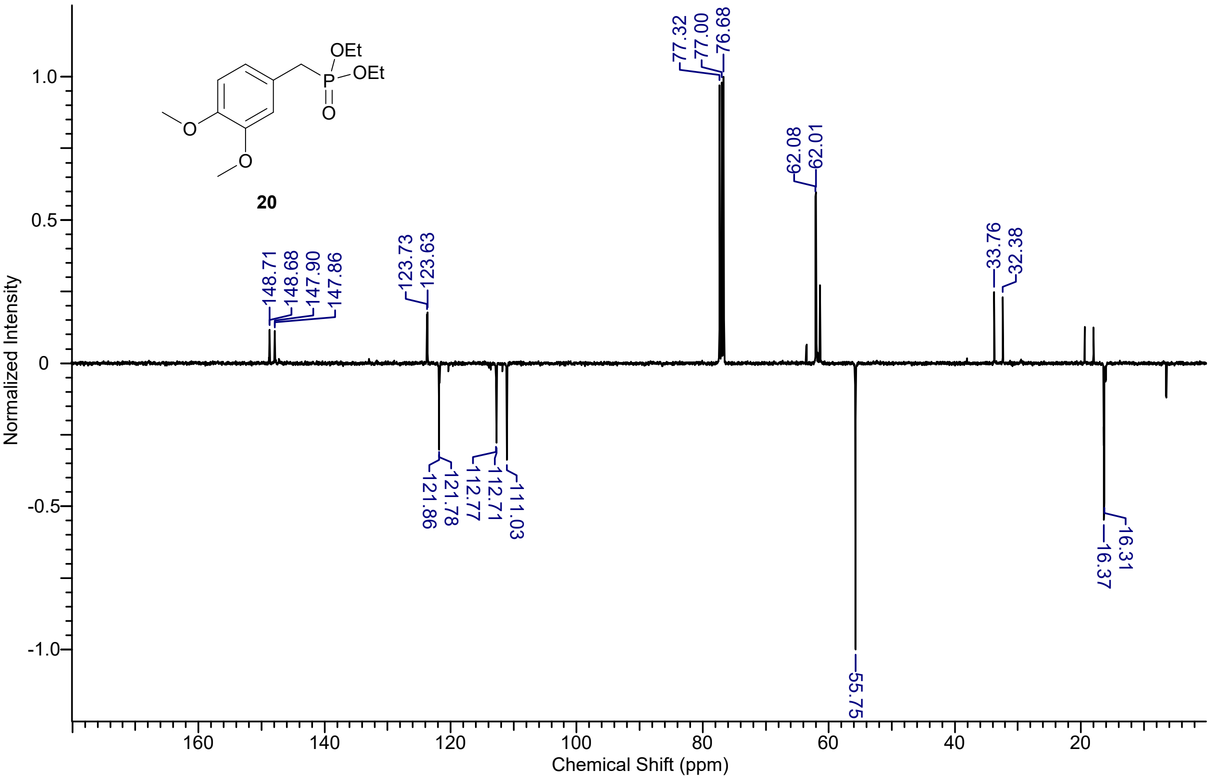

^{1}H and ^{13}C NMR spectra of compound **10**.

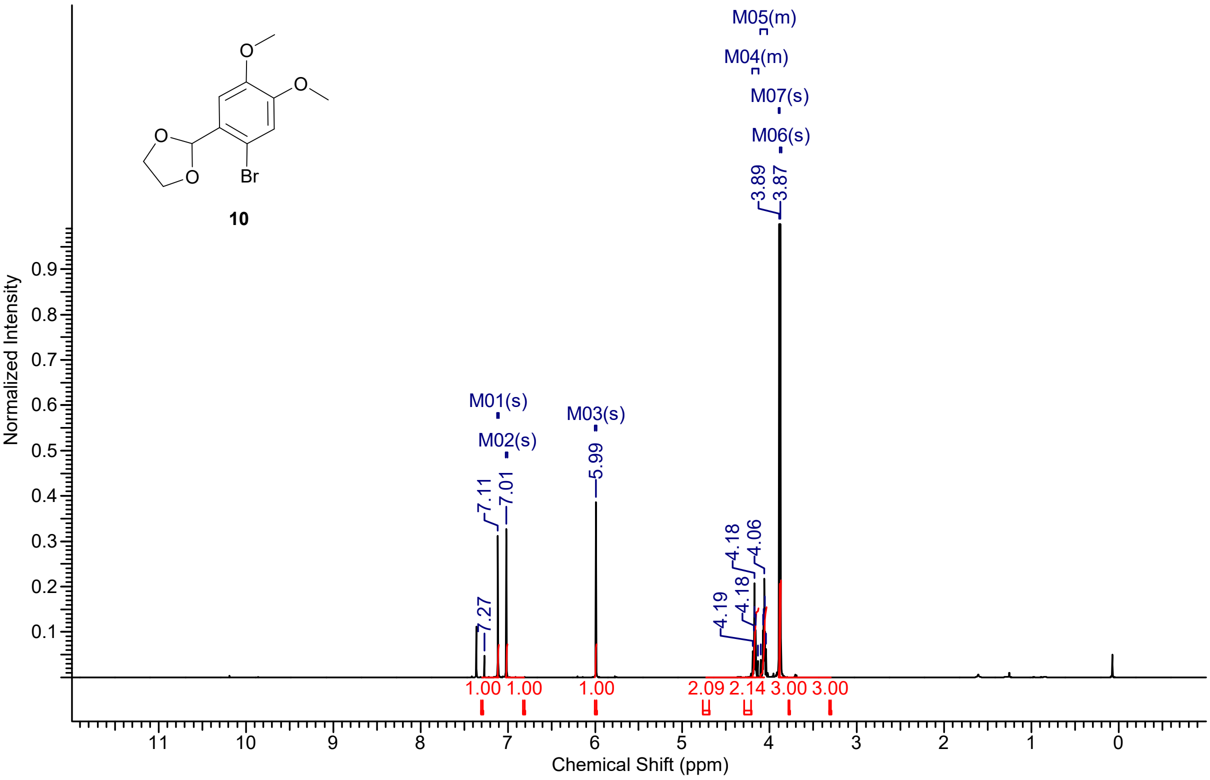

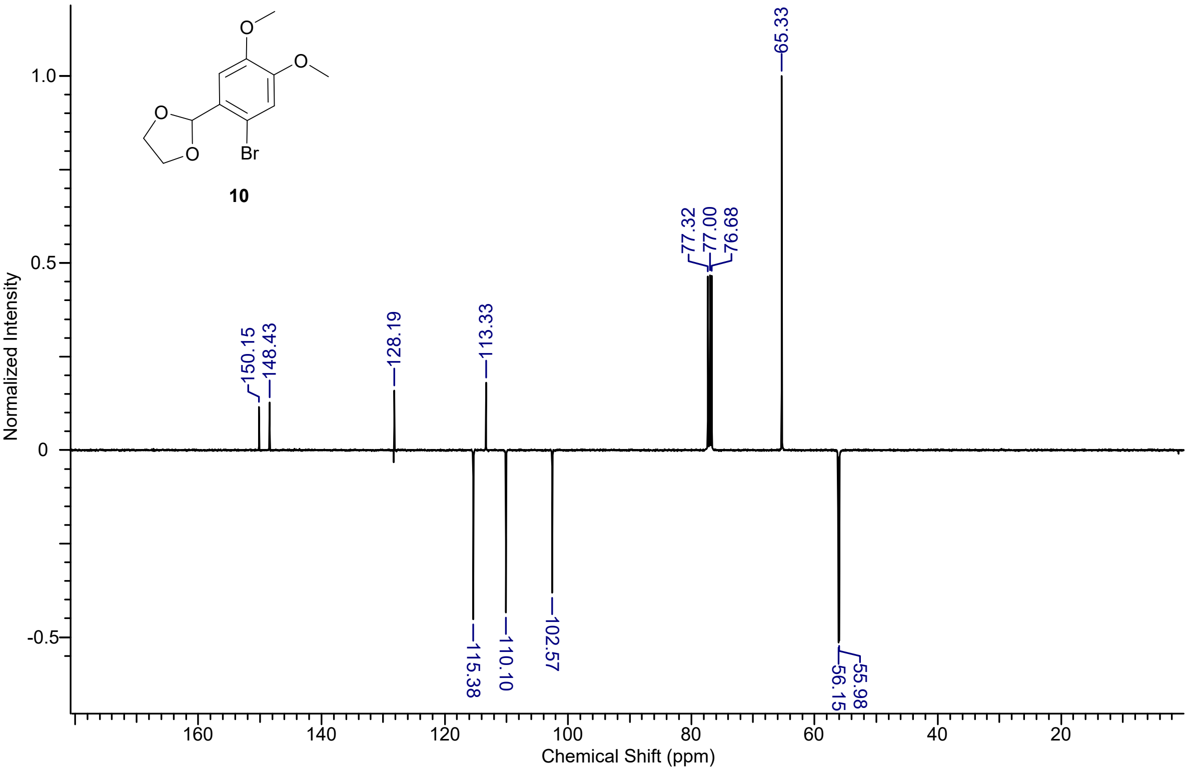

^{1}H and ^{13}C NMR spectra of compounds **11** and **12**.

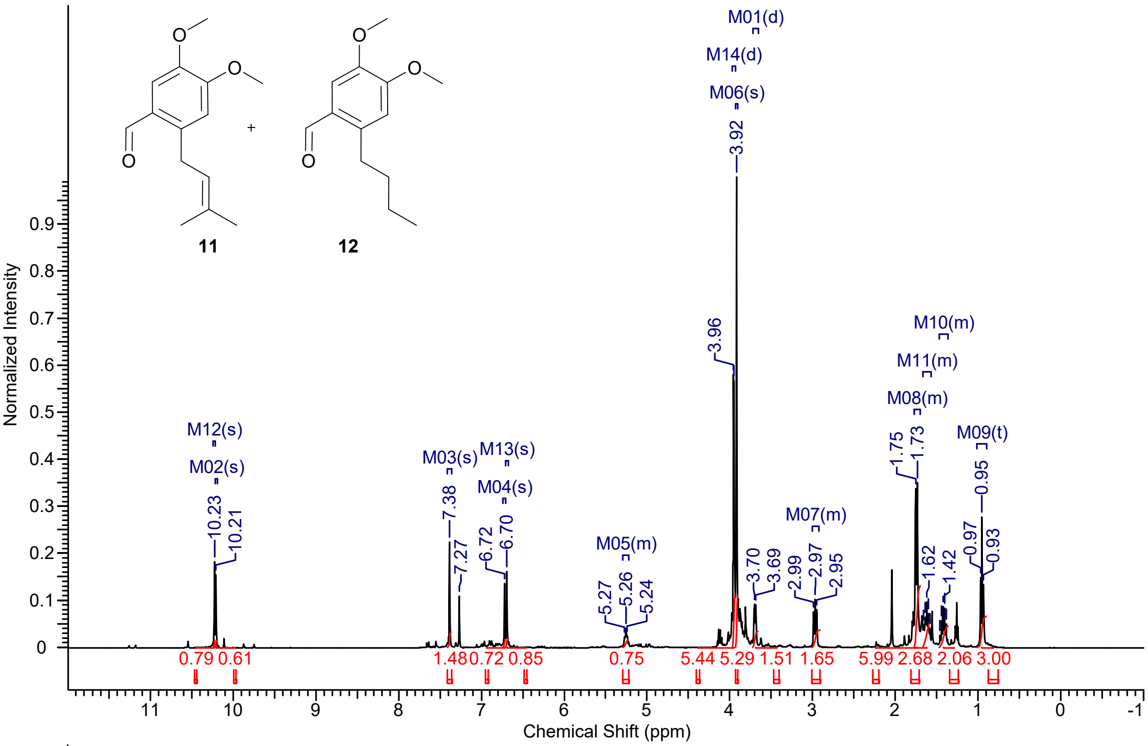

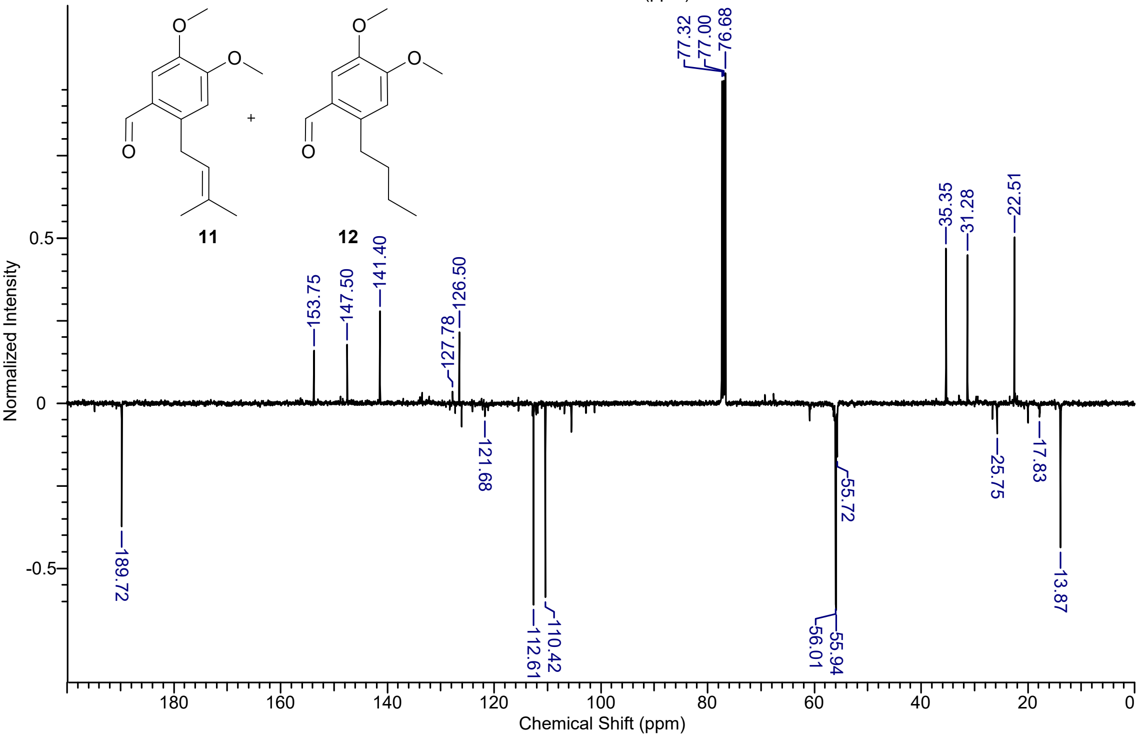

^{1}H and ^{13}C NMR spectra of (4-bromo-3,5-dimethoxyphenyl)methanol.

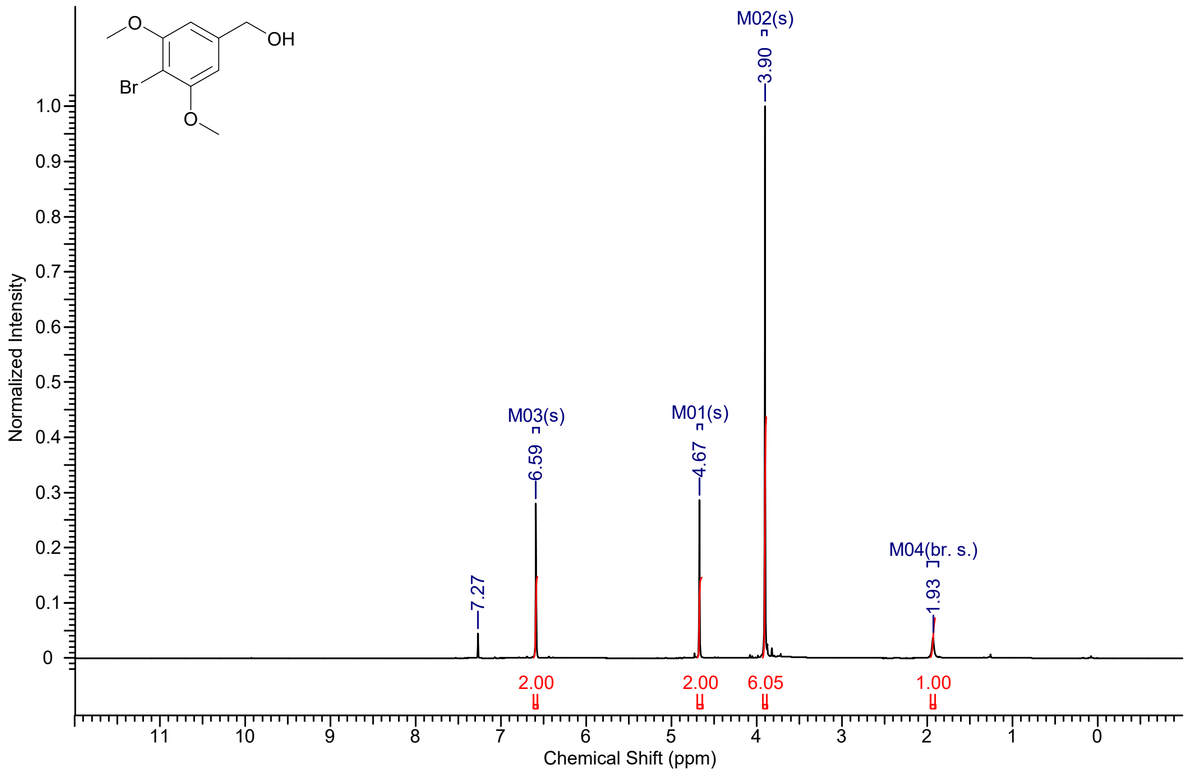

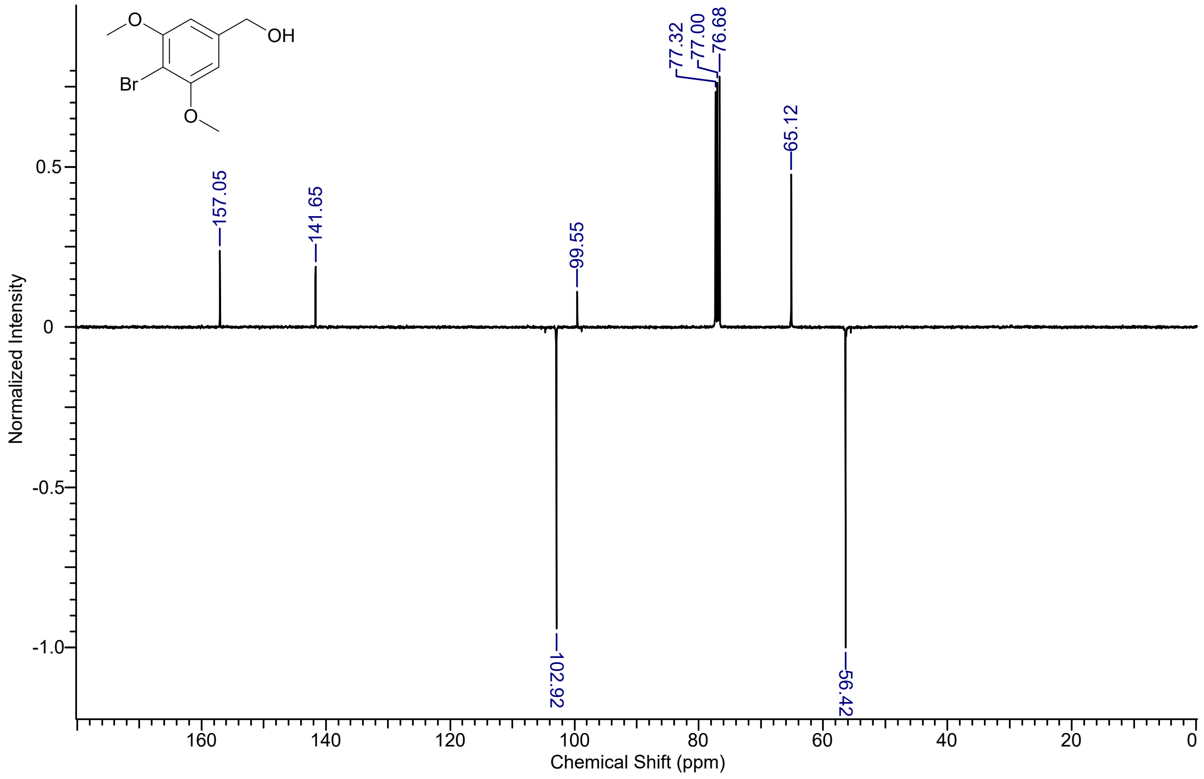

^{1}H and ^{13}C NMR spectra of compound **14**.

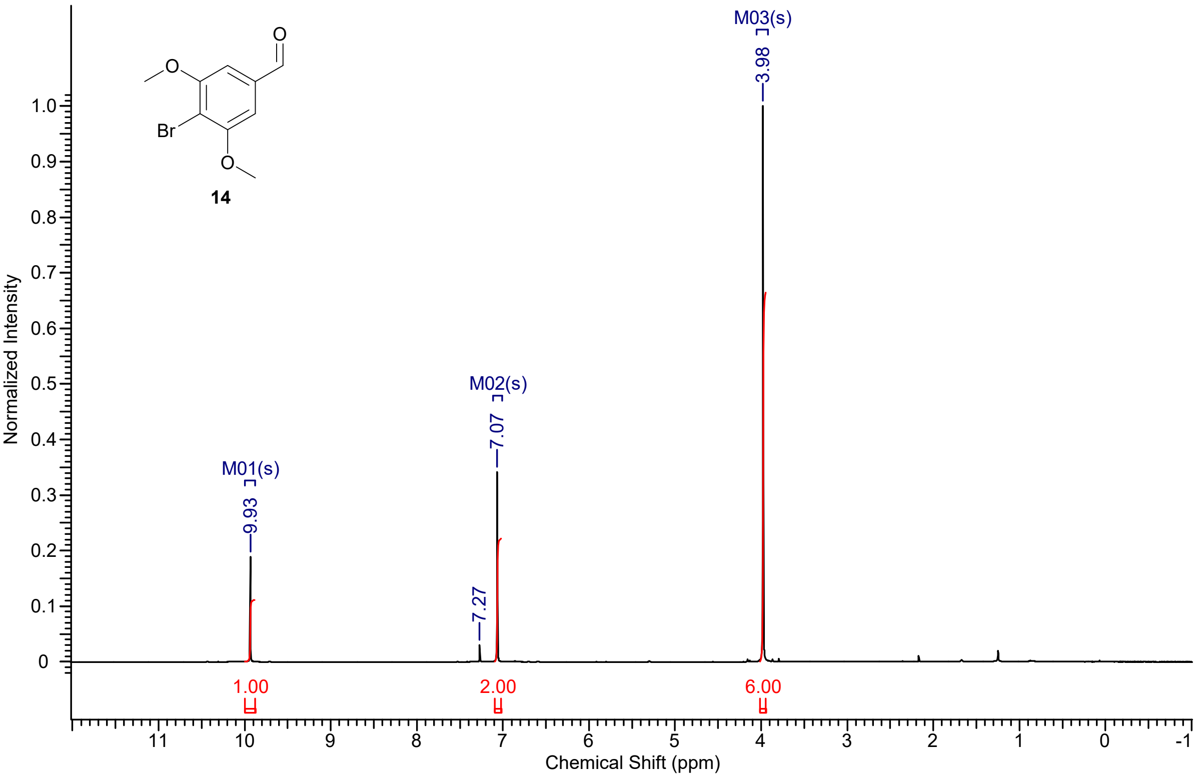

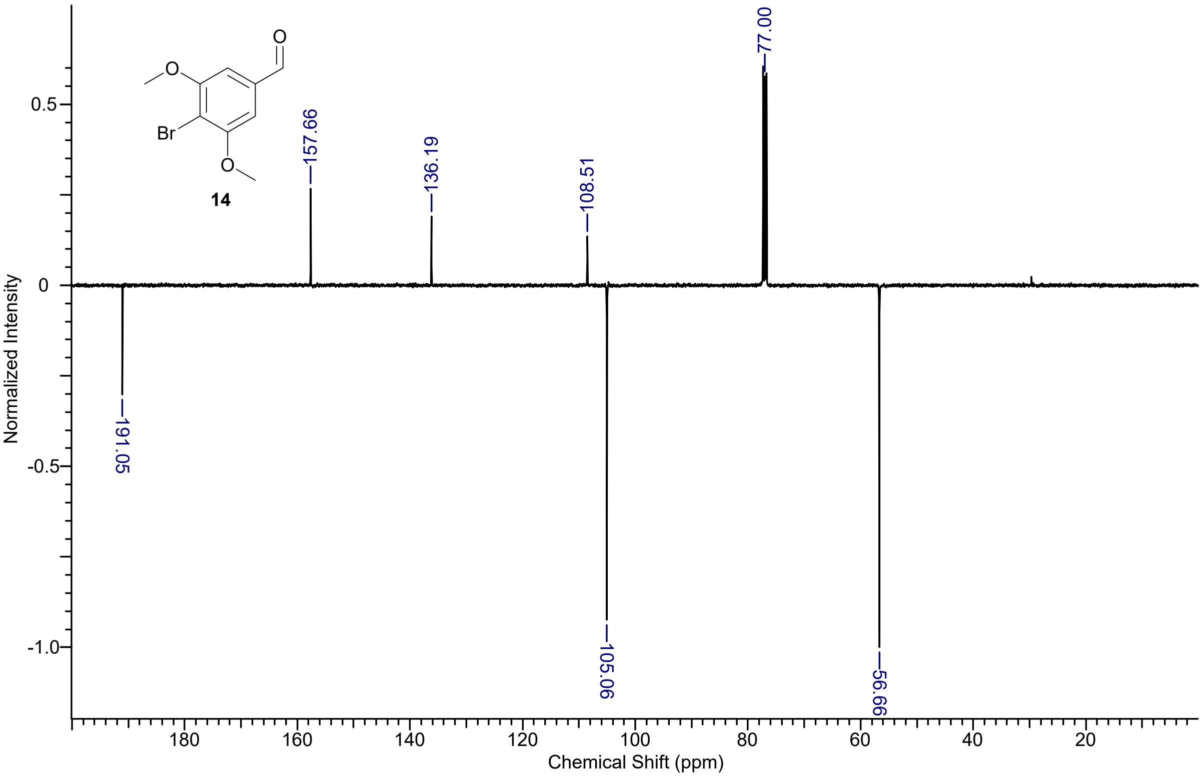

^{1}H and ^{13}C NMR spectra of compound **15**.

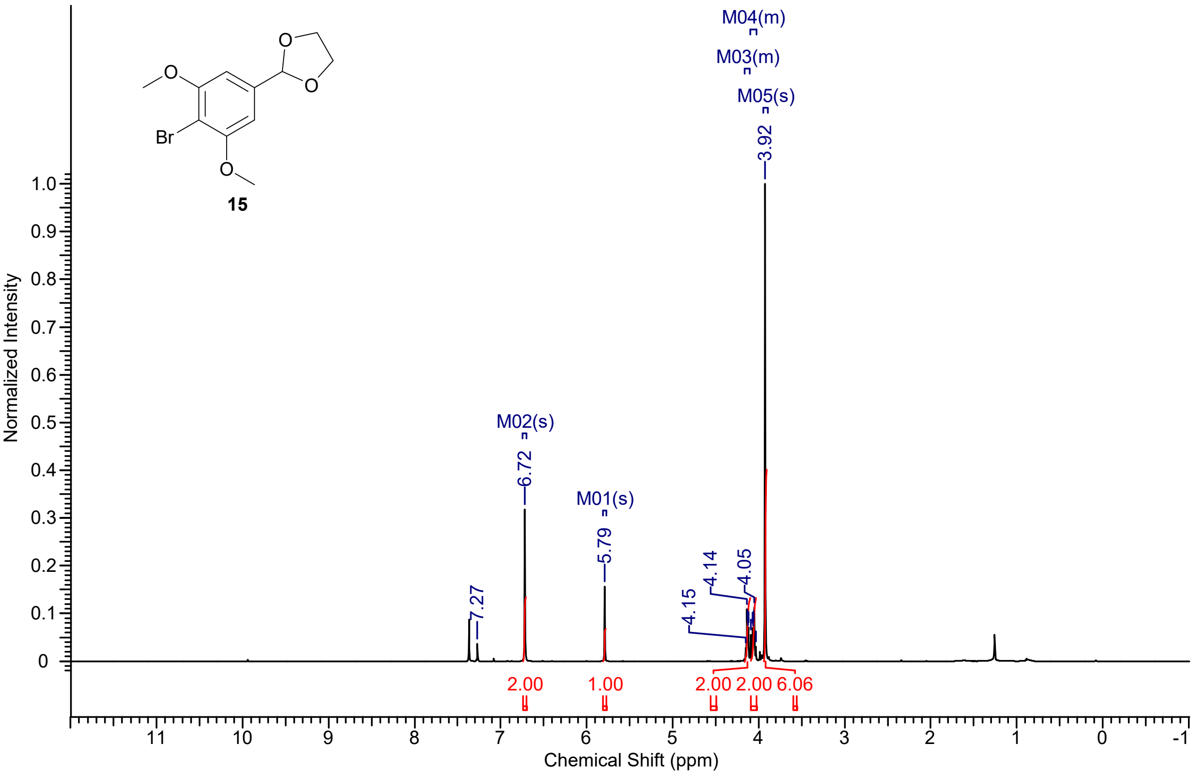

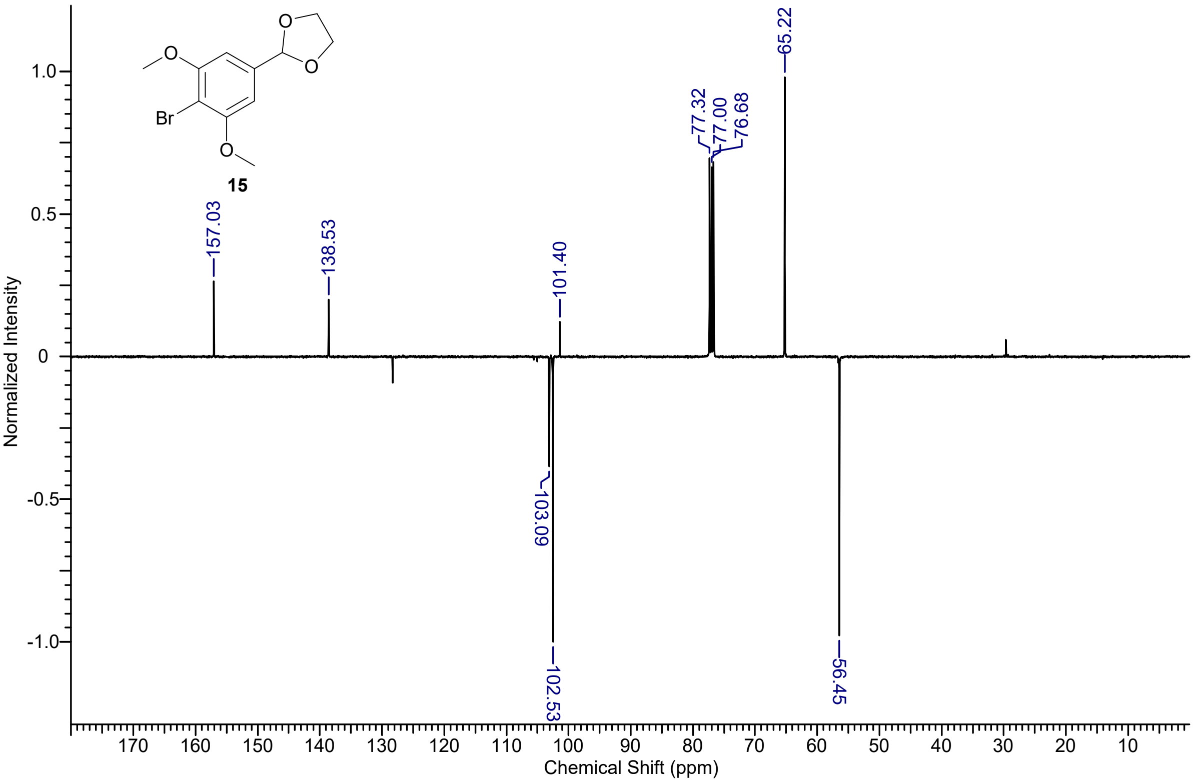

^{1}H, ^{13}C NMR and HRMS spectra of compound **16**.

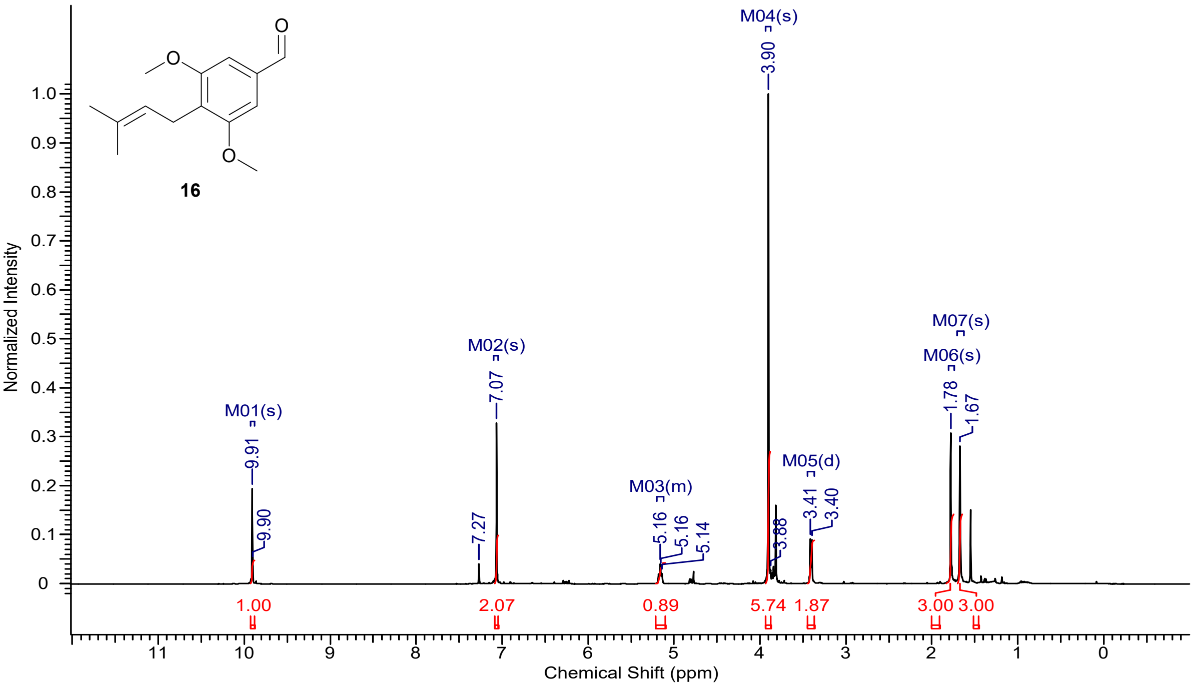

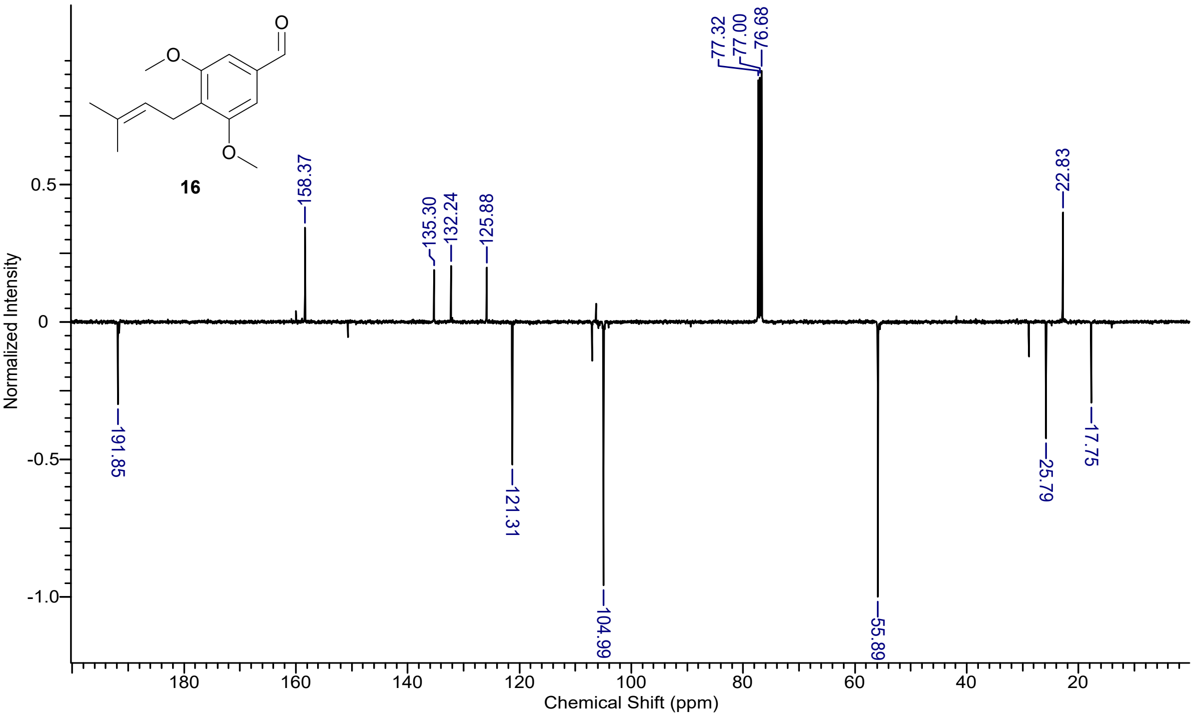

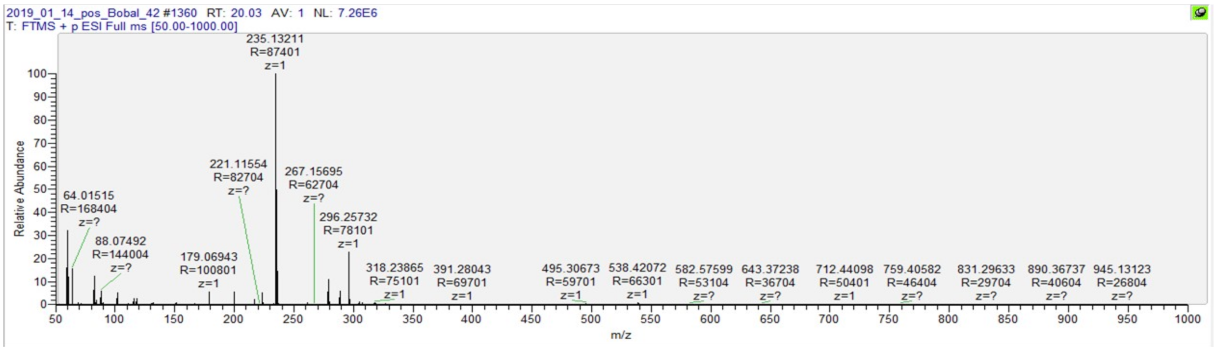

^{1}H and ^{13}C NMR spectra of compound **5**.

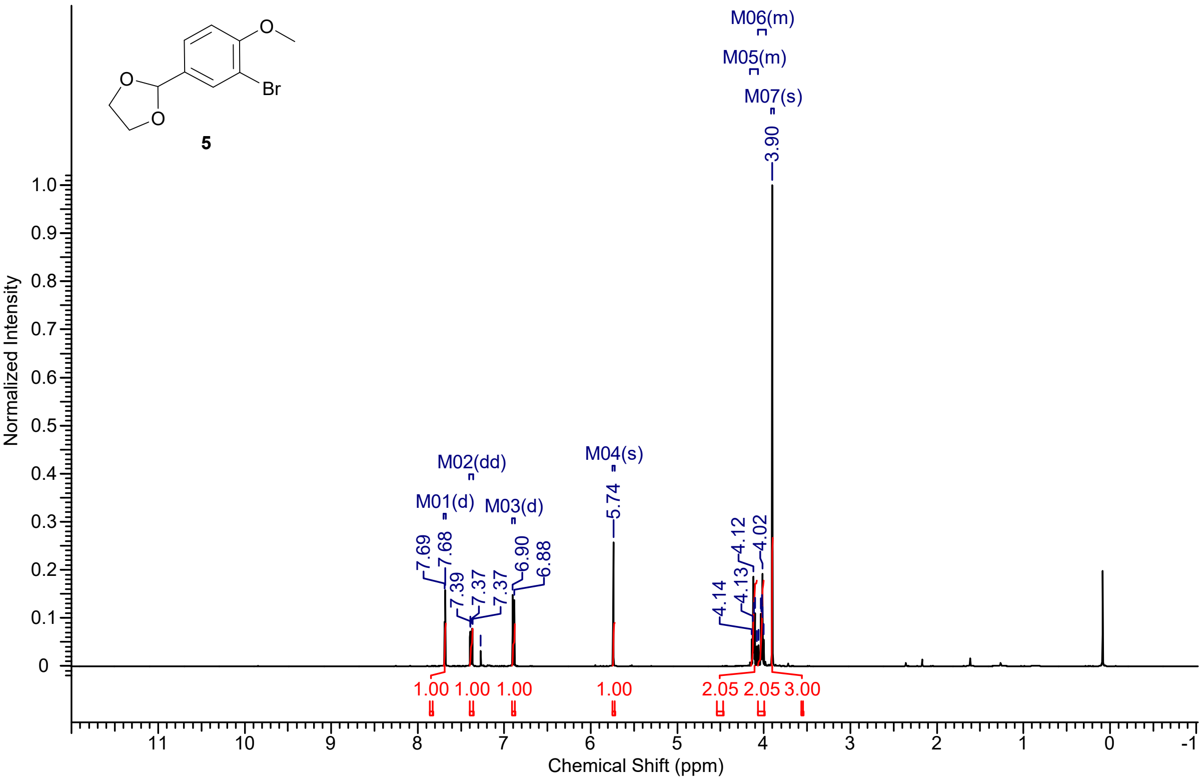

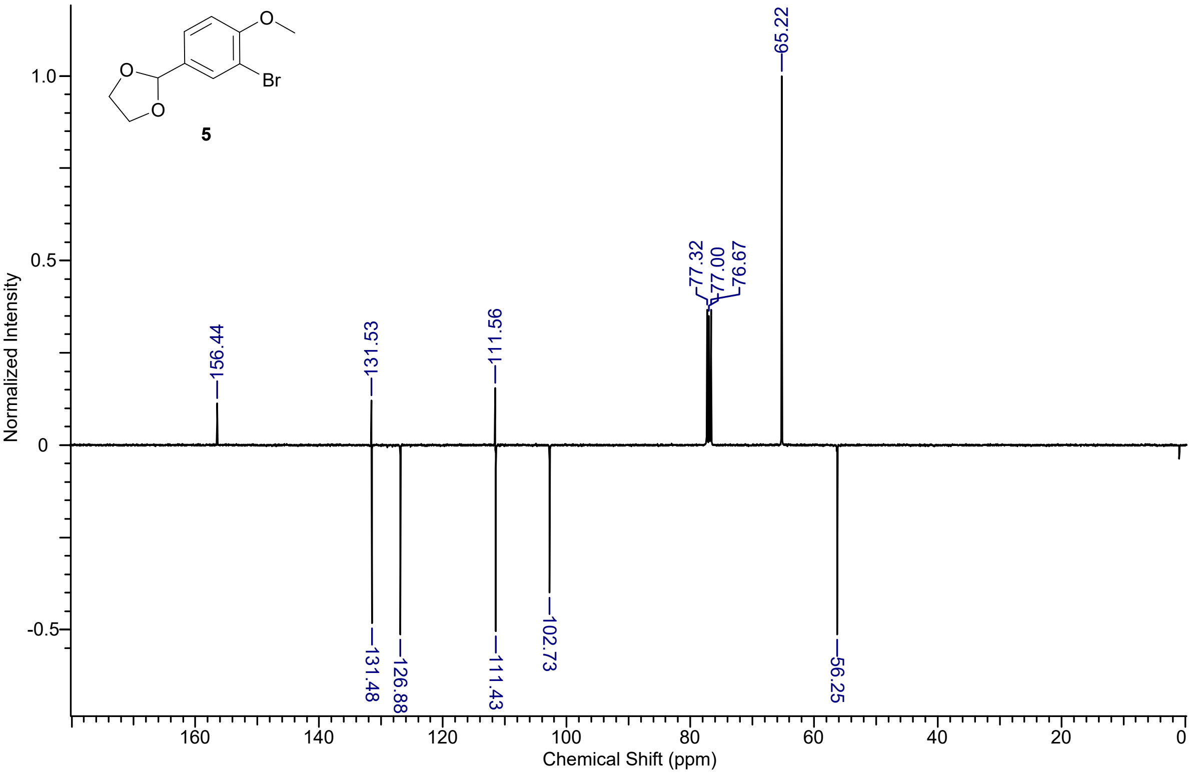

^1H and ^{13}C NMR spectra of compound **6**.

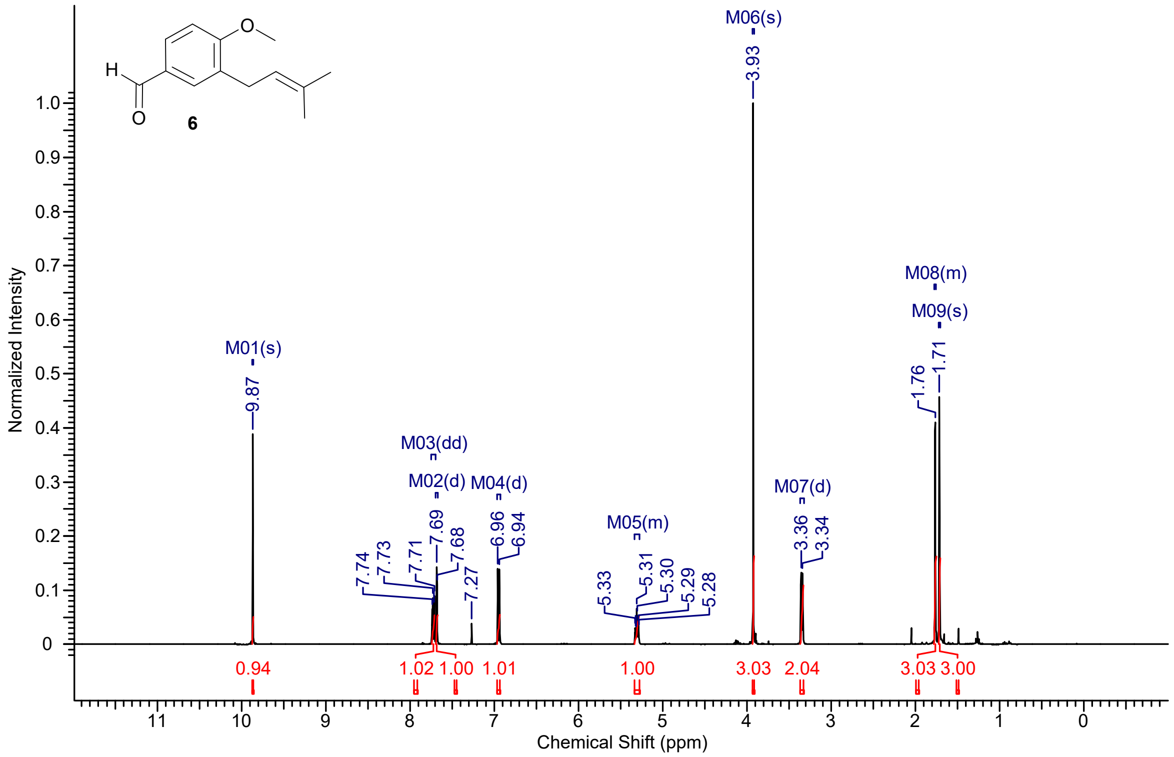

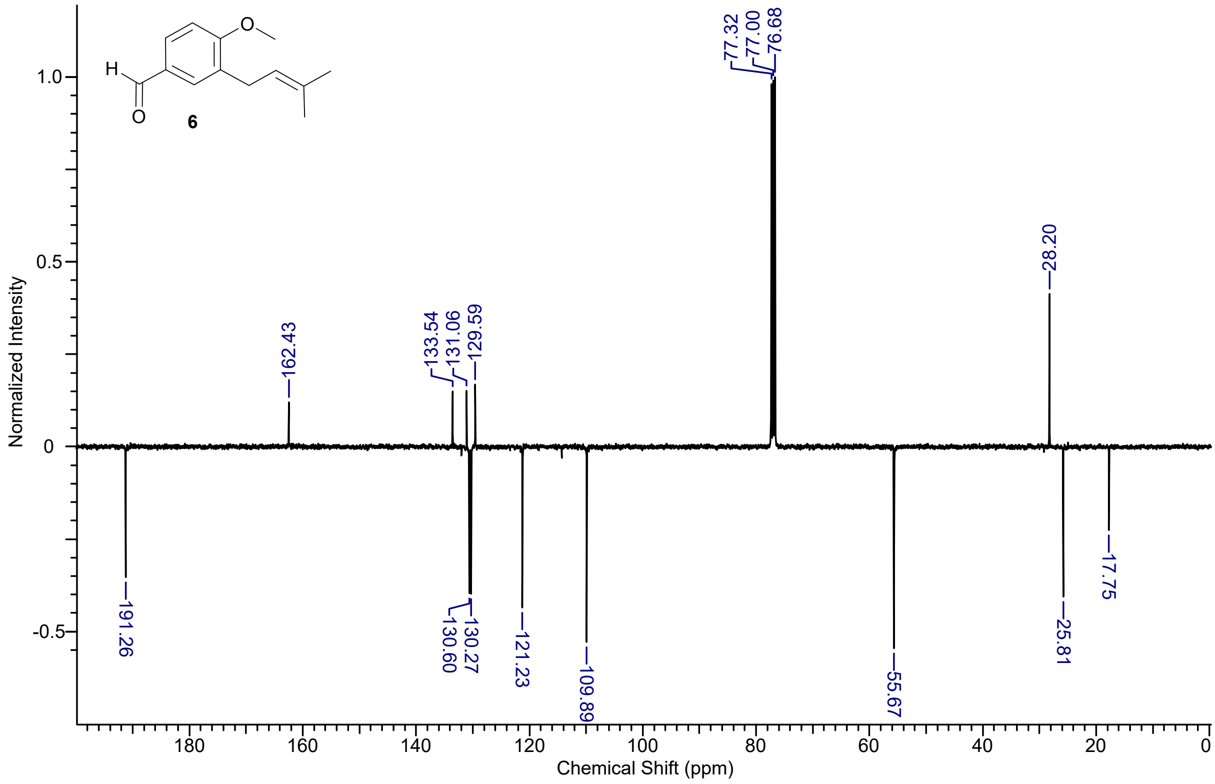

^{1}H and ^{13}C NMR spectra of compound **7**.

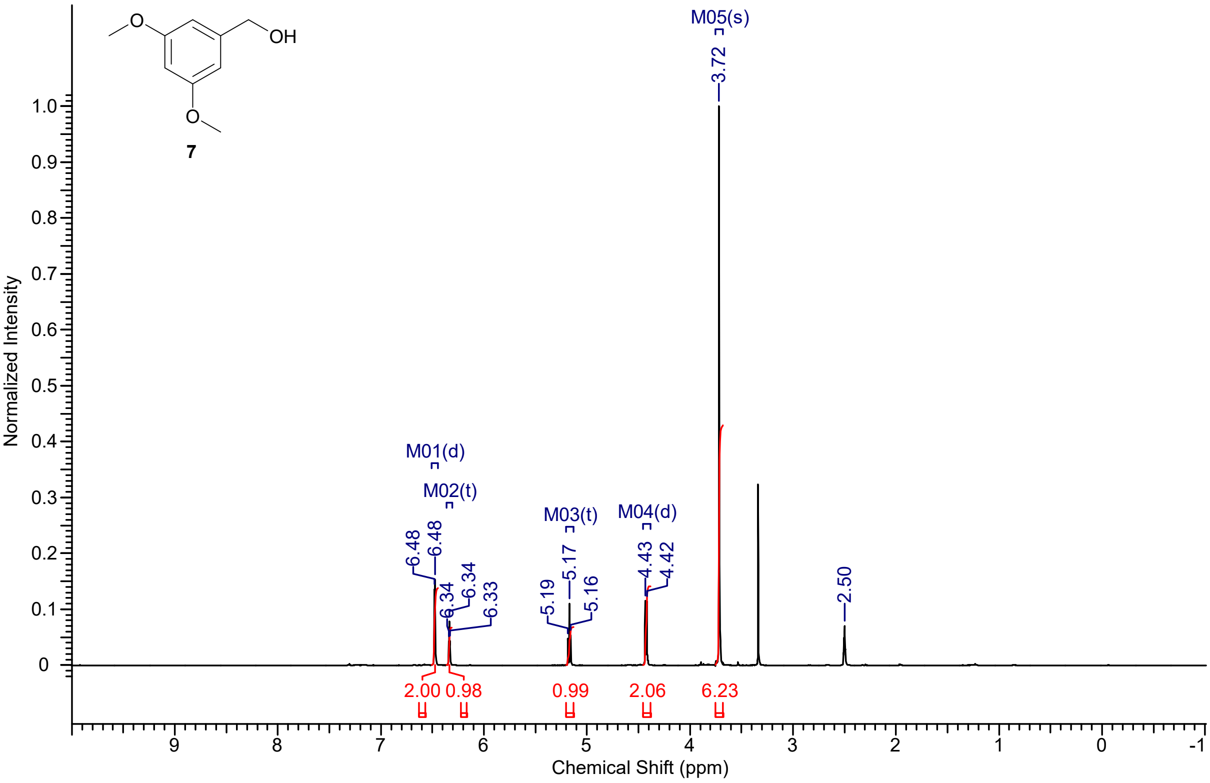

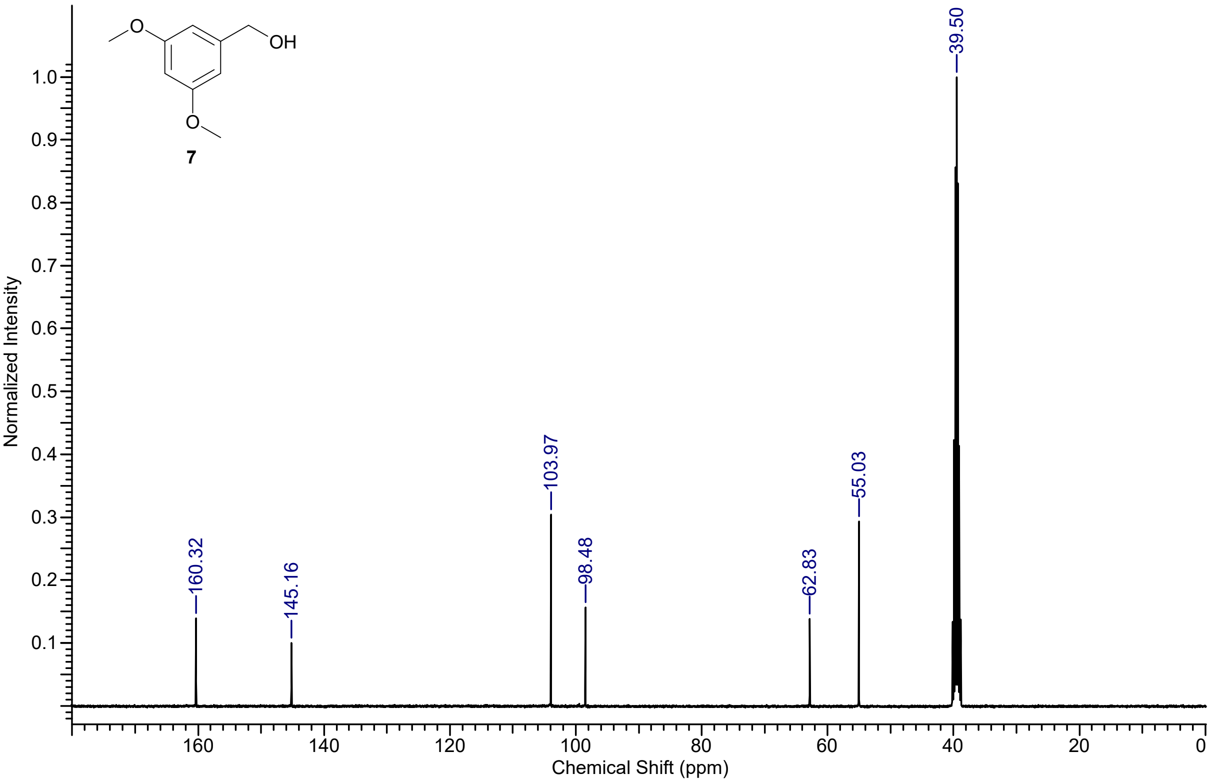

^{1}H and ^{13}C NMR spectra of compound **8**.

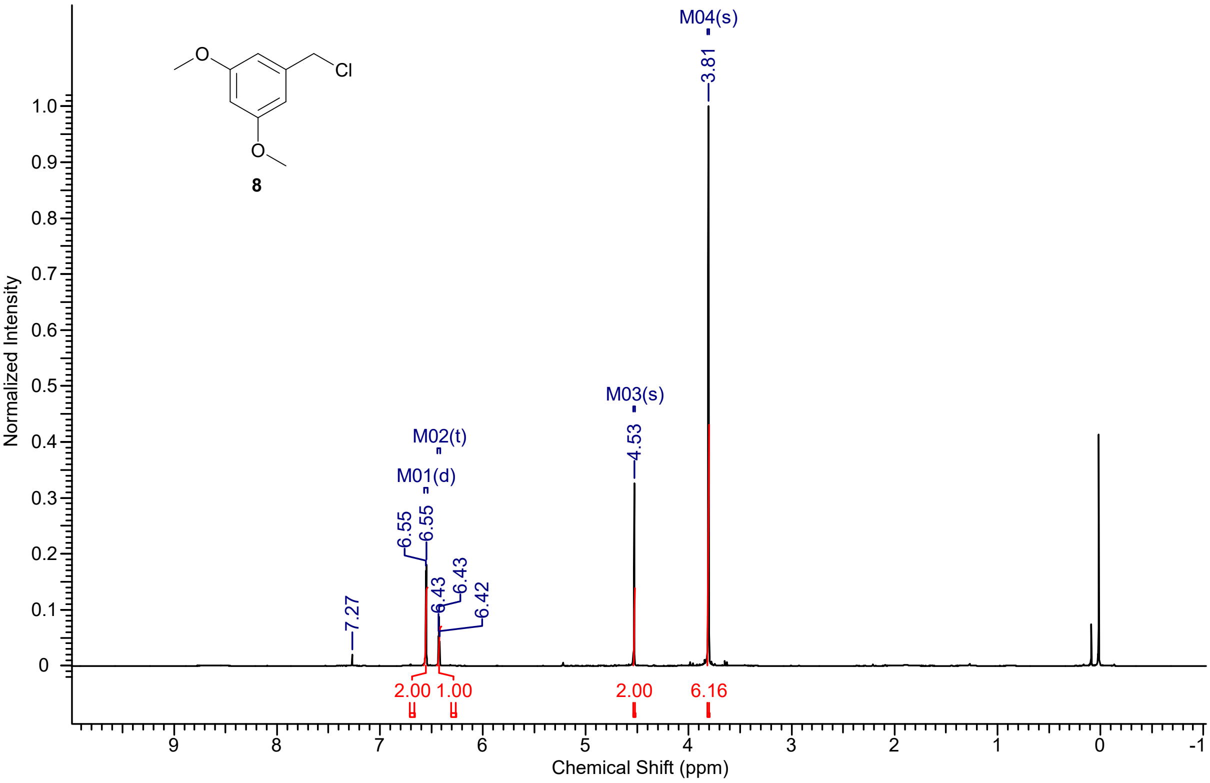

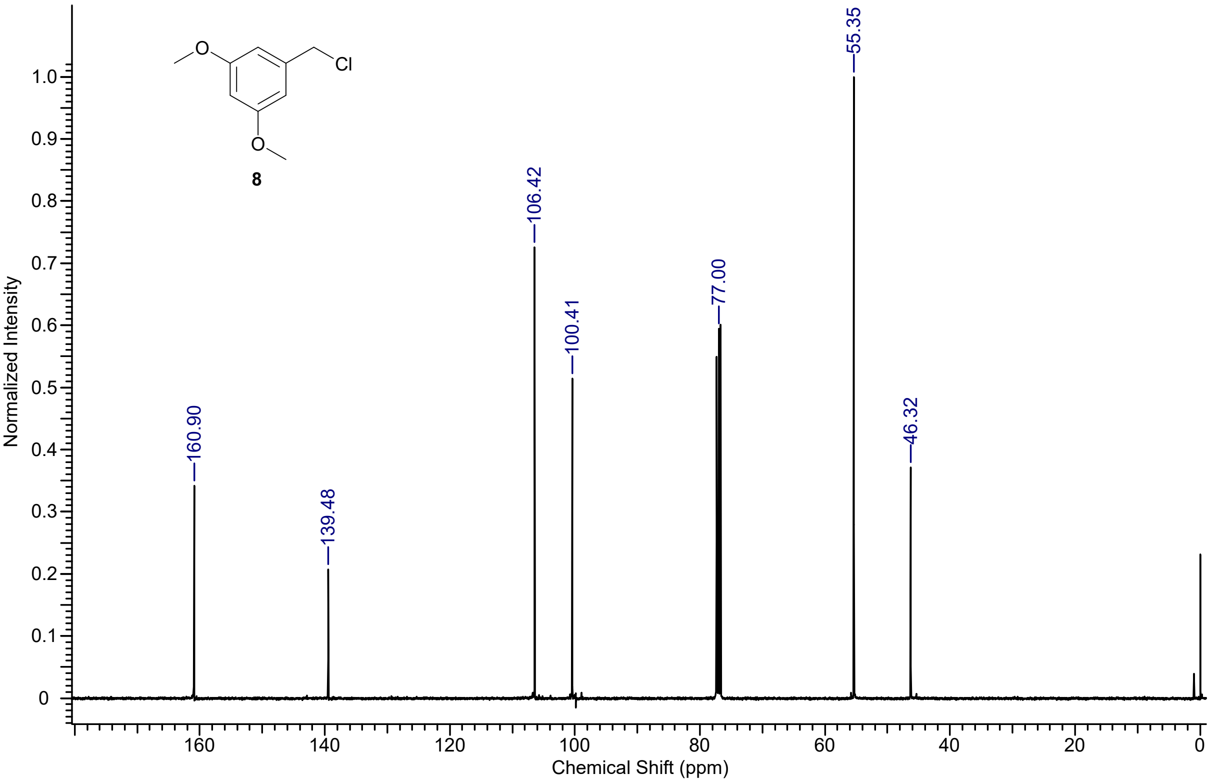

^{1}H and ^{13}C NMR spectra of compound **9**.

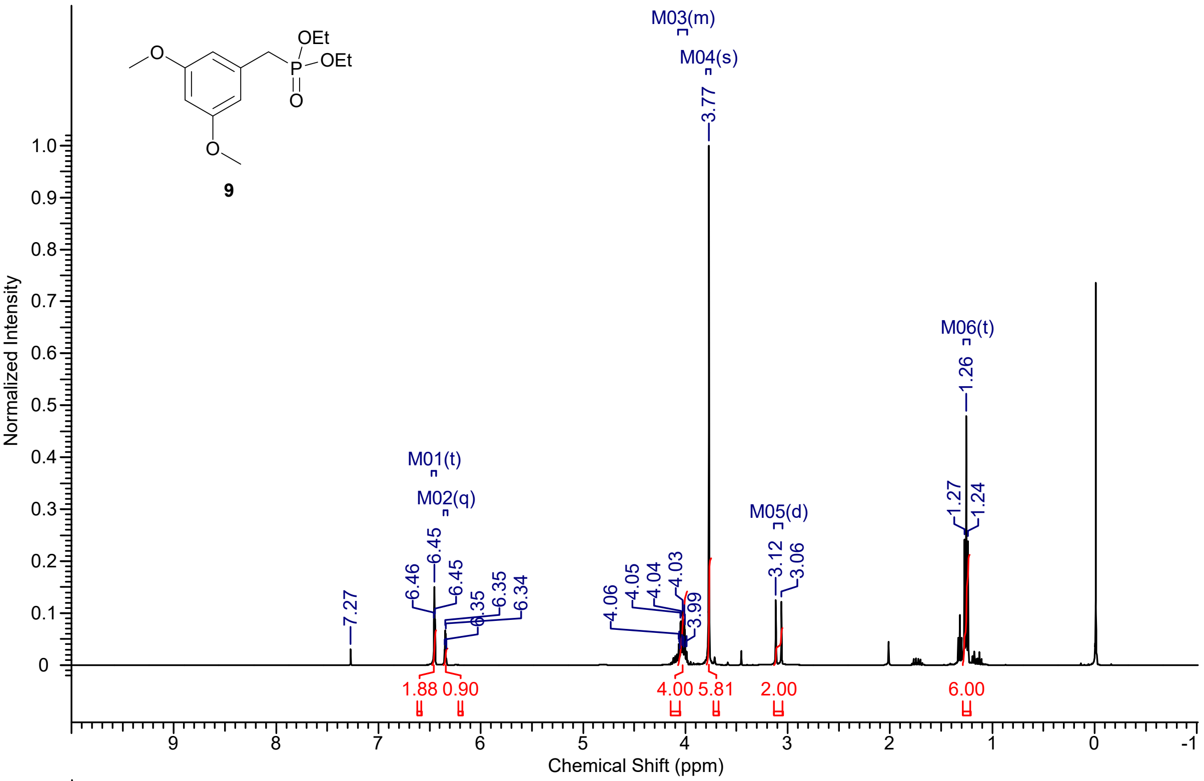

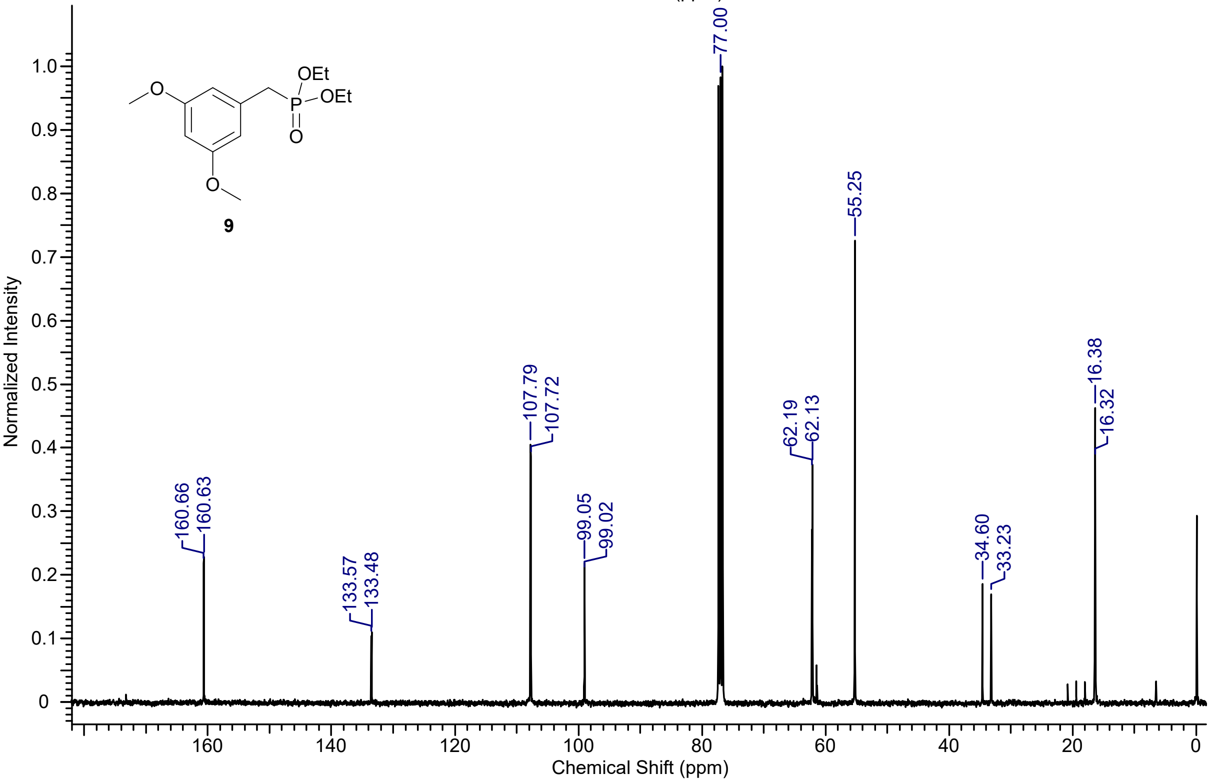

^{1}H and ^{13}C NMR spectra of compound **17**.

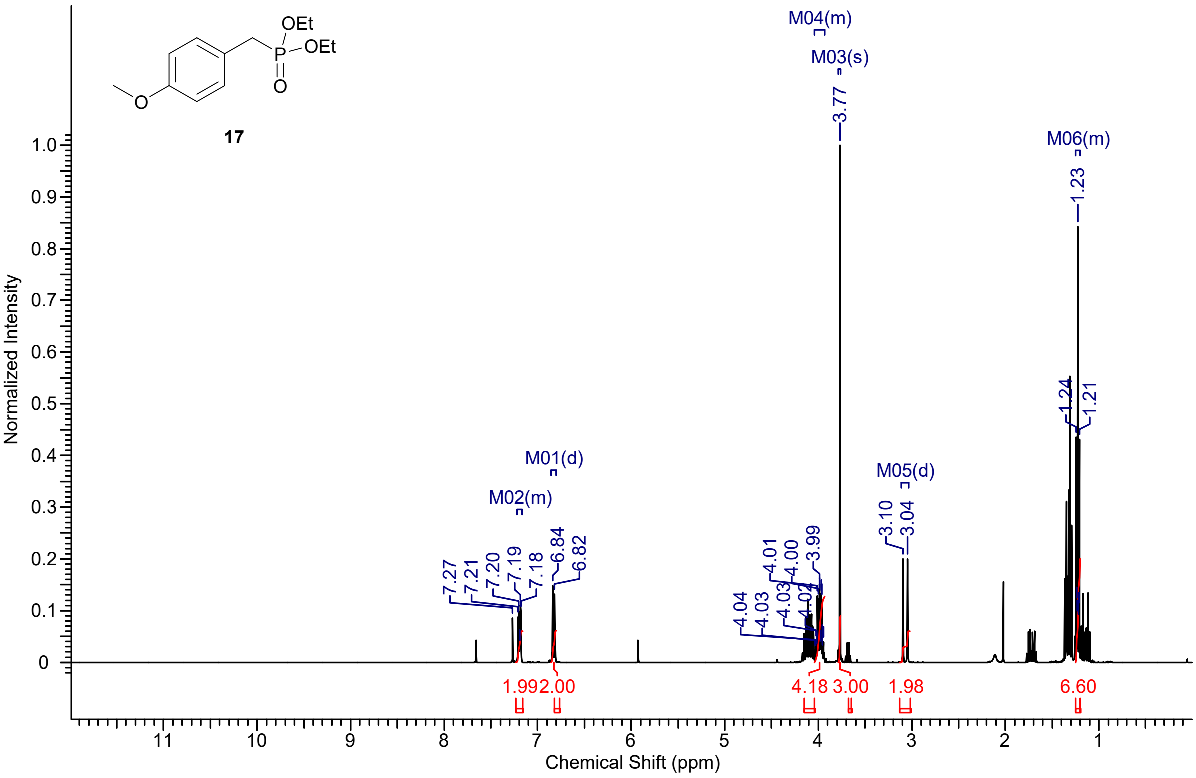

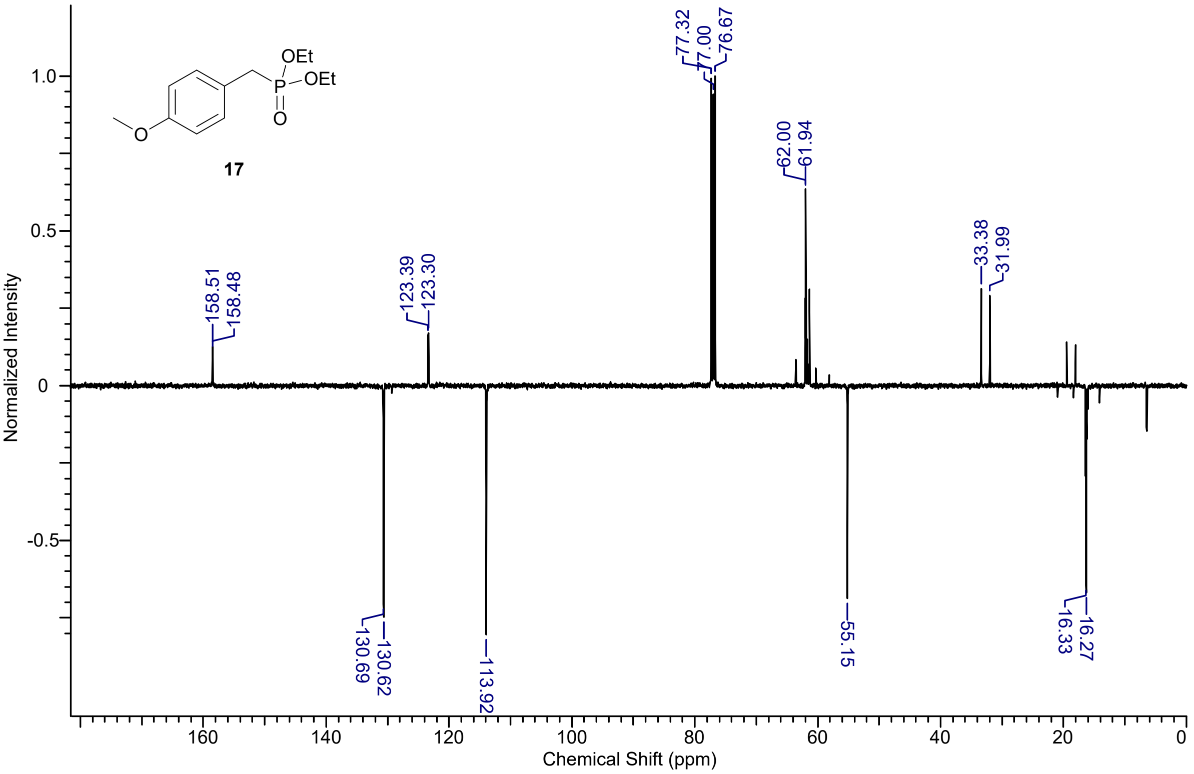

^{1}H, ^{13}C NMR and HRMS spectra of compound **1**.

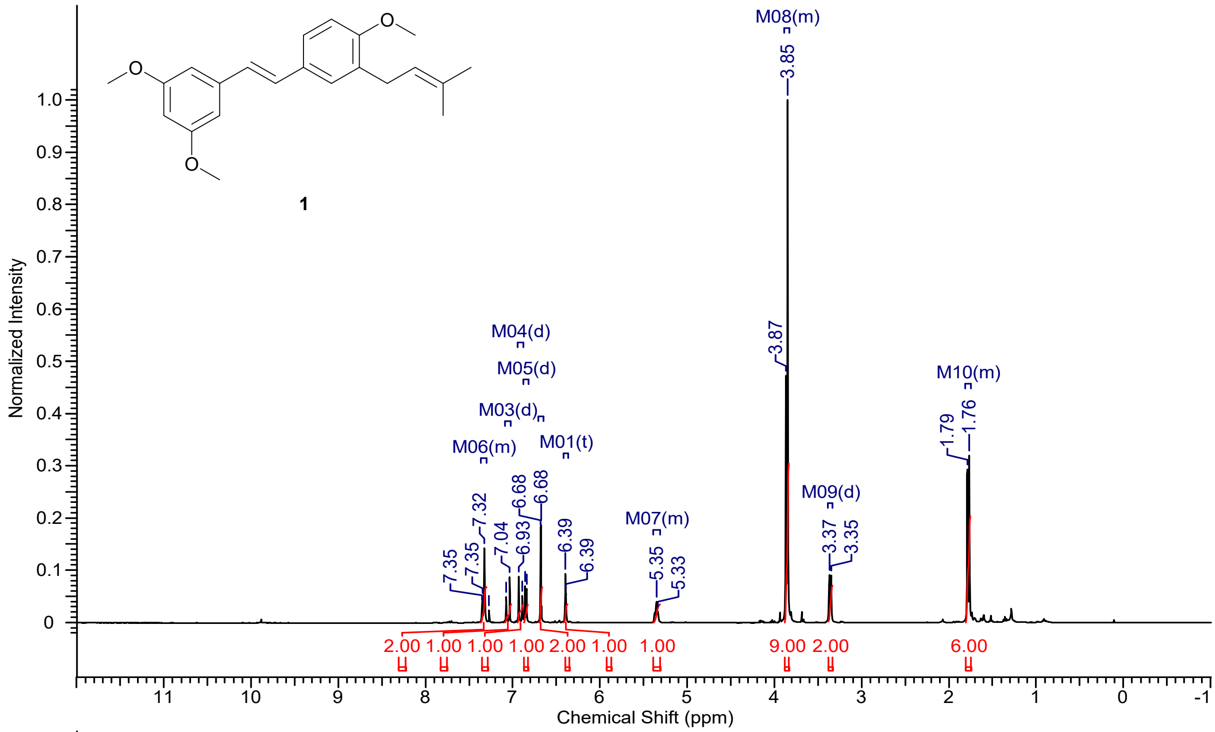

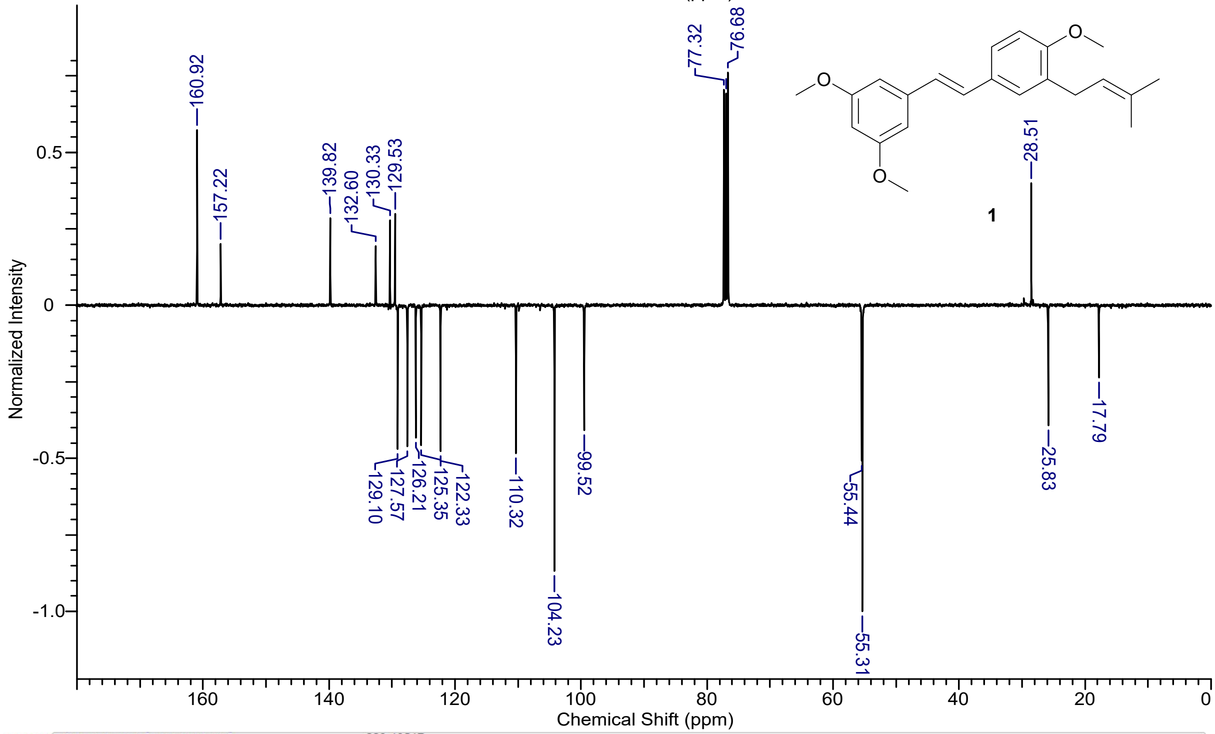

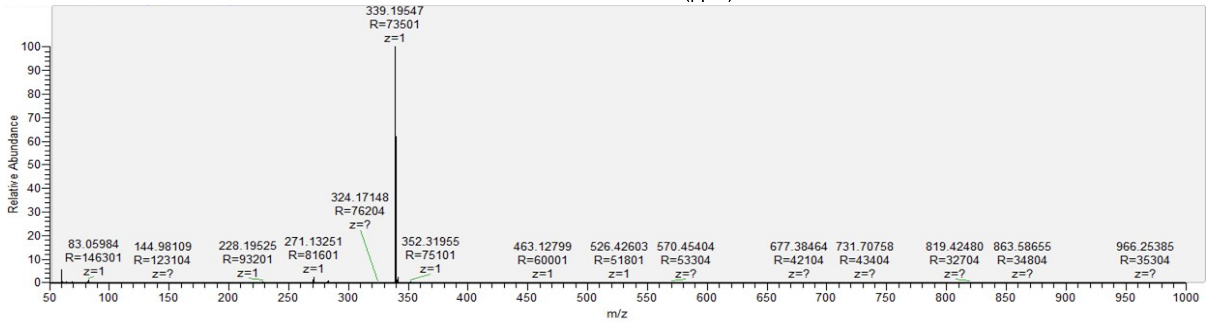

^{1}H, ^{13}C NMR and HRMS spectra of compound **3**.

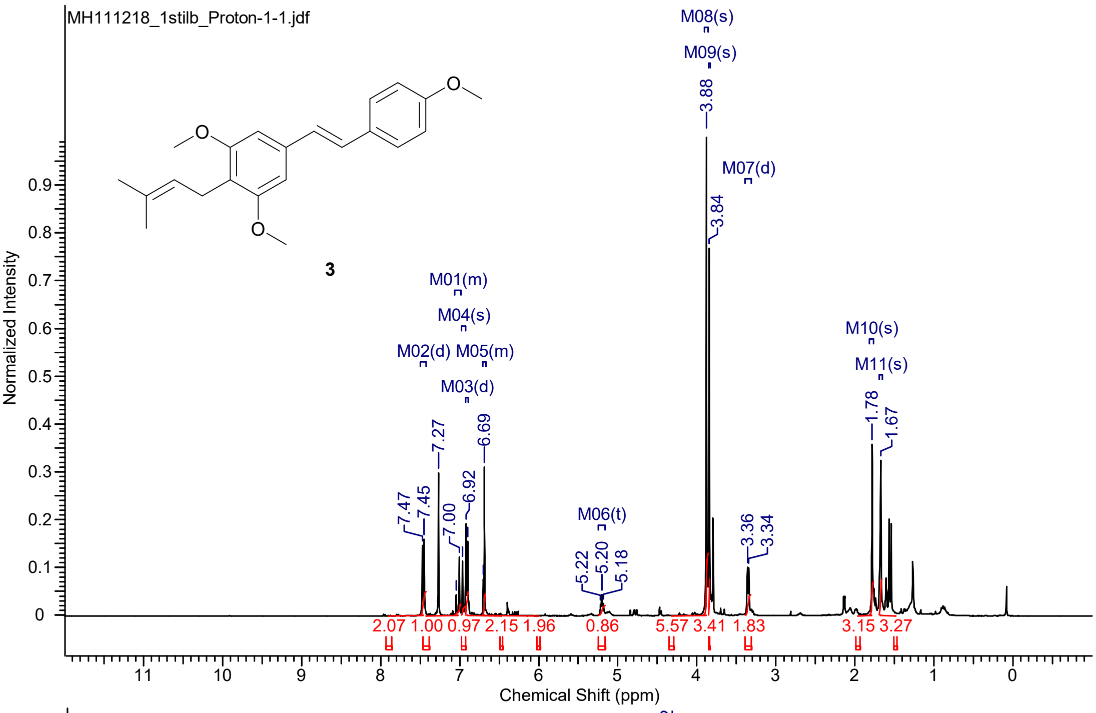

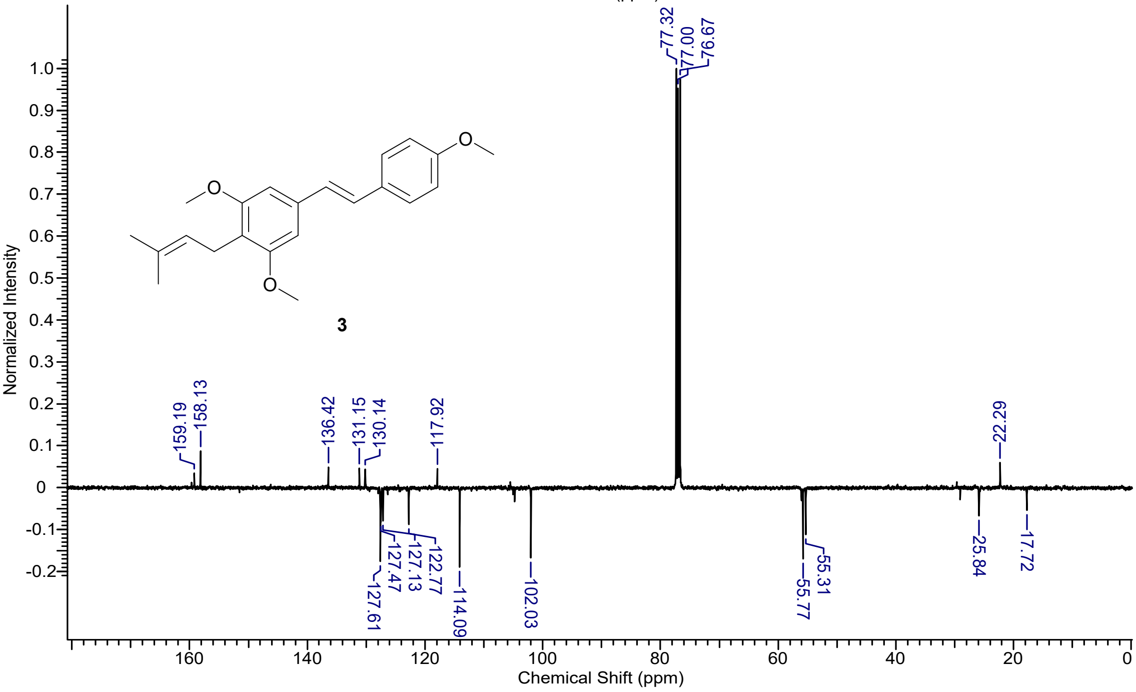

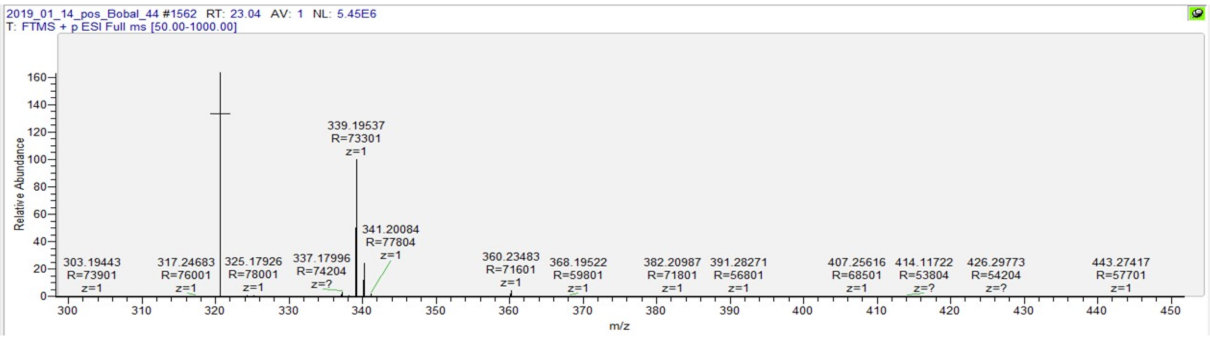

^{1}H, ^{13}C NMR and HRMS spectra of compounds **2** and **13**.

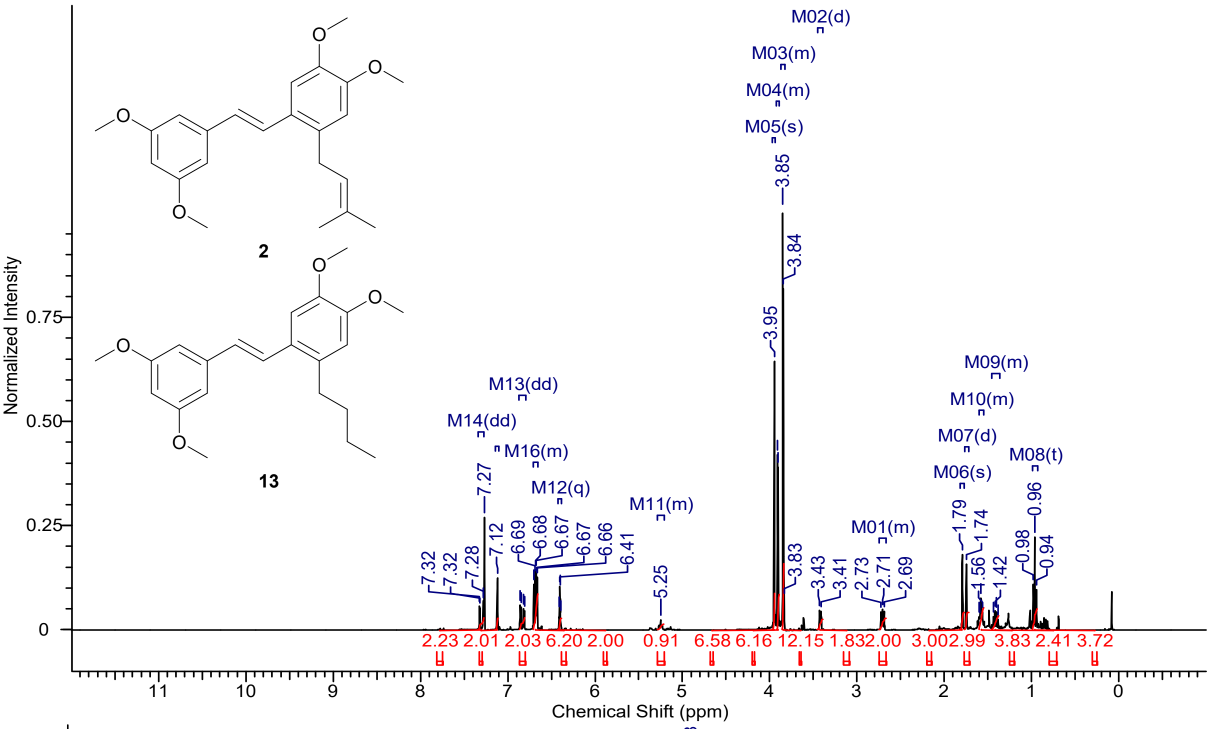

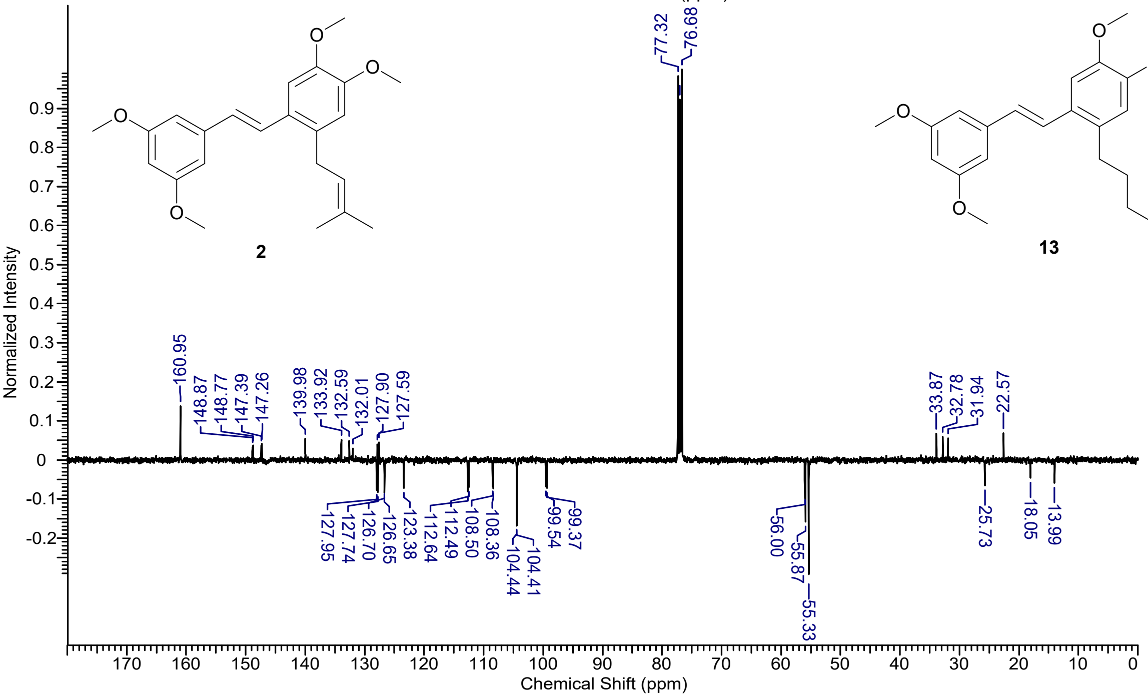

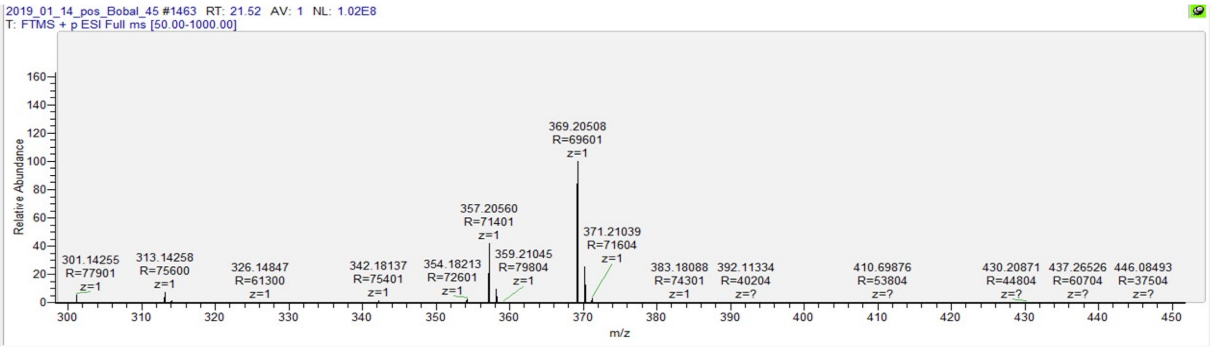

^{1}H, ^{13}C NMR and HRMS spectra of compound **4**.

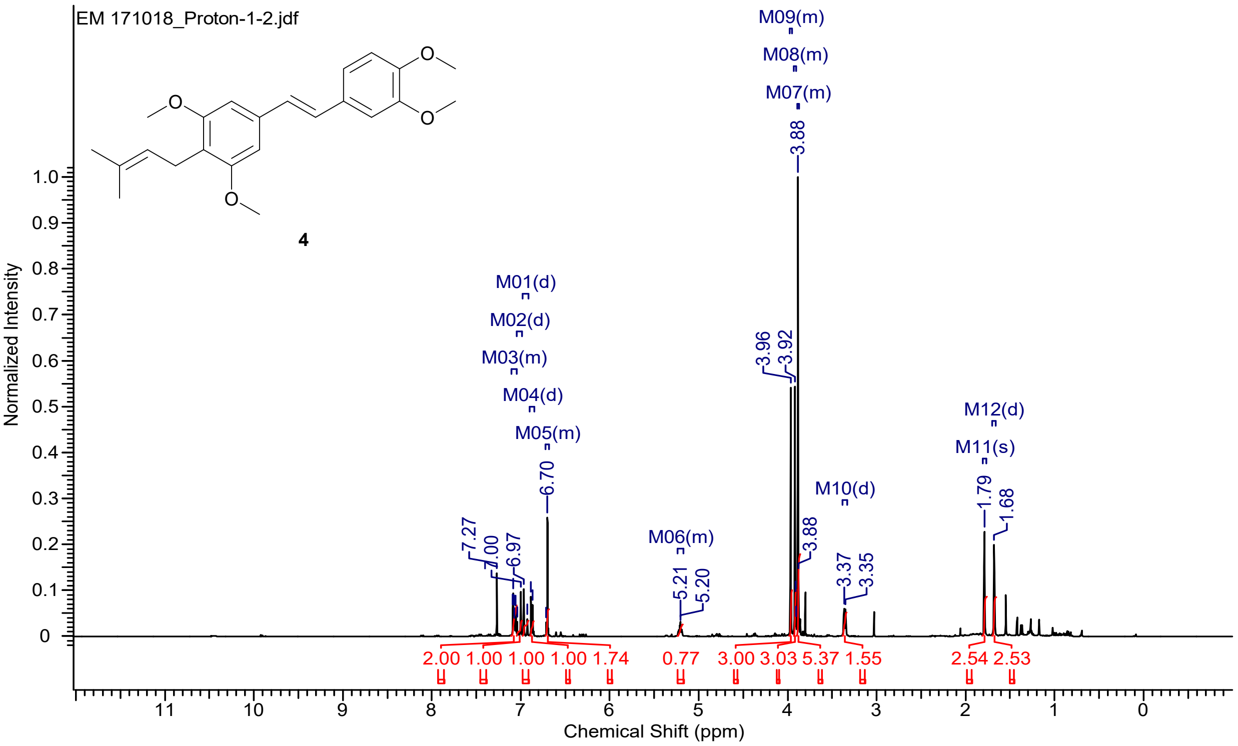

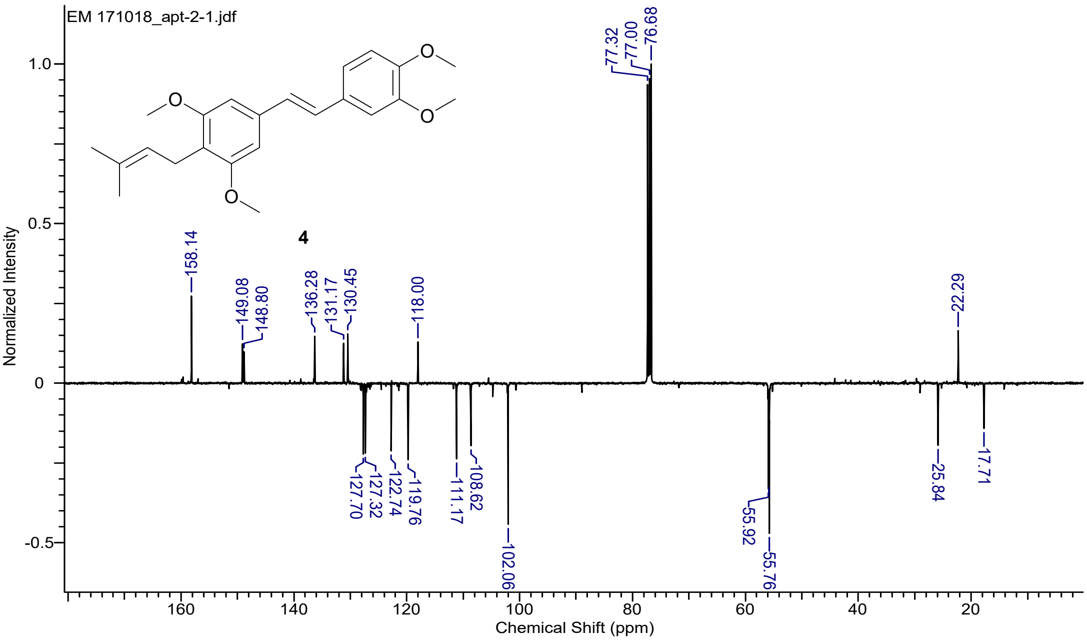

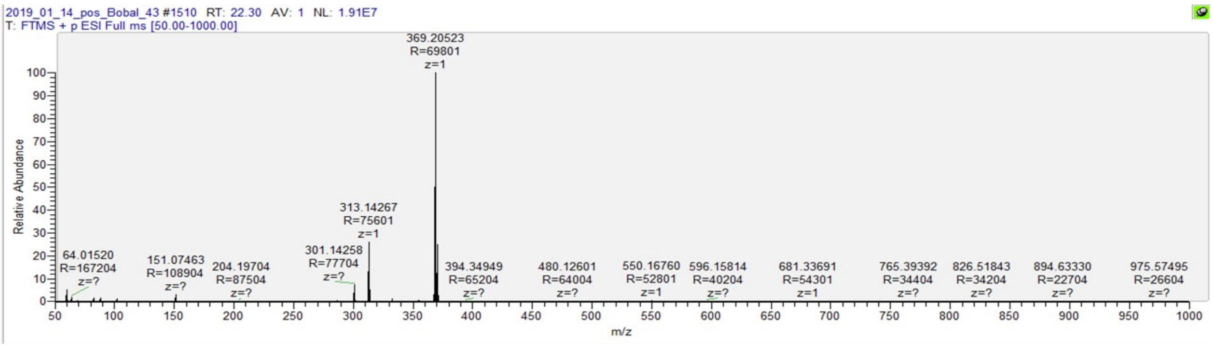

^1H, ^{13}C NMR and HRMS spectra of compound **21**.

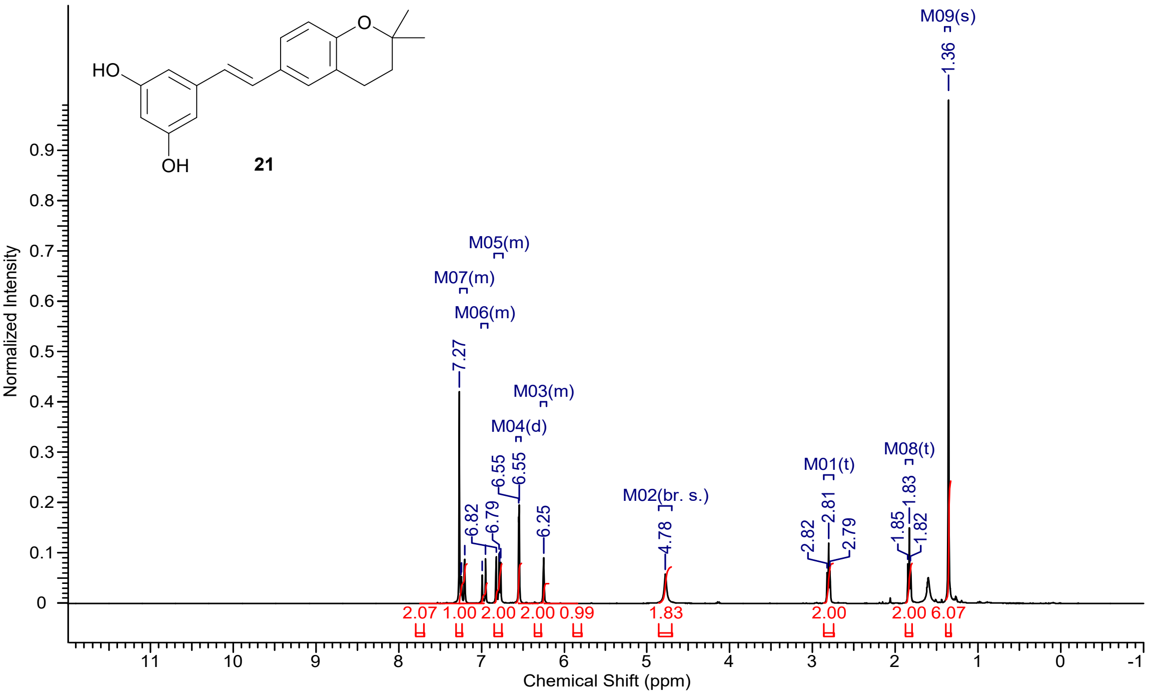

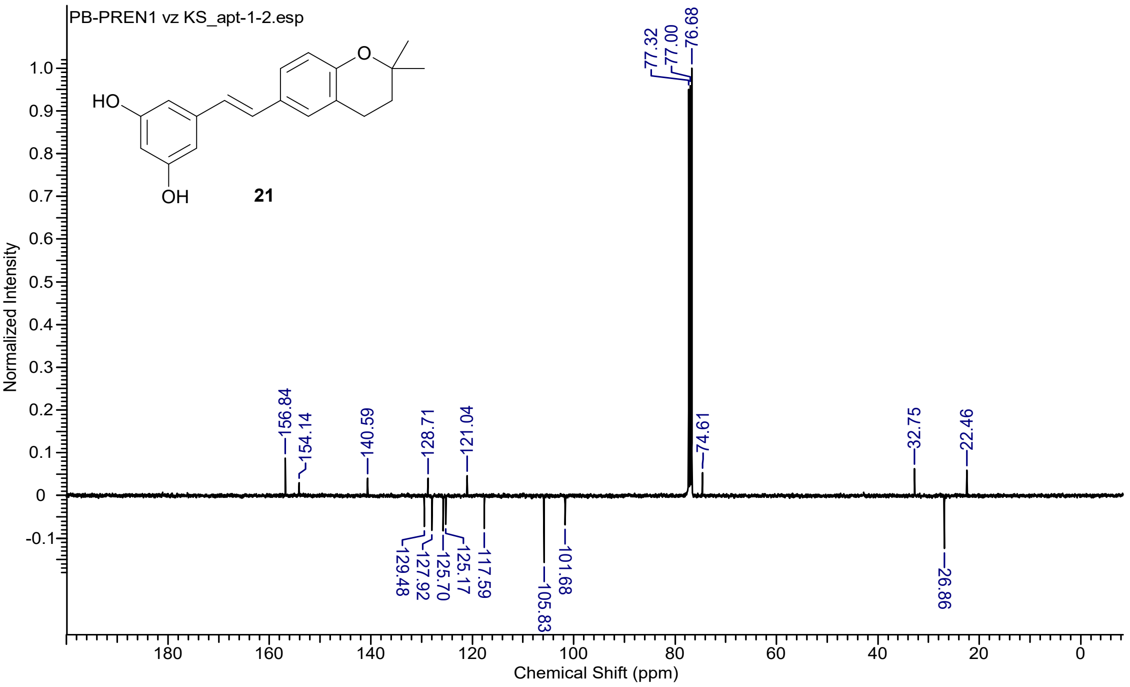

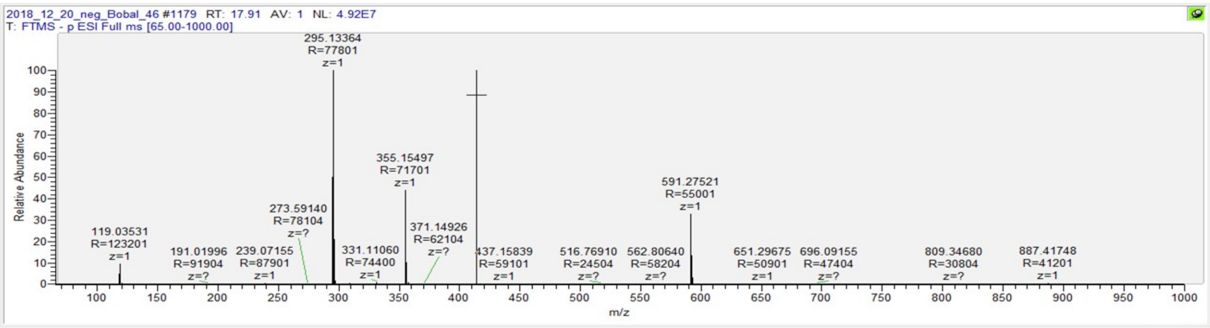

^{1}H, ^{13}C NMR and HRMS spectra of compound **22**.

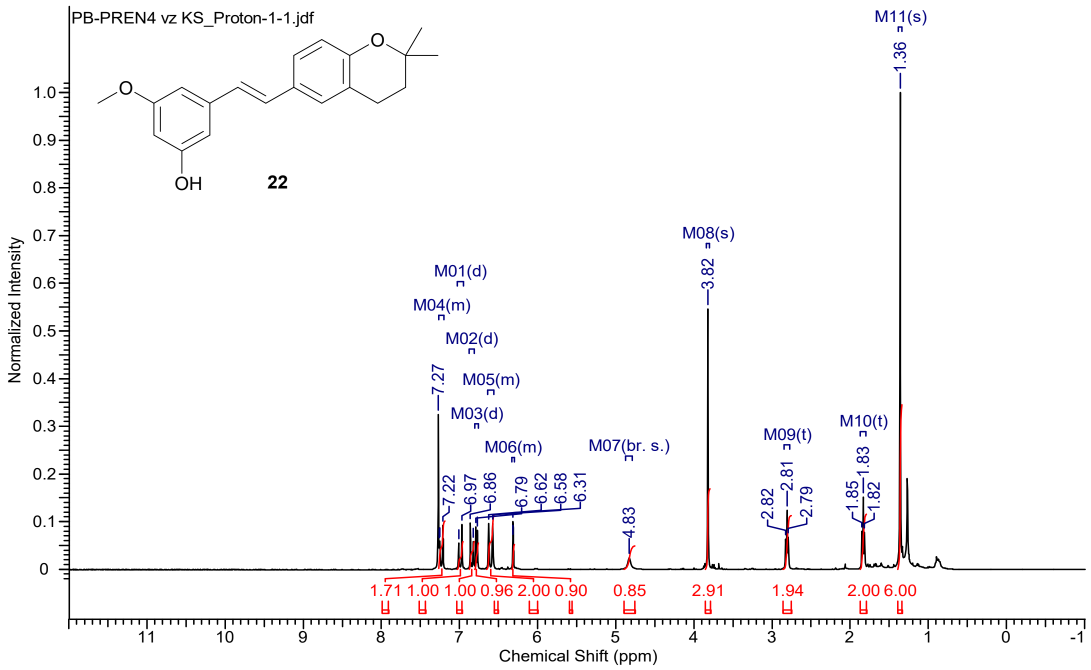

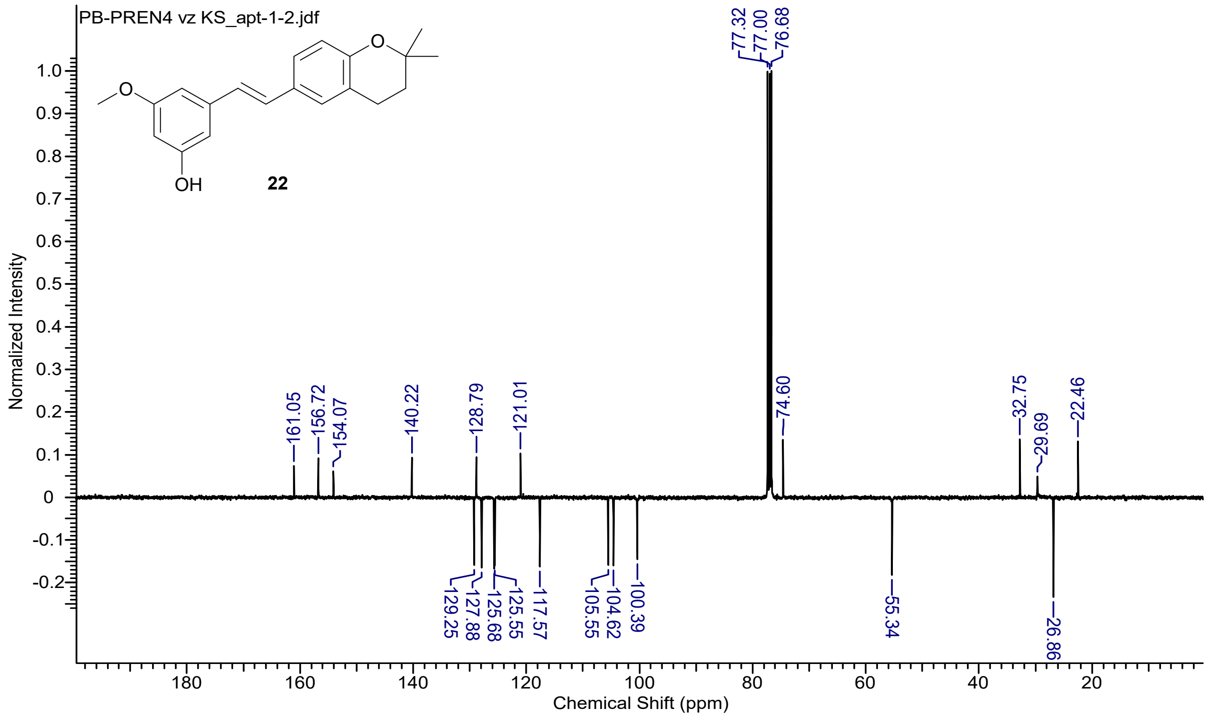

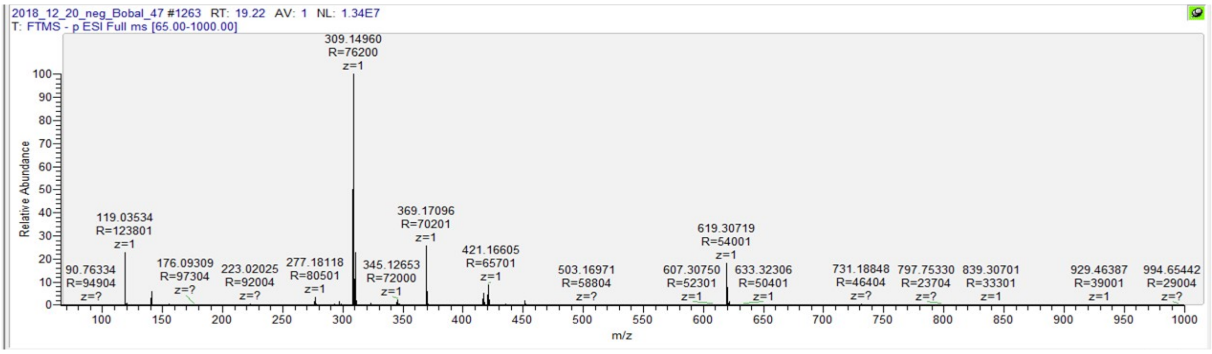

^{1}H, ^{13}C NMR and HRMS spectra of compound **23**.

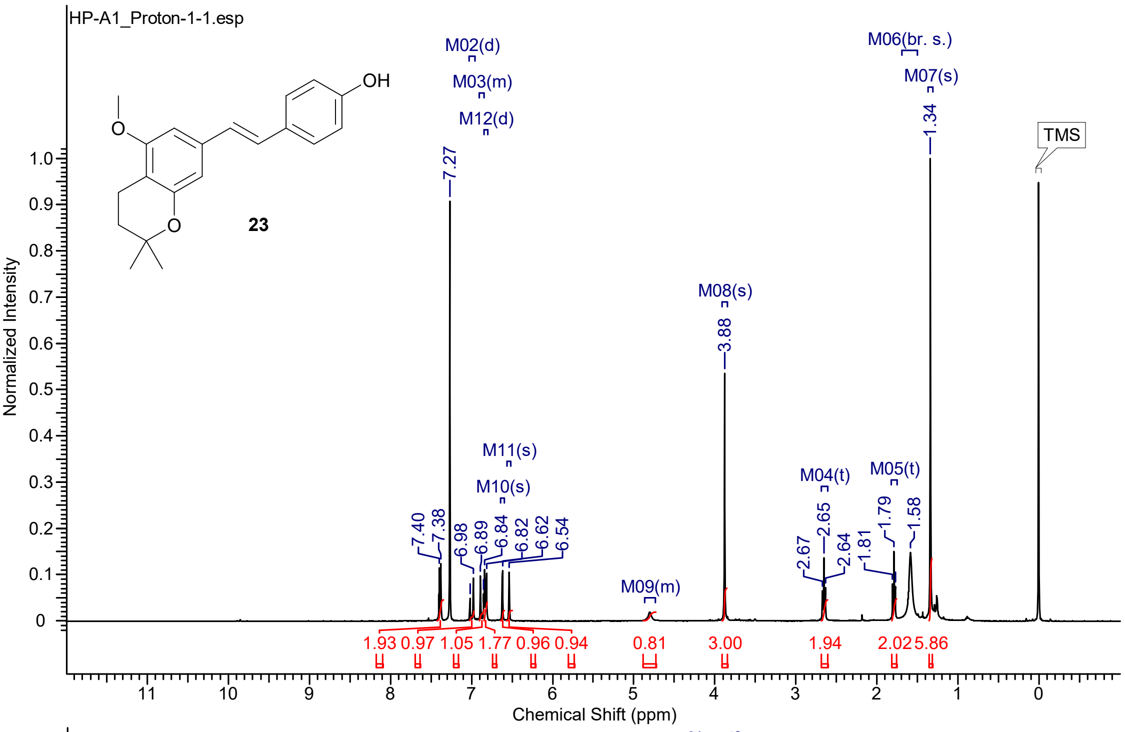

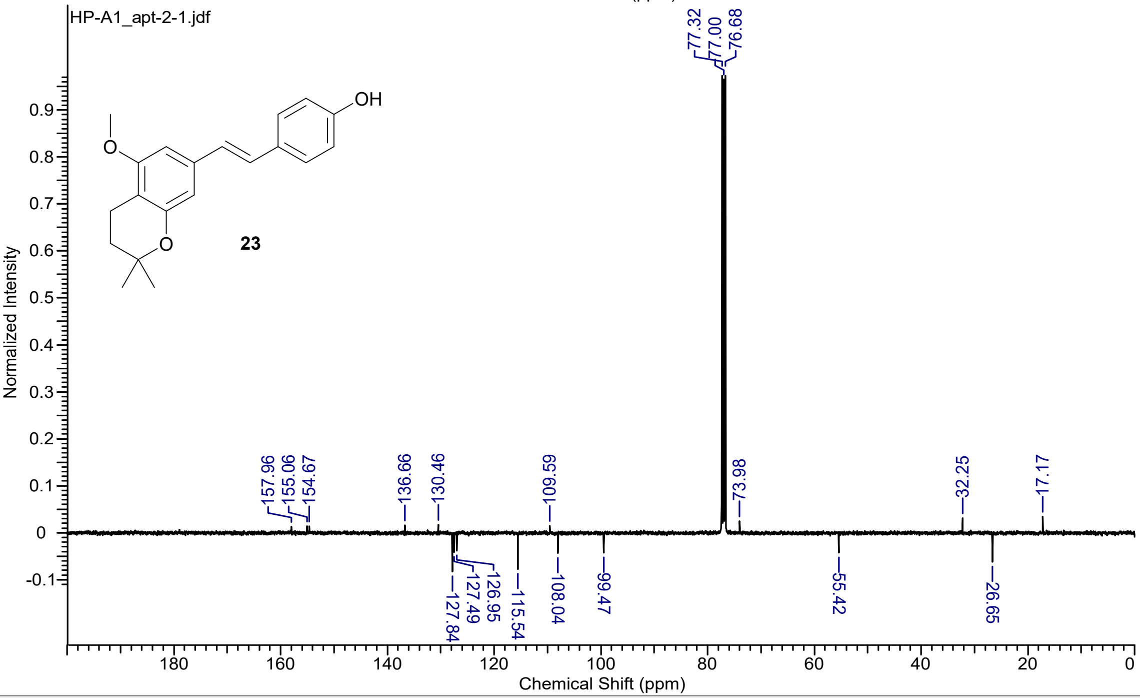

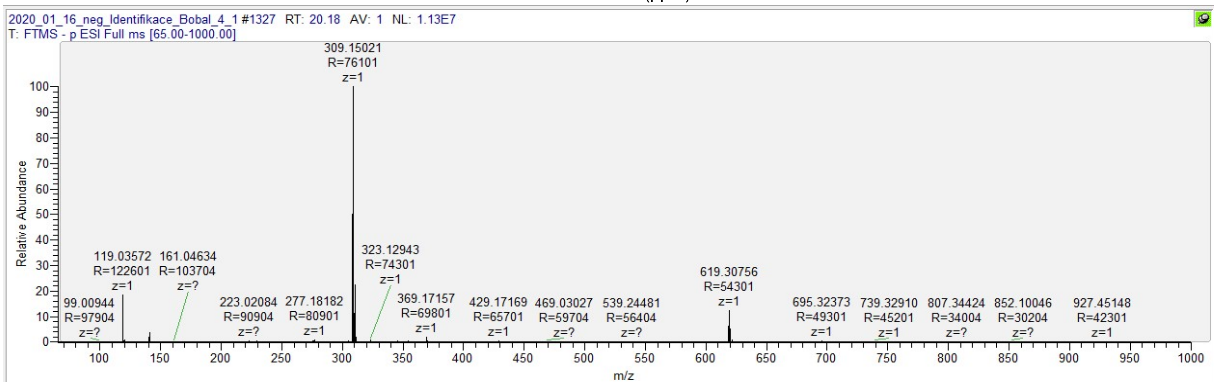

^{1}H, ^{13}C NMR and HRMS spectra of compound **24**.

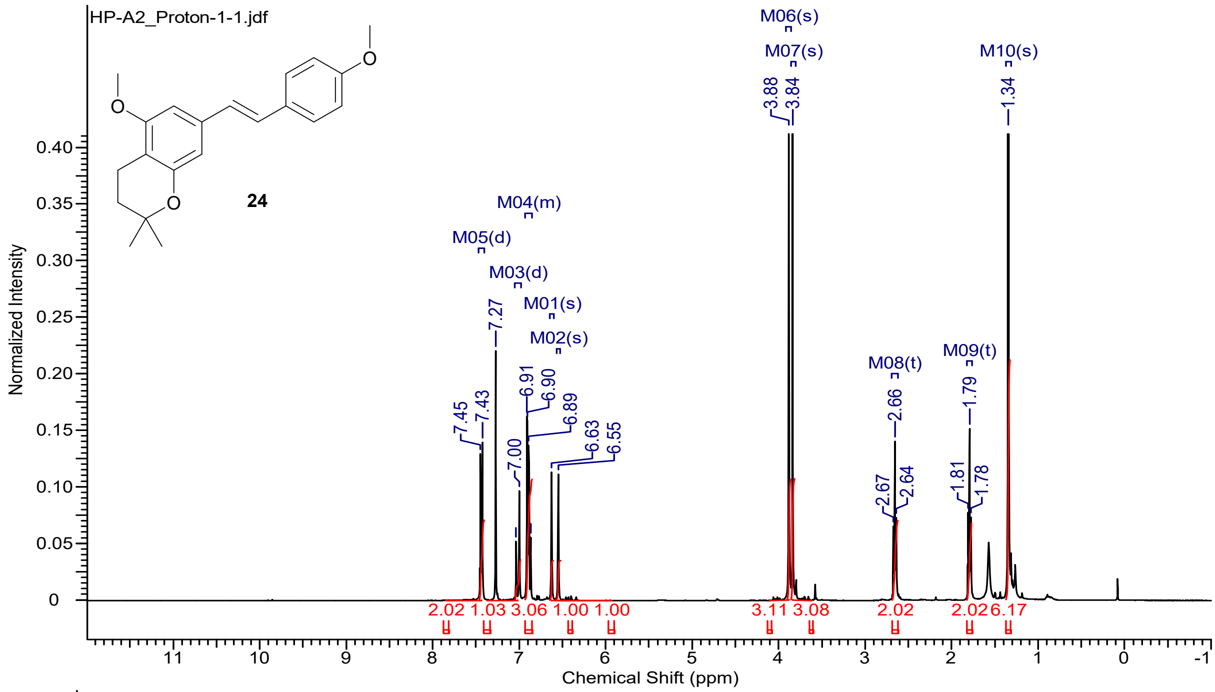

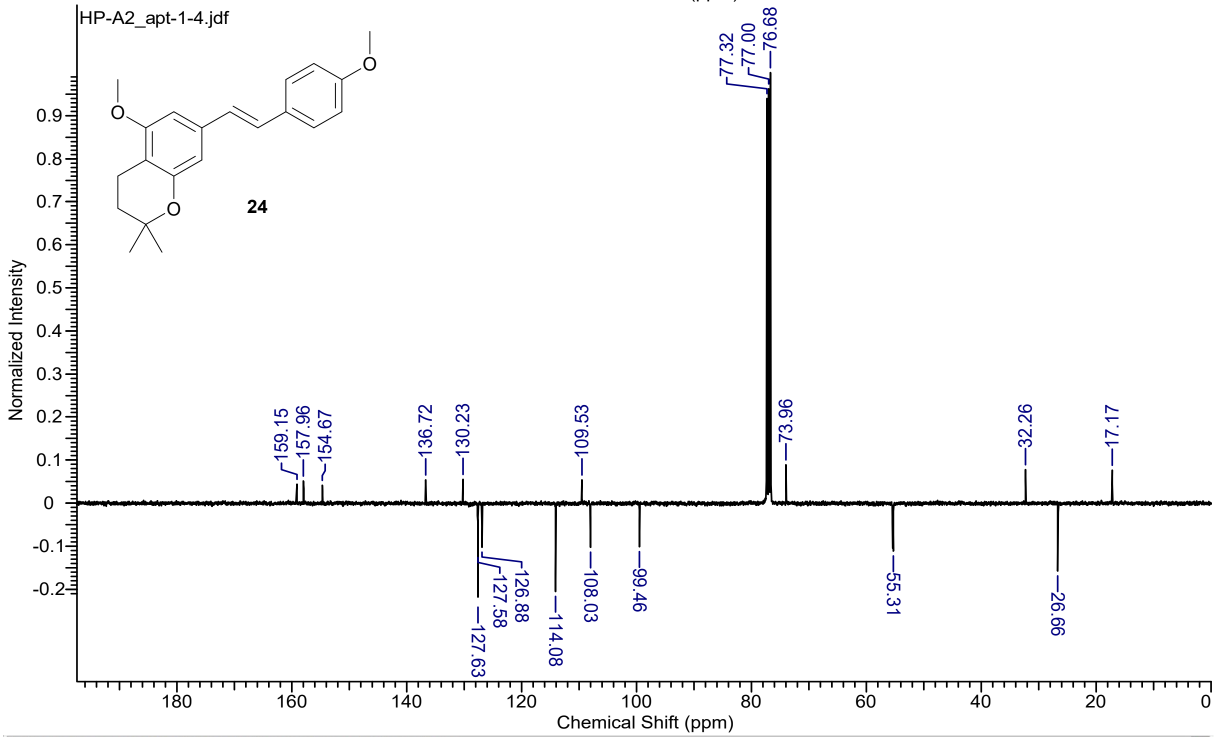

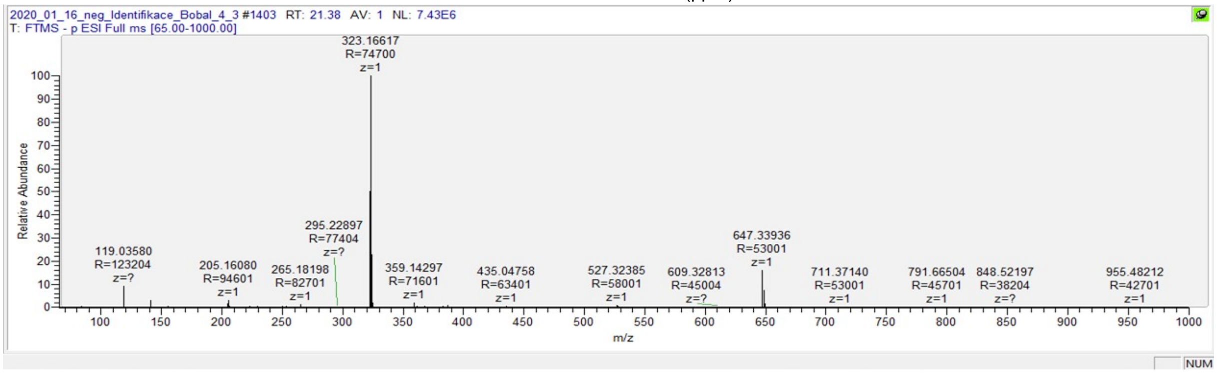

^{1}H, ^{13}C NMR and HRMS spectra of compound **25**.

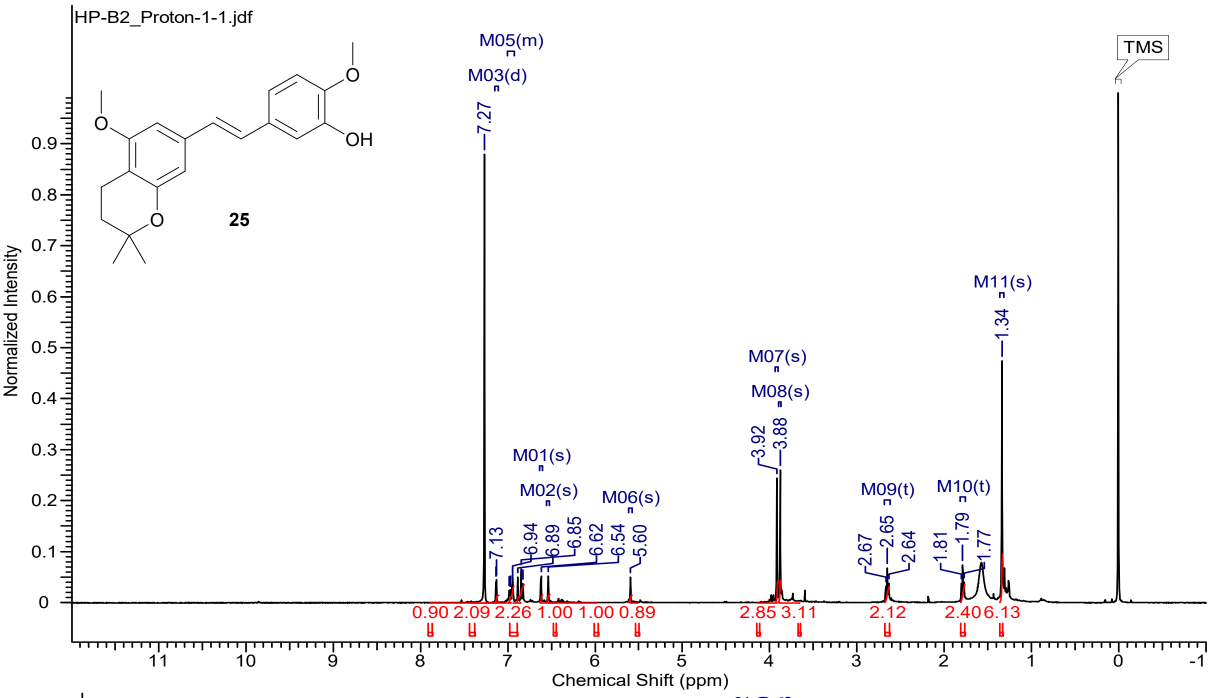

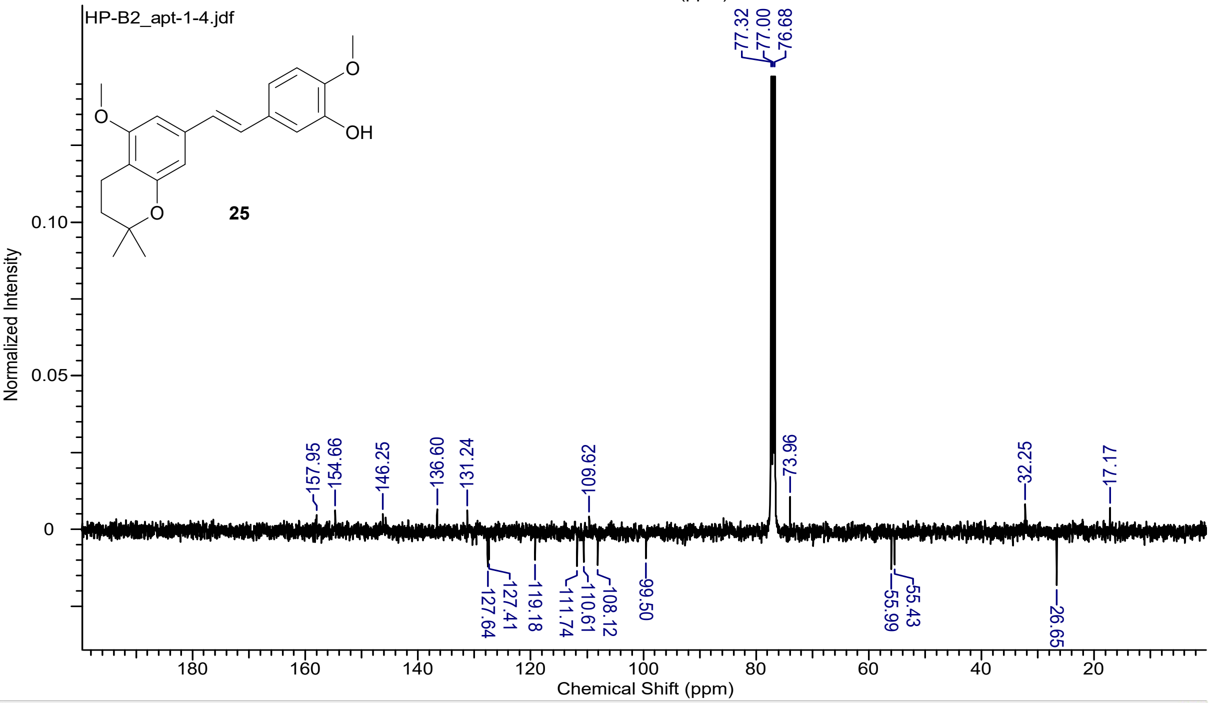

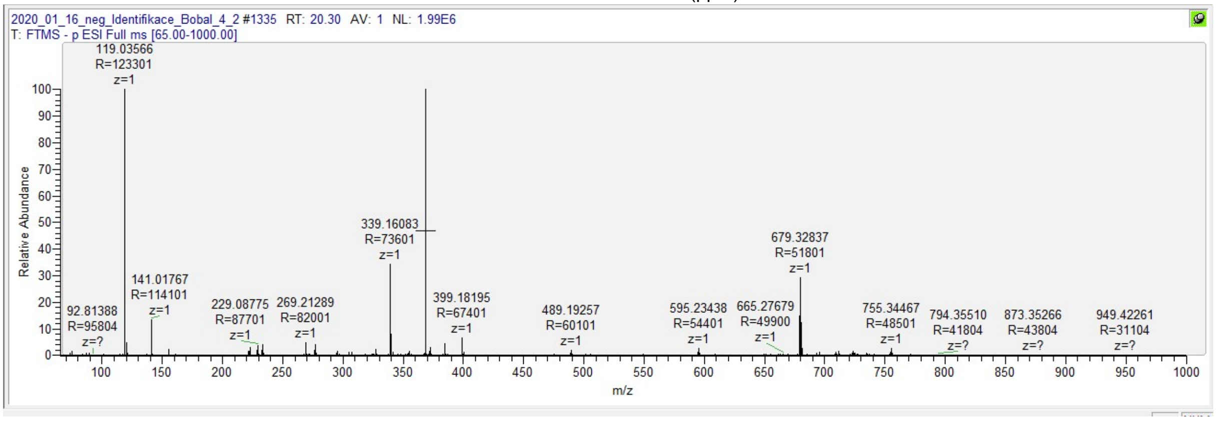

^{1}H, ^{13}C NMR and HRMS spectra of compound **26**.

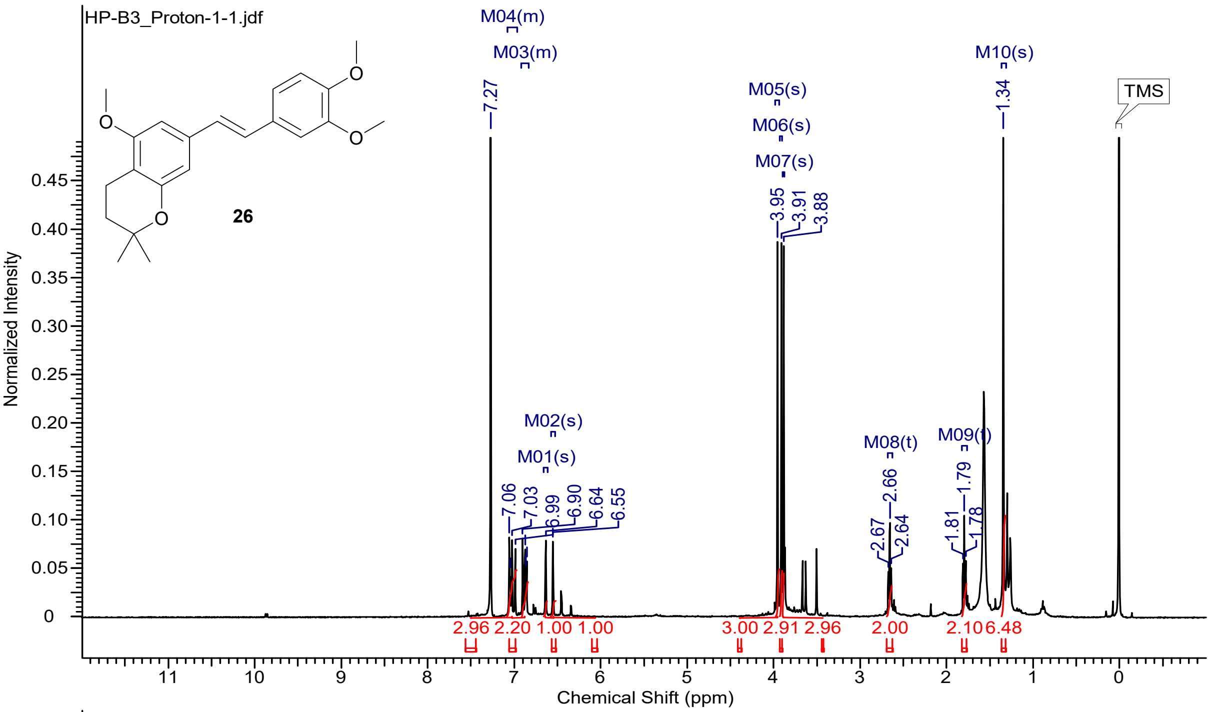

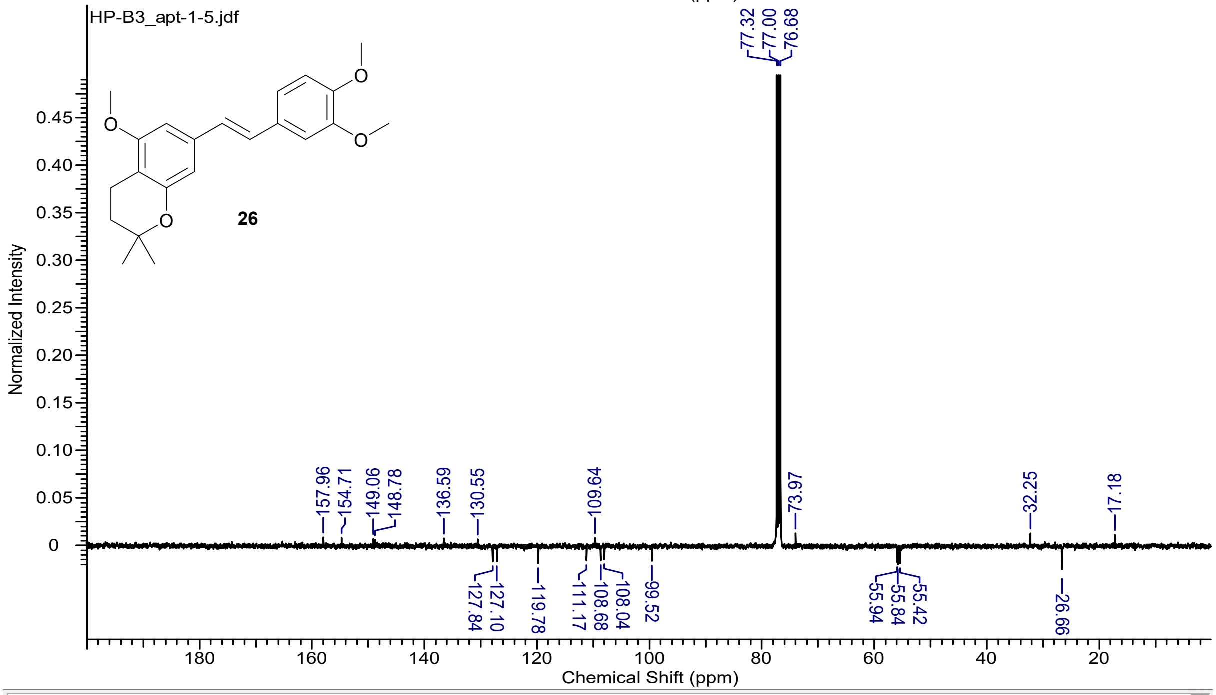

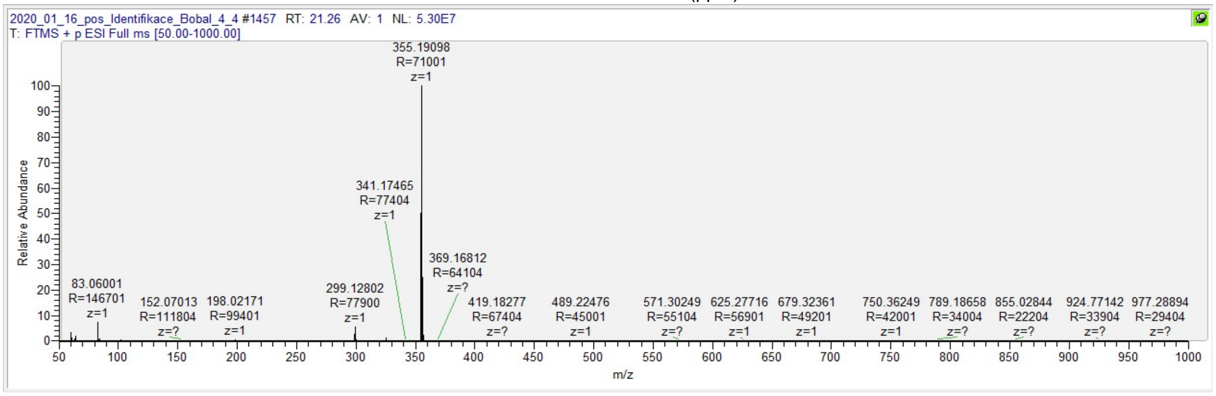

Supplement: RA-012-D2RA00441K-s001 [file RA-012-D2RA00441K-s001.pdf]
